# Pneumococcal Capsule Synthesis Locus *cps* as Evolutionary Hotspot with Potential to Generate Novel Serotypes by Recombination

**DOI:** 10.1093/molbev/msx173

**Published:** 2017-06-08

**Authors:** Rafał J. Mostowy, Nicholas J. Croucher, Nicola De Maio, Claire Chewapreecha, Susannah J. Salter, Paul Turner, David M. Aanensen, Stephen D. Bentley, Xavier Didelot, Christophe Fraser

**Affiliations:** 1 Department of Infectious Disease Epidemiology, School of Public Health, Imperial College London, London, United Kingdom; 2 Nuffield Department of Medicine, University of Oxford, Oxford, United Kingdom; 3 Institute for Emerging Infections, Oxford Martin School, Oxford, United Kingdom; 4 Department of Medicine, University of Cambridge, Cambridge, United Kingdom; 5 Bioinformatics and Systems Biology Program, School of Bioresources and Technology, King Mongkut’s University of Technology Thonburi, Bangkok, Thailand; 6 The Wellcome Trust Sanger Institute, Wellcome Trust Genome Campus, Hinxton, Cambridge, United Kingdom; 7 Nuffield Department of Medicine, Centre for Tropical Medicine and Global Health, University of Oxford, Oxford, United Kingdom; 8 Cambodia-Oxford Medical Research Unit, Angkor Hospital for Children, Siem Reap, Cambodia; 9 Centre for Genomic Pathogen Surveillance, Wellcome Genome Campus, Hinxton, Cambridge, United Kingdom; 10 Nuffield Department of Medicine, Li Ka Shing Centre for Health Information and Discovery, Oxford Big Data Institute, University of Oxford, Oxford, United Kingdom

**Keywords:** polysaccharide diversity, conjugate vaccine, pneumococcal disease, evolutionary dynamics, epidemiology, next-generation sequencing

## Abstract

Diversity of the polysaccharide capsule in *Streptococcus pneumoniae***—**main surface antigen and the target of the currently used pneumococcal vaccines**—**constitutes a major obstacle in eliminating pneumococcal disease. Such diversity is genetically encoded by almost 100 variants of the capsule biosynthesis locus, *cps*. However, the evolutionary dynamics of the capsule remains not fully understood. Here, using genetic data from 4,519 bacterial isolates, we found *cps* to be an evolutionary hotspot with elevated substitution and recombination rates. These rates were a consequence of relaxed purifying selection and positive, diversifying selection acting at this locus, supporting the hypothesis that the capsule has an increased potential to generate novel diversity compared with the rest of the genome. Diversifying selection was particularly evident in the region of *wzd/wze* genes, which are known to regulate capsule expression and hence the bacterium’s ability to cause disease. Using a novel, capsule-centered approach, we analyzed the evolutionary history of 12 major serogroups. Such analysis revealed their complex diversification scenarios, which were principally driven by recombination with other serogroups and other streptococci. Patterns of recombinational exchanges between serogroups could not be explained by serotype frequency alone, thus pointing to nonrandom associations between co-colonizing serotypes. Finally, we discovered a previously unobserved mosaic serotype 39X, which was confirmed to carry a viable and structurally novel capsule. Adding to previous discoveries of other mosaic capsules in densely sampled collections, these results emphasize the strong adaptive potential of the bacterium by its ability to generate novel antigenic diversity by recombination.

## Introduction

*Streptococcus pneumoniae* is a human bacterial commensal and pathogen, estimated to be the cause of death in over 500,000 children under 5 years of age each year worldwide (WHO 2012). The bacterium’s capacity to cause disease is associated with its possession of several virulence factors, of which the most important is the surface polysaccharide capsule (Briles etal. 1992; Morona etal. 2004; Kadioglu etal. 2008; Hyams etal. 2010). As the outermost layer of the bacterium, the capsule is the target of all licensed pneumococcal vaccines. However, the large diversity of capsular polysaccharides constitutes a major challenge for eliminating pneumococcal disease. The most commonly used conjugate vaccines currently target 10 or 13 of the most common capsular types (serotypes), but today almost 100 distinct serotypes have been described and recognized. Each serotype has a unique, experimentally confirmed serological profile (Henrichsen 1995), and for many of them the biochemical structure is known (Geno etal. 2015).

The polysaccharide capsule in the pneumococcus is synthesized by genes located in the *cps* locus (Munoz etal. 1997). Systematic genetic sequencing revealed that the diversity of *cps* alone forms a repertoire of almost 2,000 coding sequences (Bentley etal. 2006). These genes are divided based on their functions and form three major groups (Yother 2011; Geno etal. 2015). The first group is located upstream of the locus and consists of modulatory *wzg*, *wzh*, *wzd* and *wze* genes (aka. *cpsABCD*), which are common to almost all serotypes. The second group is serotype-specific genes (i.e., glycosyltrasferases and acetyltrasferases) with polymer-specific functions, and these define a serotype. Finally, many serotypes carry sugar-synthesis genes needed for capsule production (e.g., rhamnose genes). Comparison of the genetic content of different serotypes demonstrated that capsular gene acquisition and loss had been the underlying cause of emergence of many serotypes (Aanensen etal. 2007; Mavroidi etal. 2007). This is not surprising as the pneumococcus is known to undergo frequent recombination (Feil etal. 2000; Henriques-Normark etal. 2008; Vos and Didelot 2009), and the *cps* locus was shown to have elevated recombination rates in several lineages (Croucher etal. 2011; Chewapreecha etal. 2014). Furthermore, we know from previous studies that the extent of within-serotype diversity is under-appreciated, with many hybrid serotypes circulating in the population (Salter etal. 2012; van Tonder etal. 2016). However, the evolutionary dynamics, and hence the full adaptive potential of pneumococcal capsular polysaccharides, are not well understood.

The aim of this study was to gain a high-resolution view of the evolution of capsular polysaccharides in *S. pneumoniae*. In particular, we wanted to infer the rates of evolution and recombination within the *cps* locus, compare these parameters between different serogroups, and compare the relationship between evolution affecting capsular genes and that affecting the remainder of the genome. To this end, we analyzed capsular diversity in a collection of 4,469 pneumococcal isolates from several different studies, as well as 50 nonpneumococcal streptococcal isolates (see supplementary tables S1 and S2, Supplementary Material online). Our capsule-centered approach allowed us to observe the evolution of the pneumococcus from the point of view of the capsule itself, subdivided into major serotypes and serogroups, with the tree showing the evolution of the *cps* locus and the tips of the tree containing the information about changes between different genomic backgrounds. By disentangling horizontal from vertical genetic changes, we gained insight into the timescales of diversification and recombination in capsular genes. This approach brings novel qualitative and quantitative insight into the evolution of serotypes, the principal target of current vaccines.

## Results

### Species-Wide Serotype Diversity

**Fig. 1.**
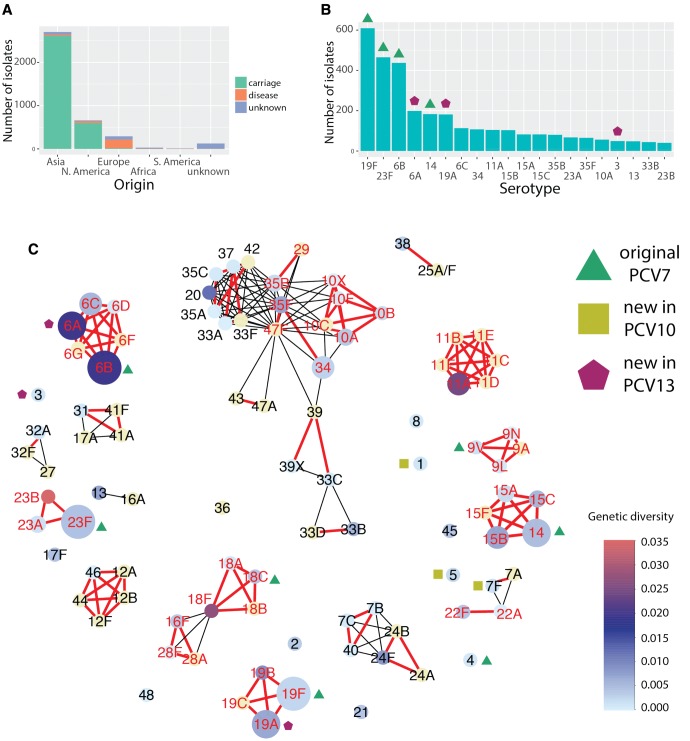
Properties of the dataset. (*A*) The distribution of the number of isolates stratified by geographical location and the source of isolation (carriage, disease or unknown). (*B*) The distribution of the number isolates stratified by the serotypes (top 20 shown). (*C*) A diversity network, where each node is represented by a *cps* reference sequence and an edge links two nodes if they are similar, i.e., share a minimum proportion *s* of the homologies. Red, bold edges show the conservative network where the minimum similarity was defined as sharing at least *s *=* *0.58 of homology groups (see Materials and Methods). Black edges show the additional connections obtained in a liberal network, where the minimum similarity was defined as sharing at least *s *=* *0.36 of homology groups. The size of each node reflects the full sample size, and the color shows the within-serotype *cps* genetic diversity measured using the mean pairwise Kimura K80 distance for all nonidentical isolates (full diversity distribution is shown in supplementary fig. S4, Supplementary Material online). Red labels of serotype nodes denote genetic serogroups which are analyzed in detail below.

To study the evolution of the *cps* locus, we analyzed several collections of pneumococcal isolates including two large carriage cohorts from Maela, Thailand (Chewapreecha etal. 2014) and Massachusetts (Croucher etal. 2013); three widespread lineages, PMEN1 (clonal complex CC81), PMEN2 (CC90) and PMEN14 (CC236) (Croucher etal. 2011; Croucher, Chewapreecha, etal. 2014; Croucher, Hanage, etal. 2014); *cps* reference collection (Bentley etal. 2006); Dutch isolates from invasive disease (Elberse etal. 2011); and publicly available reference genomes from the European Nucleotide Archive (ENA). This gave a total number of 4,469 isolates from 29 countries and 5 continents, as shown in figure 1*A*. To extract the *cps* locus in Illumina-sequenced isolates (96%), we reassembled them using a novel assembly pipeline (see Materials and Methods). In total, we obtained 3,813 full pneumococcal *cps* sequences, which were serotyped in silico (see supplementary table S3, Supplementary Material online). Figure 1*B* shows the observed serotype distribution, with 47% of the identified *cps* sequences being serotypes targeted by the seven-valent pneumococcal conjugate vaccine (PCV7) and 59% being serotypes targeted by the more recent 13-valent vaccine (PCV13). Altogether we identified 96 reference serotypes consisting of 254 homology groups (henceforth referred to as serotype-specific genes).

Among the 96 references, there were two *cps* variants which have not been previously confirmed as new serotypes. Putative serotype 10X was found in five isolates from Maela and originally classified as 10B, 10F or 33B. Its genetic structure suggests that it is a mosaic of 10C or 10F with another serotype, possibly 35B (supplementary fig. S1, Supplementary Material online). The same variant was termed 33X in a recent report by van Tonder etal. (2016), however, genetic analysis shows that it shares more serotype-specific genes with serogroup 10 than 33, and in this study, we analyzed it together with the remaining members of serogroup 10. Putative serotype 39X was found in three isolates from Maela (see Supplementary Material online), and to our knowledge has not been previously described. Its genetic structure suggests that it could have arisen as a recombination between 6C/6D and 39 (supplementary fig. S2, Supplementary Material online). Indeed, in a serotyping experiment 39X reacted to antiserum which covers serotypes 33 and 39. From this, we can conclude that pneumococci with the 39X *cps* locus are capable of producing a novel capsular polysaccharide, and that it may have some cross-reactivity to existing pneumococcal sera (see Supplementary Material online).

To visualize *cps* diversity within the pneumococcus, we generated a network with nodes represented by *cps* reference sequences and edges linking serotypes of minimum similarity *s*, defined as a maximum of the two proportions of shared serotype-specific homology groups between each pair (see Materials and Methods). Figure 1*C* shows this similarity network for two different thresholds, conservative (*s *=* *58%) and liberal (*s *=* *36%), with two corresponding edge types depicted in the figure. We see that serotype clusters in the conservative network (henceforth referred to as genetic serogroups) are often congruent with phenotypic serogroups. Nevertheless, there are exceptions: e.g., serotype 16F clusters with serogroup 28 with conservative threshold, but does not cluster with 16A even with liberal threshold. These observations are consistent with earlier findings and highlight the complexity of the polysaccharide genotype–phenotype map (Aanensen etal. 2007; Mavroidi etal. 2007). This approach also allows identification of mosaic triplets (nodes connecting other groups), which denote potential introgressive descents (Bapteste etal. 2012). In this way, one can quickly identify some of the mosaic *cps* variants, e.g., 18F (shares *wcxM* gene with 18A/18B/18C and 28A/28F/16F) or 22A (shares *wcwC* gene with 22F and 7A/7F). The largest connected component, which includes serogroups 10, 33, 34, 35 and others, is the one with the largest number of mosaic triplets. Indeed, a gene-sharing network analysis suggested that its members consist of the most interconnected serotypes (see supplementary fig. S3, Supplementary Material online). Therefore, we can conclude that many serotypes are highly mosaic in nature and their evolution was likely driven by horizontal transfer of DNA.

### Recombination Drives Emergence of Serotypes

We next investigated the diversification of different serogroups into serotypes. Gaining such insight from whole-genomes in *S. pneumoniae* is problematic. Due to frequent serotype switching, whole-genome based phylogenies for a serogroup in question would need to be constructed based on distant bacterial lineages. However, previous studies have shown that high recombination rates in the pneumococcus can obliterate the phylogenetic signal at deeper branches (Feil etal. 2001; Mostowy etal. 2017). This suggests that whole-genome trees based on distant pneumococcal lineages could not be used to reliably infer the underlying evolutionary processes leading to the diversification of serogroups. To circumvent this problem, here we obtained clonal trees based on *cps* sequences of genetic serogroups defined by red edges in figure 1*C* (nodes marked with red labels). This approach has two main advantages. First, by focusing on closely related serotypes which share a large majority of their tightly linked genetic content, we can more reliably apply standard population-genetic tools and infer the underlying clonal phylogeny. Second, this approach allows to view the evolution of the pneumococcus from the perspective of the capsule itself, with the tree showing the evolution of the *cps* locus and the tips of the tree containing the information about how the capsule changes between different genomic backgrounds.

**Fig. 2.**
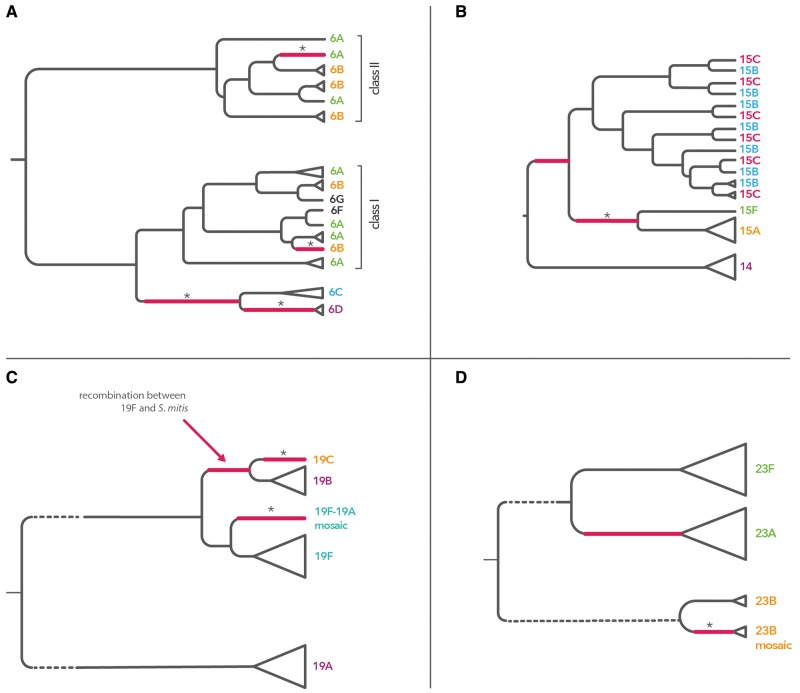
Evolution of the four most common serogroups. Schematic dendograms shows the evolutionary history of the four major serogroups, which correspond to four largest clusters marked in red in figure 1: serogroup 6 (panel *A*), serogroup 14/15 (panel *B*), serogroup 19 (panel *C*), and serogroup 23 (panel *D*). The dendograms are based on the clonal trees inferred using *cps*-based alignments, one analyzed for each serogroups. Full resolution figures can be found in Supplementary Material online. Recombinations which occurred on branches leading to a new serotype or a mosaic are colored in red. The star sign is marking those branches where there was statistical support for the recombination (using STRUCTURE or Gubbins), and the remaining ones were hypothesized to have occurred based on the gene content comparison (see detailed discussion in Supplementary Material online). Clonal uncertainty due to the suggested model is reflected by dashed branches. The origin of the detected recombinations is analyzed in figure 5.

Twelve most numerous and diverse serogroups were analyzed one by one, here referred to as serogroups: 6, 19, 23, 14/15, 18, 10, 11, 9, 34/35, 16/28, 29/35 and 22 (see Materials and Methods and supplementary fig. S4, Supplementary Material online, for serotype diversity comparison). In addition, we included here two nonpneumococcal sequences, one closely related to serotype 19C (*S. mitis* SK564; see supplementary table S2, Supplementary Material online) and the other one closely related to serotype 18F (*S. mitis* SK667). In brief, sequences from each serogroup were aligned and the population genetic structure was analyzed, with recombinations within each sub-population identified using two different methods (Falush etal. 2003; Croucher, Page, etal. 2015). The recombinant fragments were then removed from the alignment and the resulting clonal alignment was used to construct a tree of the serogroup, with recombinations mapped onto the tree. Figure 2 summarizes the inferred model of evolution for the four most common serogroups, 6, 19, 23 and 14/15, while supplementary figure S5, Supplementary Material online, shows dendograms of the remaining eight serogroups. The details of the analysis for all serogroups are given in Supplementary Material online and supplementary figures S6–S29. Here, we briefly summarize the main findings.

First, the critical event in the emergence of at least seven serotypes was a recombination importing extensive genetic diversity, as indicated by the causative change being associated with a cluster of polymorphisms on the ancestral branches of these serotypes. In four of these cases, the source could be identified (see table 1 and Supplementary Material online). Second, in serogroup 19 we hypothesize that the 19B/19C clade arose by recombination of 19F with *S. mitis*, and in serogroup 23 that serotype 23A emerged by recombination of 23F and a capsule of an unknown source (see Supplementary Material online and fig. 2). Third, we detected many recombinations which did not change serotype but sometimes produced mosaic isolates, for 6B-I/6B-II mosaic (supplementary fig. S6, Supplementary Material online), 19A/19F mosaic (supplementary fig. S8, Supplementary Material online), or 23B-mosaic (supplementary fig. S10, Supplementary Material online). Fourth, population genetic structure of the common *wz*-genes shows presence of many older, undetected recombinations (see supplementary fig. S30, Supplementary Material online). Finally, in many cases, we observed that a simple model of gene gain and loss cannot explain the observed patterns of diversity. In particular, we found serotypes 6A/6B, 15B/15C and 18B/18C to have emerged on multiple independent occasions. In the case of serogroup 6, this emergence was due to recombination (see fig. 2 and supplementary fig. S7, Supplementary Material online), in the case of serogroup 18 it was due to a point mutation (see supplementary fig. S15, Supplementary Material online) and in the case of serogroup 15 it was due to frameshift mutations with switches possibly affected by homologous recombinations (see fig. 2 and supplementary fig. S13, Supplementary Material online). Hence the evolutionary history of these serotypes is a complex story of repeated recombinations of differing phenotypic consequences.

**Table 1.** Direct Evidence for Emergence of Serotypes by Recombination.

Serogroup	Serotype(s)	Gene(s) Affected	Likely Source
6	6C	*wciN*	Unknown (homolog in 39X)
6	6D	*wciP, wzy, wzx*	6B-II
19	19B/19C	*wchU*	*Streptococcus mitis*
10	10C	*wcrW*	10A
34/35	47F	*whaI*	47A
16/28	28A	*wciU*	Unknown
22	22A/22F	*wcwC*	Unknown

Note.—The table summarizes the cases in recombinations directly detected in our approach were associated with the emergence of new serotypes. In some cases, the direction of recombination could not be established; in this case, multiple serotypes were given in the second column. Details of the analysis are given in Supplementary Material online.

### Molecular Clock of the Capsule

**Fig. 3.**
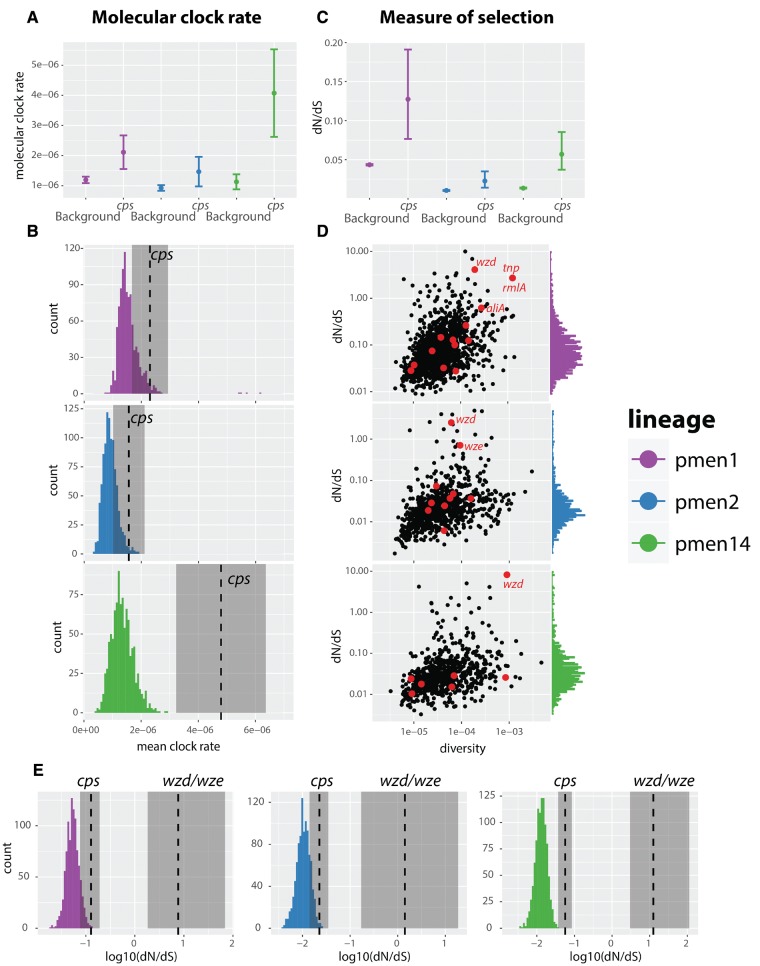
The molecular clock rate and selection in the *cps* locus in three different lineages, PMEN1, PMEN2 and PMEN14. (*A*) The inferred molecular clock rate of the whole-genome alignment as inferred by BEAST2, with the capsule removed (background), and to the capsule-only alignment (*cps*), with error bars showing 95% highest posterior density. (*B*) The null distribution of the molecular clock rate in the genome, measured in 20 random regions from the genome (repeated 1,000 times), versus the clock rate of the *cps* locus. (*C*) Comparison of the mean ω = *dN*/*dS* value in the genome versus the *cps* locus. (*D*) Distribution of ω values estimated for each coding sequence versus underlying gene diversity measured using K80 model. The values of ω lying outside the 95% quantile range are not shown. (*E*) Null distribution of ω values in the background is compared with the estimated ω in the *cps* and the *wzd/wze* region. Shaded regions show the 95% confidence intervals.

We next wanted to learn about the timescales of the evolutionary process within the *cps*. Analysis with TempEst (previously Path-O-Gen; Rambaut etal. 2016) revealed that the serogroup alignments did not have enough temporal signal to robustly infer the substitution rate of the *cps* locus. Therefore, we used whole-genome collections of three globally disseminated lineages PMEN1 (Spain^23*F*^-ST81), PMEN2 (Spain^6*B*^-ST90) and PMEN14 (Taiwan^19*F*^-ST236). The wide sampling time range of these lineages allows the estimation of the molecular clock rate, as was supported by a significant correlation between the sampling date and the root-to-tip distance in all three alignments (Croucher etal. 2011; Croucher, Chewapreecha, etal. 2014; Croucher, Hanage, etal. 2014). To obtain the molecular clock rate, for each lineage, we simultaneously fitted two separate clock models, one to the entire alignment with the *cps* removed, and the other one to the *cps* only, defined by coordinates of *dexB* and *aliA* genes (see Materials and Methods), all using BEAST2 (Bouckaert etal. 2014). Results are displayed in figure 3*A* and show that on an average we observed a roughly 2.5 times higher clock rate in the *cps* locus compared with the rest of the genome. The distribution of SNPs across capsular genes (see supplementary fig. S31, Supplementary Material online) suggests no bias at transposable elements (their repetitive nature can sometimes produce such bias due to false-positive substitutions). We can thus conclude that the observed substitution rate is not an artifact of data assembly.

The estimated difference in clock rate of *cps* compared with the background is calculated assuming that the latter is homogeneous across the genome. This is obviously false as not all proteins evolve at the same rate. We thus next investigated how the molecular clock rate of *cps* compares to the expected range of clock rates in different areas of the genome. To this end, we randomly sampled 1,000 times a genetic region from the genome of roughly the same length as the *cps* locus, and used BEAST2 to estimate the clock rate in that region. We then compared the distribution of clock rates to the one in the *cps* (see Materials and Methods). The comparison (see fig. 3*B*) shows that *cps* is consistently a rapidly evolving region in the genome, and in PMEN14 it is significantly higher than given by the null distribution.

To investigate whether the increased substitution rate within *cps* is due to varying rates of selection acting on different proteins, we estimated ω = *dN*/*dS* in different coding regions of the three PMEN lineages (see Materials and Methods). The comparison of the average ω value in the genome versus in the *cps* (fig. 3*C*) shows that the capsule has an elevated proportion of nonsynonymous substitutions compared with the rest of the genome. We then compared the value of ω between different genes (fig. 3*D*). Interestingly, we found that the distribution of ω values across capsular genes largely overlaps with the distribution of ω values across the genome, with the exception of a few genes with an unusually high ω. In all three lineages, these included the *wzd* gene, and in the case of PMEN2 also the *wze* gene. As both *wzd* and *wze* genes have previously been hypothesized to play a role during colonization of the host, and as both of them showed a trend of elevated diversity compared with other *cps* genes, we next tested the hypothesis that these genes have been under diversifying selection. To this end, we estimated ω as well as the number of synonymous and nonsynonymous substitutions for the *wzd/wze* region (see table 2). In both PMEN1 and PMEN14, we found the *wzd/wze* region to contain a significantly greater proportion of nonsynonymous to synonymous substitutions compared with other genes in the *cps* locus. Comparison of ω in the *wzd/wze* region with the one estimated for the entire *cps* locus and a background-derived null distribution is presented in figure 3*E*. It shows that the slightly higher value of ω in the *cps* is likely driven by the high value of ω in the *wzd/wze* region. Thus, we conclude that the higher level of selection in the *cps* locus is likely driven by strong, positive, diversifying selection acting on the upstream *wzd*/*wze* genes.

**Table 2.** Synonymous versus Nonsynonymous Substitutions in *wzd* and *wze*.

Dataset	N in *wzd/wze*	S in *wzd/wze*	N in Other	S in Other	*P* value
PMEN1	21	1	39	15	0.03
PMEN2	9	1	19	6	0.64
PMEN14	37	1	7	3	0.03
Total	67	3	65	24	10^−4^

Note.—The table shows the number of synonymous (S) and nonsynonymous (N) substitutions in *wzd* and *wze* versus other genes in the *cps* locus in three PMEN lineages. *P* value shows the significance of the Fisher’s exact test examining the proportion of nonsynonymous to synonymous mutations in the *wzd/wze* region.

### Variability in Recombination Rates between Serogroups

**Fig. 4.**
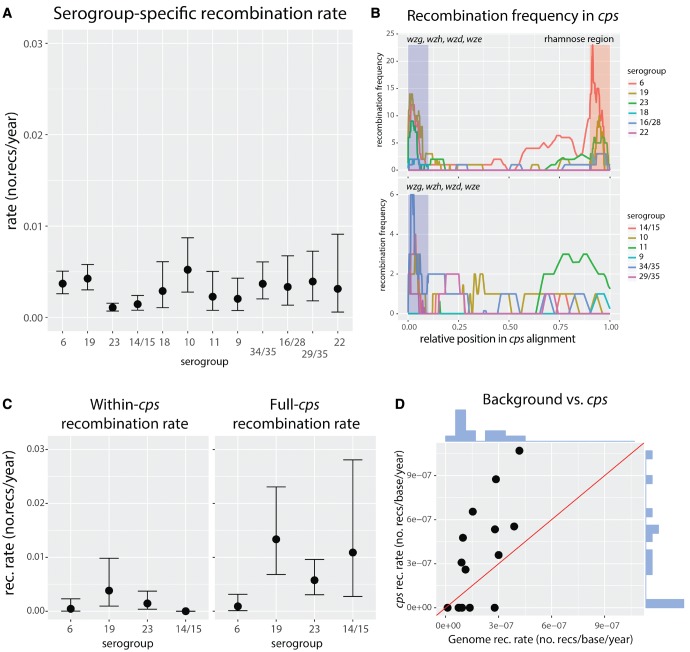
Recombination rates within the *cps* locus. (*A*) Recombination rates estimated for the 12 serogroups used in this study, with the relevant 95% confidence intervals. By definition these rates do not include long, *cps*-spanning recombinations which are invisible from the point of view of *cps* alignment. (*B*) Frequency of recombinations observed at the *cps* locus, with different colors showing number of recombinations for different serogroups, with rhamnose genes (top) and without rhamnose genes (bottom). Recombination positions were normalized such that total alignment length was 1 in all serogroups. Additionally, for the sake of comparison, the upstream *wg*-region (blue) and the rhamnose region (red) were normalized to 10% of the length each. (*C*) Recombination frequency measured using whole-genome approach, with within-*cps* events (left) versus full-*cps* events (right). (*D*) Recombination rate at the genomic background (excluding events at the *cps*) versus recombination rate of events affecting the *cps*, estimated from whole-genome alignments. Lineages of predominantly the same serogroup (80%) were chosen. The rates were normalized per base using mean alignment lengths of whole-genome and *cps*, respectively. The *y *=* x* line is shown in red, and marginal distributions are shown in blue.

Using the *cps* clock rate estimated for PMEN collections and the sampling dates obtained for the isolates used in this study, we next estimated the branch lengths of the maximum likelihood (ML) trees for each of the serogroups. This also allowed us to infer the divergence times of serogroups (four largest are shown in supplementary figs. S32–S35, Supplementary Material online) with the times of occurrence of corresponding recombination events, as well as to estimate the recombination rate for each serogroup in more intuitive units. Figure 4*A* shows the comparison of the obtained recombination rate estimates for the 12 examined serogroups. The results demonstrate a significant heterogeneity in recombination rates across all serogroups (Fisher’s exact text, number of tree branches with recombinations vs. number of tree branches without recombinations per serogroup, *P* = 3×10^−5^). To test whether sampling can explain the observed variance in recombination rates, we examined associations between the rates and five measures of sampling: number of isolates, number of nonidentical isolates, genetic diversity, number of countries in which each serogroup was sampled and lineage diversity (see supplementary table S4, Supplementary Material online, and Materials and Methods). We did not find a significant association between the recombination rate and any of the quantities (Spearman rank test, *P *>* *0.05 in all cases). As this corresponds to theoretical expectations (see supplementary fig. S36, Supplementary Material online), we conclude that sampling cannot explain the observed variation in recombination rates.

One potential determinant of the observed variation in recombination rates is the variation in background recombination rates, which are known to vary between pneumococcal lineages (Croucher etal. 2013). However, the recombination rates in figure 4*A* were inferred using serogroup alignments, and are thus likely to miss long recombinations (with breakpoints outside the *cps*) which drive serotype switching. One can expect that such shorter, within-*cps* recombinations would be observed less frequently for two reasons. First, the recombination rate at serotype-specific genes may be lower when recombination occurs between different serotypes or serogroups due to the lack of sequence similarity at the flanks of the transformed segment. Second, recombinations which are mechanistically possible could be under natural, negative selection as they are more likely to disrupt the polysaccharide structure, thus affecting the viability of the capsule. These two hypotheses are consistent with the recombination frequency pattern observed in *cps* (see fig. 4*B*). However, the absolute and relative impact of the two processes could vary between different serogroups, e.g., due to varying epistatic interactions of capsular genes or co-colonization rates of different serogroups. In such case, the ratio of within-*cps* recombination rate to full-*cps* recombination rate could vary between serogroups, thereby affecting the variation observed in figure 4*A*.

To measure the ratio of within- to full-*cps* recombinations, we next compared the frequency of all recombinations affecting the *cps* locus (full-*cps* recombination rate) with the frequency of recombinations contained within the *cps* (within-*cps* recombination rate). To detect both types of recombinations, we used a whole-genome lineage-by-lineage approach. We focused on the four major and most frequent serogroups (6, 19, 23, 14/15), and to minimize the potential impact of missing data due to mapping we picked only those lineages in which at least 80% isolates were of the same serotype. We then estimated the two recombination rates for each serogroup (see Materials and Methods and supplementary table S5, Supplementary Material online, for the list of all lineages). Results are displayed in figure 4*C* and show the comparison of the mean within-*cps* recombination rate (left) and the mean full-*cps* recombination rate (right). As expected, we generally observed higher full-*cps* rates compared with within-*cps* rates. Comparison of the recombination rates revealed different within-full recombination rate ratios (serogroup 6: 0.500, 95% CIs 0.02 − 0.98; serogroup 19: 0.286, 95% CIs 0.066 − 0.586; serogroup 23: 0.250, 95% CIs 0.050 − 0.530; serogroup 14/15: 0, 95% CIs 0 − 0.470). Given the significant heterogeneity between these ratios (one-way ANOVA; *P* < 10^−10^), these results suggest that impact of ecological and genetic factors affecting the observed within-*cps* recombination rate could vary between different serogroups, which could contribute to the variation observed in figure 4*C*.

Having established that the within-*cps* recombination rate is only partly predictive of the full-*cps* recombination rate, we next investigated how well the background recombination rate predicts the full-*cps* recombination rate (fig. 4*D*). We found that the former explains roughly half of the variance in recombination rates between serotypes (linear regression, *R*^2^=0.47, *P *=* *0.005). Finally, we investigated whether the measured recombination rates could be explained by capsule thickness. As capsules are known to constitute a physical barrier to incoming transformation events (Schaffner etal. 2014), we hypothesized that isolates with thicker capsules may exhibit lower rates of recombination. To test this, we obtained the measurements of the zone of exclusion of fluorescent dextran molecules by the capsule from Weinberger etal. (2009), which provided estimates of the degree of encapsulation. We found a positive, although nonsignificant, relation between the mean serogroup capsule size and the estimated recombination rate (Spearman rank test, *P *=* *0.4; see supplementary fig. S37, Supplementary Material online). A positive relationship between the two quantities would be in line with previous findings relating larger capsule size with increasing rates of per-lineage recombination in pneumococci (Chaguza etal. 2016). Such a relation could stem from a correlation between thicker capsules and increased duration of carriage, and the fact that serotypes which are carried longer tend to recombine more frequently. However, the nature of such a relation will require further investigation.

### Origin of Capsular Recombinations

**Fig. 5.**
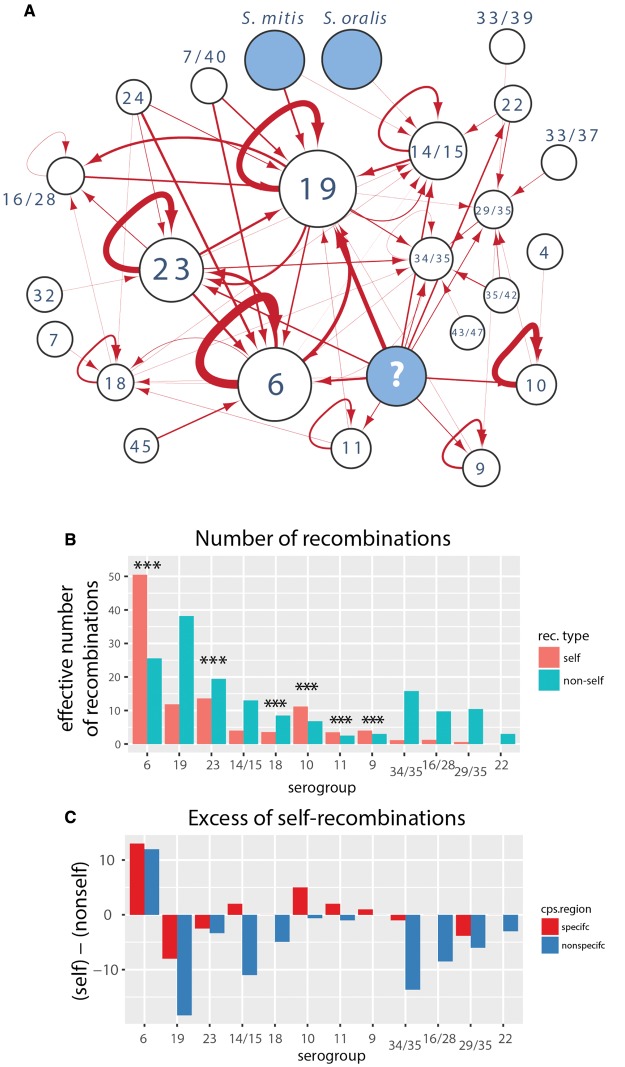
Origin of *cps* recombinations. (*A*) The network shows the recombination flow among the serogroups defined in figure 1*A*: nodes correspond to serogroups and arrows correspond to the direction of *cps* recombination flow based on the most likely origin of the putative recombination events. The width of arrows reflects the number of recombination events (between 0 and 51) and the size of the nodes reflects the number of isolates within the serogroup (except for “unknown” and other streptococci). (*B*) Proportion of *cps* recombinations originating in the same serogroup (self) versus another serogroup (nonself) for each serogroup. Stars show significance of the departure from random distribution of recombination exchanges. The significance was calculated assuming a binomial distribution of self-/nonself-recombination with the probability corresponding to the frequency of self-/nonself-serogroups. (*C*) Excess of self- over nonself-recombinations in the *cps*-specific and *cps*-nonspecific region, as defined in the main text.

We next investigated the origin of the recombination events identified in the *cps* locus. To this end, we used BLAST to identify close hits (defined by minimum 90% identity; see Materials and Methods) with multiple hits assigned a proportionally lower weight; otherwise the origin was considered unknown. We also included a set of 50 *S. mitis* sequences mentioned in supplementary table S2, Supplementary Material online. Figure 5*A* shows a recombination flow diagram, namely a directed network with nodes as serogroups and directed edges (arrows) indicating the direction of recombination between different serogroups. We identified potential source for 91% of recombinations. It is unclear whether the remaining recombinations descended from the same or other bacterial species. However, we would not expect to find many cases of inter-species recombinations for several different reasons, including biological ones (stronger purifying selection of more diverse imports) and methodological ones (underrepresented diversity of nonpneumococcal streptococci in genomic datasets). Nevertheless, in the case of serogroup 19, we found four recombinations with close homology to *S. mitis*, and in serotype 14 one recombination with close homology to *S. oralis*. Overall, we also found that more recombinations originated in other serogroups compared with the same serogroup (156 vs. 105). This proportion is inevitably affected by the detection bias in that recombinations bringing more substitutions are more likely to be detected than imports of closely related nucleotide sequences. However, since such bias is not expected to depend on the serogroup, we next quantified the number of recombinations originating in the same (“self”) versus in different (“nonself”) serogroups (fig. 5*B*). We found that most serogroups have more nonself-recombinations, but in three serogroups (6, 10 and 11) the majority of recombinations originated in the same serogroup. In fact, for six serogroups (6, 23, 18, 10, 11, 9) we found a significant departure from randomness in the distribution of recombinations (with distributions modeled as binomial with probabilities conditional on the observed serogroup frequencies; see Materials and Methods), suggesting that at least for some serogroups basic frequency distribution cannot explain the observed self-/nonself-recombination ratios. This points to unexpected patterns of co-occurrence of different serogroups, which could be driven by competition during host colonization (see Discussion). Finally, the analysis of excess of self-recombinations over nonself-recombinations in different regions of the *cps* (fig. 5*C*) shows that in some serogroups the pattern of self-/nonself-proportion holds for both serotype-specific genes (which are rare in other serogroups) and in capsule-nonspecific genes (which are common in other serogroups).

### Capsule Lineage Jumping

**Fig. 6.**
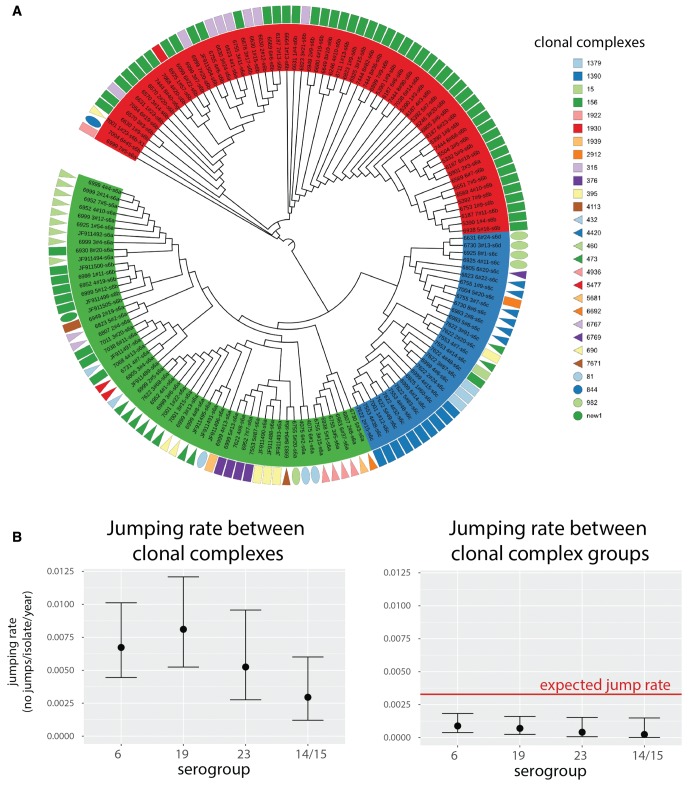
Lineage-jumping dynamics. (*A*) Dendogram based on the phylogeny of serogroup 6. Tips are grouped according to the three major populations identified: class-I 6A clade (green), 6C/6D clade (blue) and class-II 6B clade (red). Geometric shapes aligned with the tips denote the corresponding clonal complexes of the strains in which the serotype sequence was found. The clades of the tree with branch lengths shorter than 9 × 10^−5^ were collapsed and the most frequent lineage within the clade was plotted. (*B*) Lineage-jumping rates inferred for the four most frequent serogroups (6, 19, 23 and 14/15) with lineages defined as clonal complexes (CC; left) and as clonal complex groups (CCG; right). The red line shows the CCG jumping rate expected based on the observed CC jumping rate and the assumption that changes between all pairs of CC are equally likely. Mean estimate and the 95% of the highest posterior density is shown.

Analysis of *cps* sequences as performed here allows the evolution of the species to be observed from the point of view of the antigen which occasionally alters its genetic background via “lineage jumping.” Such jumps should be observed as alterations of clonal complexes (lineages) within individual clades of serotype trees. The example of serogroup 6 (fig. 6*A*) demonstrates a large within-clade variation of clonal complexes. To estimate the rates of lineage-jumping for four major serogroups (6, 19, 23 and 14/15), we defined a clonal complex (CC) using eBurst (Feil etal. 2004) by linking isolates with 6/7 MLST-locus identity, and a clonal complex group (CCG) by linking isolates with 5/7 MLST-locus identity. We next used BEAST2 to predict the rate with which an isolate in each serogroup is expected to jump lineage (see Materials and Methods). The results are shown in figure 6*B*. We found the mean jumping-rate between CCs to be 5.8 × 10^−3^ jumps per isolate per year, and between CCGs to be 5.6 × 10^−4^ jumps per isolate per year. If changes between all pairs of CCs were equally likely, we would expect that 57% of them would alter the CCG. Thus, under a random CC-jump model, we would expect the CCG jumping rate to be roughly 0.57 times the CC jumping rate. Instead, we found the CCG jumping rate to be lower than expected. These results suggest that pneumococcal serotypes are less likely to jump lineages if those lineages are very distant. This is consistent with the observation that most pneumococcal serotype switches were previously found to occur within a serogroup (Croucher, Kagedan, etal. 2015).

## Discussion

The pneumococcal capsule biosynthesis locus, *cps*, is an evolutionary hotspot, presumably underlying immune selective pressures acting on this major antigen. In the *cps*, we found elevated molecular clock rate and elevated recombination rate compared with the rest of the genome. The elevated clock rate can be explained by two selective forces acting on the capsule: relaxed purifying selection and diversifying selection. The former is consistent with previous observations that nonessential bacterial proteins are expected to evolve faster than essential proteins (Jordan etal. 2002), and that a viable capsule is not essential for evolutionary success as nonencapsulated pneumococci have been found both in carriage and disease (Chewapreecha etal. 2014; Dixit etal. 2016). Using three, globally disseminated lineages, we also found evidence for diversifying selection acting on the region of *wzd/wze* genes, which are located within the *cps* locus upstream of the serotype-specific genes. These two genes have been previously demonstrated to play a role in the interaction with the host; previous studies found mutations in *wzd* to affect attachment to the cell wall (Morona etal. 2006), and mutations in *wze* to affect capsule biosynthesis (Morona etal. 2004), both essential for successful colonization as well as ability to penetrate into blood and cause invasive disease. As different alleles of the two genes have been associated with serotype’s invasiveness (Varvio etal. 2009), it is thus conceivable that isolates are under constant selective pressure to alter capsule expression due to varying epidemiological forces or within-host factors.

We also found recombination rate at the *cps* locus to vary substantially between lineages (see fig. 4*D* and supplementary fig. S38, Supplementary Material online). The average rate of recombinations affecting the *cps* was ∼2.5 times higher than the genome-wide recombination rate (see supplementary fig. S39, Supplementary Material online), and the latter was generally a good predictor of the former. Why does capsule recombine so frequently? One explanation could be that this is a consequence of increased recombination-detection power due to increased diversity. However, homologous recombination preferentially occurs between closely related isolates (Majewski etal. 2000; Majewski 2001; Ansari and Didelot 2014), and most recombinations in *cps* between two random isolates are not going to be possible due the lack of homology between most capsular genes. Furthermore, computer simulations have shown that most recombinations occurring on such evolutionary timescales are detectable (Mostowy etal. 2014; Croucher, Page, etal. 2015). Thus, the recombination rate is more likely elevated for the same reasons as the molecular clock, namely that most of the observed recombinations are either those which selection has not yet had time to eliminate, or those promoted by diversifying selection. This hypothesis is in line with the observation that in densely sampled areas there is a higher chance of finding a rare recombination which has not yet been purged: the densely sampled Thai collection featured two mosaic *cps* sequences which have not been observed outside Maela, while the average recombination rate in the Thai collection was found to be higher compared with the Massachusetts collection (see supplementary fig. S39, Supplementary Material online). Furthermore, the two genes in which evidence for diversifying selection was found here often serve as recombination breakpoints during serotype switching (Croucher, Kagedan, etal. 2015). Given that these genes also harbor older recombinations which are difficult to detect by other methods (see supplementary fig. S30, Supplementary Material online), it is conceivable that the selective pressure driving the diversification of *wzd*/*wze* could also promote recombination events in the *cps* locus, thus driving the observed recombination rate. Conversely, it could be selection for diversity acting on the neighboring serotype-specific genes driving the elevated recombination rate, which in turn inflates the diversity observed in the regulatory *wzd/wze* genes. Either way, it seems that selection produces recombination hotspots in the capsule, and this translates into an increased capsular diversity over time.

Interestingly, variation in serogroup-specific recombination rates cannot be fully explained by the differences in the background recombination rate. Our results point to an absolute as well as relative impact of two additional processes: 1) Decreased frequency of homologous recombinations at the *cps* locus between distant serotypes due to selection for sequence similarity and the flanks and 2) negative selection against recombinations which disrupt the polysaccharide structure. In addition, we found unexpected patterns of recombinational exchanges in *cps*: in several serogroups, we found many more recombinations originating in the same serogroup than expected from the frequency pattern alone. Even expecting some donor-detection bias in favor of more common serogroups, we found significant departure from randomness in both frequent and rare serogroups as well as no departure from randomness in both frequent and rare serogroups. Thus, the observed patterns of exchanges likely reflect variation in co-occurrence rates between different serotypes and serogroups. Competitive interactions between serotypes have been shown to exist in mice invivo (Trzcinski etal. 2015), and this indeed suggests that such co-occurrence rates may not linearly depend on the serotype frequency distribution. In summary, the observed recombination rates in different serogroups are a result of the genetic, microbiological, ecological, epidemiological and evolutionary processes acting on the pneumococcal capsule.

Our results suggest that inter-species recombination plays an important role in the evolution of the *cps* locus. One prominent example is the emergence of the 19B/19C clade by recombination of 19F with *Streptococcus mitis*. Furthermore, five detected recombinations bore close resemblance to *S. mitis* and *S. oralis* isolates. In addition, many serotype-specific genes are shared between different streptococci (Kilian etal. 2014; Sørensen etal. 2016). Given the scale of genetic diversity across the mitis group streptococci (Kilian etal. 2008), this suggests that many older, hypothesized recombinations within the *cps* locus (e.g., the emergence of 23A by acquiring *wzy* gene) were probably acquired from other closely related bacteria. However, the timescales of such processes remain unclear and require a better characterization of the polysaccharide diversity in all nasopharyngeal bacteria.

The novel, capsule-centered approach devised here provides a perspective on the evolution of the bacterium from the point of view of its major antigen. This has two main advantages. First, it permits reconstruction of the evolutionary history of different serogroups by combining genetic data from distant lineages. Second, changes in the genomic background on the serogroup-based tree can inform us about the evolutionary dynamics of the serotype switching process. Nevertheless, the main limitation of this approach was the difficulty detecting long recombinations with breakpoints outside the *cps* locus. This was enhanced by the fact that we excluded external IS elements and *dexB/aliA/aliB* genes, all of which flank the serotype-specific gene cluster. However, since we found comparable recombination rates for the four major genetic serogroups in both *cps*-based approach and the lineage-by-lineage approach, it suggests that these limitations are unlikely to affect the key findings of this study.

All evidence thus points at the *cps* locus being a genetically plastic and dynamic locus under diversifying selection, with recombination being its main evolutionary driver. While most recombinations are expected to either be under weak negative selection or produce nonviable capsules (Park etal. 2014), occasionally mosaic, previously unseen capsules can emerge. Indeed, the densely sampled Thai collection contained two mosaic serotypes, here termed 10X and 39X (the latter was tested and confirmed to carry a viable and structurally novel capsule), while a 33B/33C hybrid was previously identified in an isolate from Denmark (Salter etal. 2012). Given 1) that dense sampling leads to the discovery of more recombinations, 2) that identifying a novel, mosaic capsule requires a detailed, comparative approach, and 3) the enormous diversity of glycosyltransferases and acetyltransferases in the microbial world, we can expect that many more such hybrids are circulating around the world. Why are these hybrids not spreading in the population? This could be due to several different factors, including cross-immunity (Lipsitch 1997), competitive exclusion (Trzcinski etal. 2015) or fitness differences (Cobey and Lipsitch 2012). However, introduction of broader, conjugate vaccines in the future may empty ecological niches occupied by the common serotypes, and provide a selective advantage for some of the rare, mosaic serotypes, which could increase in frequency over time. Therefore, a systematic characterization of *cps* diversity across different nasopharyngeal species is important for a better characterization of the true pneumococcal adaptive potential.

## Materials and Methods

### Isolates

We combined several, previously published genetic and genomic data collections of *S. pneumoniae*. These include 3,085 isolates from a continuous mother–infant carriage study in the Maela refugee camp, Thailand (Turner etal. 2012; Chewapreecha etal. 2014), 616 isolates from children carriage in Massachusetts (Croucher etal. 2013), and 605 globally disseminated isolates from three lineages defined by the Pneumococcal Molecular Epidemiology Network: PMEN1 or CC81 (Croucher etal. 2011), PMEN2 or CC90 (Croucher, Hanage, etal. 2014), and PMEN14 or CC236 (Croucher, Chewapreecha, etal. 2014). We also included 45 sequences of serogroup 6 and 19 isolates from invasive disease from the Netherlands (Elberse etal. 2011), 92 reference sequences (Bentley etal. 2006; Park etal. 2007; Bratcher etal. 2011; Oliver etal. 2013), and a set of 25 reference genomes of *S. pneumoniae* as found in the European Nucleotide Archive www.ebi.ac.uk/genomes/bacteria.html. The total 4,469 isolates are listed in supplementary table S1, Supplementary Material online, while 50 publicly available isolates of (closely related) *S. mitis*, *S. oralis* and *S. pseudopneumoniae* are listed in supplementary table S2, Supplementary Material online.

### Assembly of Whole-Genome Lineages

Two largest collections used in this study (Massachusetts and Thailand) were assembled and analyzed as described in the original publications, with assemblies and the corresponding assembly statistics publicly available (Chewapreecha etal. 2014; Croucher, Finkelstein, etal. 2015). Briefly, core-genome alignments were constructed and their population structure was analyzed by BAPS (Corander and Marttinen 2006). The resulting 15 largest monophyletic clusters in the Massachusetts collection and seven largest monophyletic clusters in the Thai collection were chosen for the lineage-by-lineage analysis. The corresponding isolates were mapped to closely related references to produce whole-genome alignments. In addition, whole-genome alignments of the three PMEN lineages were obtained by mapping to the closest reference as described in the original publications (Croucher etal. 2011; Croucher, Chewapreecha, etal. 2014; Croucher, Hanage, etal. 2014). We inferred the maximum likelihood (ML) clonal frame of these 25 alignments using Gubbins (Croucher, Page, etal. 2015), with recombinations mapped to nodes of the clonal tree. Imports occurring in regions annotated as mobile genetic elements were excluded. The list of all lineages with basic summary statistics, including serotypes and predominant sequence types for each lineage, is given in supplementary table S5, Supplementary Material online.

### Assembly of *Cps* Variants

As default assemblies were optimized for the construction of the whole-genome alignments, not all isolates had the *cps* locus contained in a single contig. To maximize the number of isolates for which full-length *cps* sequences were available, the assembly pipeline was reoptimized in the following way. All Illumina-sequenced isolates were reassembled using velvet (Zerbino and Birney 2008) with varied k-mer length (between 50% and 90% length of the short-read) and the expected coverage (between 5 and 140). The aim was to find an assembly which spanned as much of the entire *cps* locus as possible (defined by aligning the assembled and reference sequences using BLASTN with e-value <10^−50^) in as few contigs as possible; if multiple such assemblies were produced, the one with the least number of N’s and the highest *n*_50_ value was chosen. Due to the repetitive nature of insertion-sequence elements, sequences were assembled excluding *dexB/aliA/aliB* genes and transposable elements flanking the serotype-specific region, thus usually starting with *wzg* and ending with *rmlD*, *glf* or *wcjE*. (The list of all genes used as markers for the start and end of the serotype sequence in question is given in supplementary table S6, Supplementary Material online.) The resulting set of contigs was then analyzed for potential misassemblies using reapr (Hunt etal. 2013) and had gaps filled using GapFiller (Nadalin etal. 2012). Finally, isolates with the full *cps* locus were scaffolded against the corresponding reference sequence using ABACASS (Assefa etal. 2009) and GapFiller. Finally, the quality of each *cps* sequence was assessed visually by comparing to the reference, in search of false-positive polymorphisms associated with N’s in the assembly. All poor quality assemblies were removed from the analysis, leaving 3,651 *cps* sequences as well as 162 previously PCR-sequenced isolates. The list of all isolates used in the study is given in supplementary tables S1 and S2, Supplementary Material online.

### Diversity of *Cps* Variants

To compare the genetic similarity of different reference serotypes, we first collected a list of all proteins located within the *cps* locus of all 96 references. Of those, 92 were annotated in the original publications (Bentley etal. 2006; Park etal. 2007; Nahm etal. 2011; Oliver etal. 2013). The four remaining isolates (10X, 11E, 22F, 39X) were annotated by running blastn with -megablast option against *cps* gene sequences from other 92 reference sequences and identifying closely related alleles of *cps* genes. Families of genes with no close resemblance were searched using UniProt (T, O) (Bateman et al. 2015), and if no hits were found they were considered “unknown.” The only exception was the seemingly unknown transferase in 22F: as shown by Salter etal. (2012) this gene is actually *wcwC* described in Bentley etal. (2006) but with no corresponding sequence available due to mistaken submission in the original publication. Of 1,590 genes, we focused on serotype-specific genes, namely those involved in the synthesis of the repeat-unit (i.e., acetyltransferases, glycosyltransferases, flippases and polymerases) and excluded overrepresented genes *wzg*, *wzh*, *wzd*, *wze*, *dexB*, *aliA*, *aliB*, sugar synthesis genes and transposable elements, giving altogether 742 proteins. All protein sequences were then classified into homology groups with a similar approach to Mavroidi etal. (2007). Specifically, all-versus-all blastp was run with e-value threshold of 10^−50^ with hits with <60% query coverage ignored. The resulting undirected network was analyzed with MCL (Enright etal. 2002) with inflation value of 2, resulting in 254 homology groups. A sequence similarity network was constructed using 96 reference serotypes with nodes representing reference isolates and edges representing similar sequences. Similarity between two isolates was defined as *s* = *max*(*s*_*A*_,*s*_*B*_), where *S*_*i*_ is the proportion of homology groups shared by both isolates to the number of homology groups in isolate *i*. A network was built for a given similarity threshold meaning that all pairs above a chosen similarity threshold were connected with an edge. A conservative similarity index of 0.58 was chosen to maximize the number of serogroups which are internally connected and to minimize the number of serogroups which are externally connected (see supplementary fig. S40, Supplementary Material online). This approach produced 40 clusters which were used as a basis for defining a genetic similarity group. Using such clustering, we identified 12 groups which had at least 40 isolates and 500 single nucleotide polymorphisms (SNPs) in the alignment (as given in main text). With the exception of serogroup 19, these genetic similarity groups were identical when defined on the basis of significant shared similarity with threshold of *e *=* *0.01 based on the approach used by Lima-Mendez etal. (2008).

### Evolution of Serogroups

Genetic serogroups were initially aligned using progressiveMauve (Darling etal. 2010). Visual inspection of these alignments, together with the detailed analysis of the genetic content of serotypes in question and the identification of homologous and nonhomologous regions for each pair of reference strains by the command blastn-taskmegablast, led to determination of sequence blocks in the alignment. Each block corresponded either to a homologous gene or group of genes in the reference, or to insertions or duplications. These blocks were aligned using mafft with -ginsi option (Katoh etal. 2002) and concatenated to produce the full alignment, such that the full *cps* genetic diversity was used in further analyses.

To infer the population genetic structure of serogroups, the STRUCTURE software with linkage model was used (Falush etal. 2003). The runs were based on at least 600,000 iterations plus 200,000 burn-in and with multiple chains to insure convergence. The number of populations *K* was found as the smallest value of *K* which explained the observed population structure, and was supported by all independent runs, and by BAPS (Corander and Marttinen 2006). In all examined cases, the identified populations corresponded well to major serotypes or serotype groups (see Supplementary Material online for the results of the population structure analyses). Between-population recombinations, defined by STRUCTURE with the minimum posterior probability for originating in a different population as 0.75 and reaching at least 0.95 at one site, were removed. The initial phylogeny was generated using PhyML (Guindon etal. 2010) with the GTR model of nucleotide substitution with four substitution rate categories. Next, each population was analyzed by Gubbins (Croucher, Page, etal. 2015) with the initial phylogeny used as a starting tree. Recombinations identified by Gubbins or STRUCTURE were removed. The resulting clonal alignments were then analyzed again by STRUCTURE to identify potential hierarchical population structure and within-population recombinations which Gubbins could not detect. In the final alignment, all regions identified by STRUCTURE or Gubbins were removed from the alignment to generate the final clonal phylogeny. The pattern of recombinations on this tree was predicted by both Gubbins (running a single iteration conditional on the final phylogeny with two window sizes: default 10 kb and optional 1 kb to detect shorter recombinations) and STRUCTURE (using the ace function in ape package in R to predict the most likely ancestral pattern; Paradis etal. 2004). The two types of recombinations were then merged into the final list of recombinations with overlapping blocks merged, however, because Gubbins has a more elaborate algorithm of predicting ancestral recombinations based on ancestral SNP reconstruction, STRUCTURE-recombinations at internal nodes which did not overlap with Gubbins-recombinations were ignored. All recombinations ancestral to each of the *K* populations found were ignored due to low detection power of events on long tree branches.

### Molecular Clock

To estimate the molecular clock of the *cps* locus, we focused on the three globally disseminated PMEN lineages with recombinations removed as described earlier (Croucher etal. 2011; Croucher, Chewapreecha, etal. 2014; Croucher, Hanage, etal. 2014). Alignment was divided into the *cps* locus (defined by the starting position of the *dexB* and the ending position of the *aliA* gene) and background (the clonal alignment with *cps* removed). The two alignments were then analyzed using BEAST2 (Bouckaert etal. 2014) in a single analysis with parameters shared between alignments. In particular, we assumed that the two alignments had the same substitution model (GTR with four gamma categories), the same tree prior (Coalescent Bayesian Skyline), but different parameters of the clock model (Relaxed Clock Log Normal). We ensured that all parameters were estimated with effective sample size (ESS) >200. The results of the ucldMean parameter for the *cps* from three different lineages were then pooled together to which a log normal distribution was fit, yielding best fit parameter of μ = −12.98, which was used as the underlying molecular clock rate of the *cps* (see below).

To estimate the null distribution of the molecular clock rate in the background (i.e., without *cps*) in each of the three lineages, we did the following. First, we randomly sampled 20 regions from the clonal alignment (excluding *cps*). Each region contained a coding sequence as predicted by the Prodigal software (Hyatt etal. 2010) plus the intergenic region between the next coding sequence. (This way the overall length of the sampled regions was approximately the same length as the *cps*, and the total length of all regions was equal to the length of the whole-genome alignment without the *cps*.) Second, the 20 sampled regions were concatenated, to which a strict clock model was fitted using BEAST2 with the same substitution model as above but conditional on the clonal frame, and ran for 20M iterations. Third, we repeated this procedure 1,000 times, for all three lineages, and saved the mean value of the clock rate for each lineage. Finally, the molecular clock of the *cps* was obtained in the same way but using only the *cps*, as defined above, instead of the 20 concatenated regions.

To date the clonal trees for the 12 serogroups, we analyzed their clonal alignments using BEAST2. Specifically, the same set of models was fitted as in the case of whole genomes with three exceptions. First, we assumed a coalescent constant population tree prior. Second, an informative prior for the simpler, molecular (strict) clock was used with the value of μ estimated using whole genomes and the standard deviation of 0.04. Third, the phylogeny was fixed as the final clonal phylogeny obtained in the previous section, thereby estimating divergence times and the dates for the recombination events assigned to each node of the tree.

### Selection

Estimation of ω = *dN*/*dS* for three PMEN lineages was performed using CODEML from the PAML 4.9 package (Yang 2007). First, we compared ω of the *cps* to the average omega in the genome. To this end, we concatenated genes as predicted by Prodigal in the *cps* versus everywhere else. Two sets of genes were removed: (1) loci with alleles containing stop codons and (2) loci with >20% of uninformative sites, where an “uninformative site” was defined as the one where 80% or more of isolates had missing data. CODEML was fit conditional on the whole-genome tree and with κ = 2 and α = 0. Confidence intervals were estimated using the log-likelihood ratio test. Second, we estimated ω value for each gene, which was done by running CODEML individually on each gene, excluding those genes which were removed in the first approach. Calculation of the number of synonymous and nonsynonymous substitutions was performed by inferring the pattern of ancestral polymorphisms on the clonal frame using ClonalFrameML (Didelot and Wilson 2015). To obtain the null distribution of the ω values, we performed an analogous calculation as in the case of the molecular clock. Specifically, we randomly selected 20 coding regions from the background excluding the *cps* locus, and estimated ω by concatenating these sequences. The procedure was repeated 1,000 times.

### Origins of Recombinations

To identify the sources of recombinations, we only focused on the more recent recombinations, i.e., those occurring within each of the sub-populations identified for each serogroup alignment. The recombination sequences were exported using the SNP ancestral pattern predicted along the branches of the maximum likelihood phylogeny with Gubbins. Similarity between two recombinations was defined by the blast search with minimum e-value of 10^−20^ and percentage-identity of 90%. The resulting recombinations were then blasted against the entire database of *S. pneumoniae* and other streptococcal isolates listed in supplementary tables S1 and S2, Supplementary Material online. All taxa found downstream of the node where the recombination was found were excluded from the hit list. As a null hypothesis, we assumed that the source of recombination is unknown unless a hit was found. If a single serotype was identified as the origin it was assigned a weight of one; if multiple serogroups were recorded as hits, the weight was one divided by the number of serogroups.

### Lineage-Jumping Dynamics

To estimate a lineage-jumping rate for the four major serogroups (6, 19, 23 and 14/15), we first defined clonal complexes (CCs) and clonal complex groups (CCGs) using eBurst (Feil etal. 2004). To this end, we downloaded all available sequence types from pubmlst.org/spneumoniae (in July 2016) and identified a CC with 6/7 MLST-locus identity, and a CCG with 5/7 MLST-locus identity. Having identified a CC for each isolate, CCs were treated as discrete traits to perform a discrete trait phylogenetic analysis (Lemey etal. 2009) by modeling switches of the genomic lineage background (CC or CCG) as discrete trait substitutions. As sampling of isolates was blind to the genomic background (with the exception of PMEN isolates which were removed from this analysis), and as our discrete traits represent genetic, not geographic traits, no sampling bias for the discrete trait analysis was expected (De Maio etal. 2015). We also did not expect any bias due to the distribution of lineages in different countries as such bias would only be expected in the most extreme case of a single genomic background present in each geographic location but different backgrounds in different locations (this was not the case due to the nature of data collections analyzed here). Each sub-population in a given serogroup (determined by STRUCTURE/BAPS) was considered as a separate phylogeny with shared molecular clock and the shared jump-rate between phylogenies within the same serogroup. For simplicity, a homogeneous jumping-rate was assumed.

### Estimation of Recombination Rates

In order to estimate the recombination rate for each serogroup, we fitted a basic model describing the distribution of recombination events on a tree using a Poisson process; see also Mostowy etal. (2014). The number of recombinations at each branch of the tree was modeled as a Poisson-distributed random variable *m*_*i*_ with mean λ*L*_*i*_, where λ is the inferred recombination rate and *L*_*i*_ is the branch length in years. The estimated recombination rate λ was the value which maximized the likelihood $\ln\prod_{i=1}^{B}\;\text{Pois}(m_{i},\lambda L_{i})$, where *B* is the number of tree branches. To avoid estimate bias, we excluded all long branches of the tree, namely branches leading to the most recent common ancestors for each subpopulation (of the *K* populations estimated by STRUCTURE) together with all their ancestor branches. The same procedure was applied to both serogroup trees and lineage trees.

To compare recombination rate at the *cps* with whole-genome recombination rate, we analyzed the distribution of recombinations on lineage-derived trees, as described earlier. To control for capsular switches, we excluded lineages which were not predominantly of the same serotype or serogroup, depending on the analysis. Genes *dexB* and *aliA* were used to identify the *cps* coordinates and distinguish background- from *cps*-recombinations. The list of all lineages is given in supplementary table S5, Supplementary Material online. The lineages used to estimate the recombination rates in the four analyzed serogroups were chosen as predominantly of the same serotype (minimum 80%). Serogroup 6: MA-10, MA-13, MA-14 and PMEN2; serogroup 19: Maela-1 and PMEN14; serogroup 23: PMEN1; serogroup 14/15: MA-3 and Maela-7. (The remaining Maela lineages were ignored due to the absence of the full resolution *cps* locus in the original alignment.) For each serogroup, lineages were used collectively to estimate the mean recombination rate by combining the information about the number of recombination events on each branch and the corresponding branch length in units of years, and fitting the Poisson model described earlier.

## Data Availability

All *cps* sequences generated and used in this study, including reference sequences, have been made publicly available online, and are available via the following Figshare DOIs: https://doi.org/10.6084/m9.figshare.4681207.v1, https://doi.org/10.6084/m9.figshare.4681213.v1, https://doi.org/10.6084/m9.figshare.4681225.v1. Supplementary figures S7, S9, S11, and S13, Supplementary Material online, are available in high-resolution via: https://doi.org/10.6084/m9.figshare.4680913.v1, https://doi.org/10.6084/m9.figshare.4681180.v1, https://doi.org/10.6084/m9.figshare.4681198.v1, and https://doi.org/10.6084/m9.figshare.4681201.v1.

## Supplementary Material

Supplementary data are available at *Molecular Biology and Evolution* online.

## Supplementary Material

Supplementary Data
