# Supplementary material for: Pneumococcal Capsule Synthesis Locus cps as Evolutionary Hotspot with Potential to Generate Novel Serotypes by Recombination
Source: Mol Biol Evol. 2017 Jun 8;34(10):2537–54. doi: 10.1093/molbev/msx173 (PMC5850285; doi:10.1093/molbev/msx173)
Supplement: Supplementary Data [file msx173_Supp.zip › TextS1.pdf]

Supplementary Material

1 Additional details

Serotyping *in silico*

Genetic serotyping of all isolates was done by comparing nucleotide and amino-acid sequences of individual genes to the ones described in a set of reference genes [1], and several updated polysaccharide structures (see citations below). First, each sequence was blasted against a set of glycosyl and acetyl transferase genes (throughout the study referred to as serotype-specific genes) with the cutoff of $e = 10^{-50}$. Candidate serotypes were chosen as those whose all serotype-specific genes were found in the query sequence. If multiple candidate serotypes were found, the actual serotype was chosen as the one with a higher similarity to the corresponding reference sequence, with the exception of serotypes which were fully syntenic (i.e., those whose genes had an identical set of gene homology groups). Out of 19 syntenic groups, 17 were found in the dataset: 6A/6B/6F/6G, 6C/6D, 7A/7F, 7B/7C/40, 9A/9V, 9L/9N, 11A/11D/11E/11F, 11B/11C, 12A/12B/12F/44/46, 15B/15C, 18B/18C, 19A/19F, 24B/24F, 25A/25F/38, 28A/28F, 32A/32F, 33A/33F/37, and 35A/35C/42; the two absent groups being 11B/11C and 41A/41F. Determining the actual serotype within each synteny was done as described in Table S3.

Seven isolates had a full capsular locus identified in spite of having been classified as a non-typeable. In three of these cases (ERR051587, ERR063956, ERR064096) the identified serotype was putative 39X (see next section). Of the four remaining cases, three (ERR054582, ERR064226, ERR065309) had sequences identical to other, viable isolates (6C, 15B, 19F) and one was identified as a non-functional 34 (ERR273501).

Confirmation of presence of novel 39X polysaccharide

In addition to three 39X isolates mentioned above, three further were identified in the same population (MaeLa refugee camp). All five carried the same *cps* locus. This name reflects the latex agglutination reactions from the strains at the time of isolation, three of which were non-typeable and two of which appeared to be serotype 21. The five strains were recovered from carriage in three individuals between 2008-2010. Three of the strains (ERR051587, ERR054600, ERR067827) were successfully cultured at the Wellcome Trust Sanger Institute and confirmed to contain the unique *wzy* (polymerase) and *wzx* (flippase) genes of the 39X locus using the following primers:

F: `GTGCTAATTCATTCTAGTACG`

R: `TCCTTAGTTCCATAGCTAGCAACC`

yielding an approximately 2kb product. These three strains were sent for confirmatory serotyping at the *Neisseria* and *Streptococci* Reference laboratory at the Statens Serum Institut, Copenhagen, Denmark. A weak reaction was found by the Quellung method with omniserum, suggesting that the capsule is different to the standard pneumococcal serotypes. A strong reaction was observed with Immulex pool E (which includes type 21, 39, group 10, 12 and 33) and a sporadic specific reaction to 10A occurred; however there was no reaction to pool S (type 5, 8, group 10, 15 and 17). From this we can conclude that pneumococci with the 39X cps locus are capable of producing a novel capsular polysaccharide, and that it may have some cross-reactivity to existing pneumococcal sera.

Neutral model of distribution of recombinations across capsule

Let us assume that the *cps* locus is of length L_C and a recombination is of length L_R. The number of all possible ways in which the recombination can affect the capsule is $L_C + (L_R - 1)$, while the number of ways in which the recombination will be contained within the capsule is $L_C - (L_R - 1)$. Assuming that raw recombination rate is uniform across the locus, the proportion of recombinations contained within the capsule is

$$p(L_R) = \begin{cases} \frac{L_C-(L_R-1)}{L_C+(L_R-1)} & \text{if} \quad L_R \leqslant L_C \\ 0 & \text{if} \quad L_R > L_C. \end{cases}$$

Assuming that the length of recombination events is geometrically distributed with mean R, the frequency of a recombination event of length L_R will be

$$f(L_R; R) = \left(\frac{1}{1+R}\right)\left(\frac{R}{1+R}\right)^{L_R}.$$

The proportion of all recombinations contained within the capsule will thus be

$$\sum_{L_R=0}^{\infty} p(L_R) f(L_R; R) = \left(\frac{1}{1+R}\right) \sum_{L_R=0}^{L_C} \frac{L_C - (L_R - 1)}{L_C + (L_R - 1)} \left(\frac{R}{1+R}\right)^{L_R}.$$

For the mean capsule length of $L_C = 17$kb and mean recombination length of $R = 7.7$kb, this model predicts that 49.6% of all capsular recombinations will be contained within the capsule.

To estimate the ratio between the within-*cps* recombination rate and the full-*cps* recombination rate, here defined as ρ, we defined the following likelihood

$$l(\lambda^{\text{out}}, \rho) = \prod_{i=1}^{B} \text{Pois}(m_i^{\text{out}}, \lambda^{\text{out}} L_i)\text{Pois}(m_i^{\text{within}}, \rho\lambda^{\text{out}} L_i/(1-\rho)),$$

where m_i^{within} was the number of within-*cps* recombinations on branch i, m_i^{full} was the number of full-*cps* recombinations on branch i, and $m_i^{\text{out}} = m_i^{\text{full}} - m_i^{\text{within}}$, B was the number of branches, and

$$\rho = \frac{\lambda^{\text{within}}}{\lambda^{\text{full}}} = \frac{\lambda^{\text{within}}}{\lambda^{\text{within}} + \lambda^{\text{out}}}.$$

Maximum likelihood approach was applied to calculate best fit λ^{out} and ρ. The confidence intervals for ρ were obtained using the log-likelihood ratio test, and significance of the heterogeneity in ρ between different serogroups was tested using one-way ANOVA.

2 Evolutionary history of major serogroups.

Here we summarise the main findings of the phylogenetic and population structure analyses for each of the examined serogroups, and what we can learn from it about their evolutionary history.

Serogroup 6

Serogroup 6 consists of 6 serotypes: 6A, 6B, 6C, 6D, 6F and 6G. The serotype 6B comes in two classes, class I and class II [2] and it has been hypothesised that 6B-II may constitute a new serotype, 6E [12],

however it was recently demonstrated that 6B-II has all the serological and biochemical properties of 6B [13]. From the point of view of the genetic content, the serogroup divides into two main groups, 6A/B/G/F and 6C/D . The two groups differ in the copy of the wciN allele, perviously referred to as $wciN_\alpha$ (A/B/G/F) and $wciN_\beta$ (C/D). The differences between serotypes within each of the groups have been identified as due to single mutations. However, in line with previous knowledge, the population genetic structure analysis reveals that serogroup 6 consists of three populations: class I 6A/B/G/F (pop1), 6C/D (pop2) and class II 6B/6A (pop3). The populations are well defined in the *wzg*-*wzx* region but not in the rhamnose region – there we observe a striking amount of recombination, suggesting that the rhamnose genes are in weak linkage disequilibrium with the remaining capsular genes. Thus, in this particular case, we analysed the region *wzg*-*wzx* separately, and then proceeded with the analysis for the entire capsular region conditional on the *wzg*-*wzx* clonal phylogeny.

The results of the analysis support previous findings that the population 3 (class II) forms an outgroup to the 6A/6B-I/C/D/G/F clade [2]. The diversity within population 3 is mostly due to a few acquired recombinations, two of which led to the emergence of 6As from 6Bs; otherwise the clade has comparatively little diversity. Given that the examined population 3 isolates come from 13 countries sampled over 23 years, it suggests that this capsule variant could have evolved in another bacterial species, for example *S. mitis*, and was acquired relatively recently into the pneumococcus. The split between 6A/6B-I and 6C/D is more recent and likely occurred in the same species. The higher density of SNPs flanking $wciN_\beta$ identified as two recombinations around that gene suggests that this gene was inserted via illegitimate recombination into the 6C/D ancestor, in line with earlier findings [14]. The source of this recombination is unclear, however we found a close copy of the $wciN_\beta$ gene in putative serotype 39X suggesting that this gene spread to both serotypes in the past. The analysis also suggests that in class I 6B emerged from 6A once by acquiring a mutation, and likewise that 6F and 6G emerged by acquiring relevant substitutions in $wciN_\alpha$. In contrast, we see clearly that 6D emerged via recombination spanning the wciP, wzy and wzx genes and that this recombination originated in pop1 (6A/B-I clade). Even though the final clonal tree suggests that 6D may have evolved twice, the recombination underlying the diversification is the same while the clade uncertainty is high due to a small number of SNPs remaining after removing the recombination. Thus, a much more parsimonious explanation is that 6D emerged once by a single recombination event. This provides support for the previously hypothesised scenario [15]. Finally, the analysis supports the emergence of the US mosaic isolate abbreviated 7001_1#23-s6b as a result of recombination between populations 1 and 3.

Overall, this analysis provides strong evidence against the notion that 6A/6B emerged once, clearly demonstrating multiple emergence of 6B from 6A and 6A from 6B in different clonal backgrounds. It also provides evidence that many times new serotypes originated by recombination, including those examples when the difference between serotypes is due to a single substitution, like in the case of 6A and 6B. We thus reconcile previous, seemingly paradoxical, observations [2, 14, 16, 17], and suggest a new, more complex model of evolution of serogroup 6. The population genetic structure is shown in Figure S6, and the evolutionary diagram with corresponding recombinations is shown in Figure S7.

Serogroup 19

Serogroup 19 consists of 4 serotypes: 19A, 19B, 19C and 19F. Additionally, we analysed a capsular sequence AEDU-mitis from the closely related species *Streptococcus mitis*, which bears close resemblance to 19C serotype [18]. 19B is very similar to 19C/AEDU in that it misses a wchU gene. The two remaining serotypes, 19A and 19F, additionally possess wchR, wchS, rbsF transferases as well as non-related copies of flippase (*wzx*) and polymerase (*wzy*). Using this knowledge, Kilian and colleagues suggested the following model of evolution of serogroup 19 [19]: the 19C-ancestor capsule was acquired from *S. mitis*, and then the ancestor of 19A/F emerged by the horizontal transfer of wchR, *wzy*, wchS, rbsF and *wzx*, and further diversification of 19A and 19F into different serotypes. Meanwhile, 19B emerged

by the loss of *wchU*. However, our analysis does not support this model, and in turn suggests that a more complicated scenario.

The population genetic structure analysis shows that 19A is so diverse from all other serotypes that it forms an outgroup to all remaining capsular sequences. The other populations are 19F, 19B/C and a single *S. mitis* strain which forms a separate population. However, 19F is much more closely related to 19B/19C than to 19A. Therefore, the more likely explanation for the observed pattern of clonal diversity is that the split between 19A and the ancestor of all remaining serotypes was the first one to occur. Later, the 19B/19C cluster emerged by recombination between 19F and a *S. mitis* AEDU-like capsule giving rise to 19C serotype. Our analysis also suggests that 19C forms an outgroup to 19B strains, suggesting that 19B probably evolved from 19C by the loss of gene *wchU*, likely via homologous recombination. The population genetic structure and the evolutionary diagram are shown in Figures S8 and S8, respectively.

Serogroup 23

Serogroup 23 consists of 3 serotypes: 23A, 23B and 23F. Serotypes 23B and 23F have an identical gene content and they differ from 23A by the presence of a different polymerase gene (*wzy*). Our analysis clearly identifies three distinct genetic populations which form three monophyletic clades, however we find a considerable amount of population mixing at downstream glycerol and rhamnose genes, possibly due to recombination. The population 23B consists of two subpopulations, one of which features a divergent set of downstream genes *wchX*, *gtp1*, *gtp2*, *gtp3*, *rmlA*, *rmlC* and *rmlB* (but not *rmlD*). The most parsimonious explanation is that the serotype diverged clonally and then reacquired a recombination from 23F however the phylogenetic analysis shows that the isolates with divergent genes downstream form a monophyletic subclade, and thus it seems likely that the divergent recombination came from from an unknown source. This suggests that all three serotypes (except the 23B subclade) are closely related downstream in the alignment and divergent upstream in the alignment. Interestingly, the diversity is not uniformly distributed as with the exception of highly divergent *wzd*, *wze* and *wchA* genes in 23A, 23A is much more closely related to 23F than it is to 23B.

One explanation for this is that 23A and 23F diverged most recently by 23A acquiring (a) a copy of wzy from unknown source and (b) diverse genes wzd, wze and wchA which bear close resemblance to serotypes 18A/16F. In this case, the divergence between 23B and the ancestor of 23A/23F occurred further down the past. The close resemblance of downstream region of the capsule across all serotypes suggests further recombination events between the three serotypes, however the precise evolutionary scenario remains unclear. The case of serogroup 23 again emphasizes the importance of recombination in the evolution of polysaccharide loci. The population genetic structure and the evolutionary diagram are shown in Figures S10 and S11, respectively.

Serogroups 14 and 15

Due to the close genetic relatedness, serotype 14 and serogroup 15 have been analysed together as serogroup 14/15, with serotypes 14, 15A, 15B, 15C and 15F. All five serotypes have an identical gene content in the region *wzg*-*wzx* with the exception of 15A/F which contain a different copy of polymerase *wzy*. 15A differs from 15F in that is has inactive gene *wciZ*, and similarly for 15B/C. Serotype 14 is the most diverse from all others and it also contains a *wciY* gene following *wzx* and a repetitive *lpr* sugar transferase. The population structure analysis identified 3 populations: 14, 15A/15F and 15B/15C. The phylogenetic analysis shows that the serotype 14 forms an outgroup to serogroup 15, indicating that the serological difference between the two serogroups is reflected at the genetic level. The divergence between the two serogroups likely occurred by the exchange of *wciY* and *lpr* genes (present in serogroup 14) to *wciZ*, *wchX*, *gtp1*, *gtp2*, *gtp3* (present in serogroup 15), or vice versa. The more recent split occurred between the 15B/C cluster and the 15A/F cluster, very likely by the

ancestor of the 15A/F group horizontally acquiring a different polymerase gene together with several upstream genes including wchA, wchJ and wchK. The analysis also suggests a single emergence of the 15A serotype from 15F as a frameshift mutation as all 15A's form a monophyletic clade, also in the phylogeny based only on the wciZ gene. However, the inverse could also be true: 15F could have emerged from 15A having acquired a recombination from 15B/C-like ancestor. Interestingly, we see multiple emergence of 15B from 15C and vice versa, however it is not clear whether genetic alterations between active and inactive wciZ are due to frameshift mutations, homologous recombinations, or both. Finally, the presence of rmlB, rmlD and glf genes in 15F remains unclear. It could be that this serotype acquired it relatively recently, or alternatively it could be a remnant of old rhamnose genes synthesised by some ancestors of 15F. To better understand this a bigger data sample of serogroup 15 is needed. The population genetic structure and the evolutionary diagram are shown in Figures S12 and S13, respectively.

Serogroup 18

Serogroup 18 consists of 4 serotypes: 18A, 18B, 18C and 18F. Additionally, we analysed a capsular sequence JPFV-mitis from the closely related species *Streptococcus mitis*, which bears close resemblance to 18F serotype. The serotypes mostly consist of homologues glycosyltransferases with the following exceptions: (a) the mitis strain and 18F have a copy of wcxM which the other serotypes are lacking, (b) 18A, unlike all other serotypes, misses wciX, and (c) 18B differs from 18C in that it has an inactive copy of wciX. The phylogenetic analysis supports the most parsimonious scenario of evolution with the exception of 18B, which in our analysis is found to emerge twice. Assuming the mitis isolate to be an outgroup of the serogroup, the 18A/B/C form a monophyletic clade which arose after the loss of wcxM and then diversified into 18A upon the further loss of wciX in the 18B/C clade. The 18B seems to have emerged at least twice from 18C by acquiring two independent frameshift mutations. The population genetic structure and the evolutionary diagram are shown in Figures S14 and S15, respectively.

Serogroup 10

Serogroup 10 consists of 4 serotypes: 10A, 10B, 10C and 10F; additionally we analysed a newly discovered variant termed 10X, a candidate new serotypes. 10X is genetically similar to 10F however it contains a different set of upstream cpsABCDE genes, with wchA replacing wcjG as the initial sugar transferase, an partially disabled copy of wzy gene with over 50% of it missing, and three inactive homology groups of unknown source, possibly remnants of a single gene. The remaining serotypes cluster into two groups, with 10A/B being one and 10C/F being another. The two groups differ in the presence of wcrG (absent in 10C/F), wcrH (absent in 10A/B) and the presence of a working copy of wcrD (inactive in 10C/F). Furthermore, the serotypes differ genetically at the wcrC gene with 10B/F/X having an allele which was shown to alter the biochemical structure of the capsule, and termed wcrF [20]. Consistently with these observations, our analysis found 4 genetic populations: 10X, 10A, 10B and 10C/F, with two mosaic 10A/10B sequences which arose independently. Further phylogenetic analysis suggests that the likely evolutionary scenario of serogroup 10 emergence is consistent with the most parsimonious explanation, however the direction of gain/loss of genes is unclear. One possibility is that the emergence of the 10C/10F clade was cause by the loss of wcrG, gain of wcrH and deactivation of wcrD. This is supported by the monophyletic 10C/F clade of the wcrD gene-based tree alone. 10A would then emerge from 10B in the same way as 10C would emerge from 10F, namely by evolving wcrF into wrcC. As wrcC in 10C and 10A are nearly identical, at least one of these events had to be a recombination event, and this is supported by the population genetic structure analysis of the serogroup. The population genetic structure and the evolutionary diagram are shown in Figures S16 and S17, respectively.

Serogroup 11

Serogroup 11 consists of at least 6 serotypes: 11A, 11B, 11C, 11D, 11E and 11F, however their classification has proved challenging [9, 10]. With respect to gene content serotypes classify into two groups: 11A/D/E/F which contain wcwC allele and 11B/C which contain *wcwR* allele. 11B has the same gene content as 11C but has inactivated copy of *gct*. 11A/D/E/F also have identical gene content but (a) 11E has inactive copy of *wcjE*, (b) 11F has inactive copy of *gct*, while (c) 11A/D have both genes active but differ by mutations in *wcrL*. The population genetic structure has revealed four distinct groups: 11A/D, 11A/E, 11B/C and 11F. One mosaic 11A sequence was found in the Massachusetts collection. The phylogenetic analysis confirms that there are two classes of 11A serotypes, and suggest the following evolutionary scenario. The most recent common ancestor of serogroup 11 diverged into 11B/C group and 11A/D/E/F group, which must have happened via recombination as *wcwC* and *wcwR* are non-homologues. Further split happened between 11A/D and 11A/E/F clades, most likely via vertical diversification, and further split between 11F and 11A/E. Even though 11F have inactive copy of *gct*, it is not certain that the underlying mutation caused the divergence; rather this mutation was acquired in the process of gradual diversification over a longer time. Finally 11E emerged relatively recently by inactivating the *wcjE* gene (11Es form a monophyletic clade in the *wcjE*-based phylogeny), while likely 11D diversified from the first 11A group by acquiring a mutation in *wcrL*. The 11B/C clade diversified relatively recently by 11C acquiring a recombination from 11F. It thus seems likely that there is a substantial amount of uncovered genetic diversity across serogroup 11. The population genetic structure and the evolutionary diagram are shown in Figures S18 and S19, respectively.

Serogroup 9

Serogroup 9 consists of at least 4 serotypes: 9A, 9L, 9N and 9V. They subdivide into two groups with identical gene content: 9A/V and 9L/N, and differ with respect to each other in that the first group contains an extra gene *wcjD* and a transposable element, presumably acquired together. This subdivision is also true with regard to the serogroup population genetic structure, with two groups being genetically distant. This suggests that the split between 9A/V and 9L/N occurred a long time ago, with the *wcjD*/*tnp* gene complex acquired horizontally. The 9L/N group diversified into 9L and 9N, and it is possible this happened by horizontal acquisition of *wcjA* gene from 9V. This hypothesis is supported by Structure analysis which finds a recombination in that gene as well as the fact that *wcjA* copies of 9Ls are more closely related to 9V than they are to 9N. Finally, the relatively low genetic diversity within the 9A/V group suggests a more recent emergence of 9A from 9V (by inactivation of *wcjE*). The population genetic structure and the evolutionary diagram are shown in Figures S20 and S21, respectively.

Serogroup 34/35

Serogroup 34/35 defined here consists of 3 serotypes: 34, 35F and 47F. This group is a subgroup of a large mosaic sub-population of serotypes sharing multiple genes, however these three serotypes share a large number of those and are thus analysed separately. The population genetic structure analysis reveals two sub-populations, 34 and 35F/47F, and indeed, 47F is genetically much closer to 35F than to 34. The two groups differ mostly in the presence of the initial sugar transferase (*wchA* vs. *wcjH*), a copy of *wzy*-polymerase (two different homology groups), and presence of *wciG* (absent in 34). There is substantial genetic diversity between two two groups, and this suggests that 34 diverged from the ancestor of 35F/47F a long time ago, and in this process altered its genetic composition. Interestingly, 34 and 35F are very close genetically in the region of *wcrO*, *wcrC* and *wcrD* genes. One explanation is that these genes have not diversified over time as much as other genes due to purifying selection, however this is unlikely because their copies in other serotypes (e.g., in 33C, 36 or 39) are

more diverse. Therefore it is possible that the the clonal relation between 34 and 35F is misleading and the emergence of the currently observed diversity is a result of a number of horizontal events in their evolutionary history. What is clearer is the emergence of 47F which likely descended from 35F, first by replacing *wcrO* by *whaL* horizontally from an unknown source, and second by acquiring *glf* and *wcjE* horizontally (and likely from different sources: *glf* from 33A, 33F, 35C or 42 and *wcjE* from 47A). The population genetic structure and the evolutionary diagram are shown in Figures S22 and S23, respectively.

Serogroup 16/28

Serogroup 16/28 defined here also consists of 3 serotypes: 16F, 28A and 28F. 28A and 28F are genetically close and for a single group in the population structure analysis, and their gene content is the same, however there is genetic diversity distinguishing them, especially within the *wciU* gene. Compared to serogroup 28, serotype 16F contains a different polymerase (*wzy*) and syntheses a different sugar using *gct* and not *gtp1*, *gtp2* and *gtp3* genes like the 28s. The phylogenetic analysis confirms that 28A/F and 16F are considerably diverse and the two groups likely split a long time ago. The split between 28A and 28F is much more recent, and likely due to a recombination at *wciU* from an unknown source but vertical diversification cannot be excluded. The population genetic structure and the evolutionary diagram are shown in Figures S24 and S25, respectively.

Serogroup 29/35

This serogroup was analysed mostly due to considerable diversity within 35B, however only one isolate of serotype 29 was available, and thus it is difficult to make certain statements about the evolutionary history of the serogroup. Thus the population genetic structure and phylogenetic analyses do not reject the simplest explanation that the two serotypes diversified a long time ago by 35B's ancestor exchanging *wcjH* to *wchA* and acquiring *wciG*. Interestingly, it shows that some of the diversity acquired within 35B arose via recombination. The population genetic structure and the evolutionary diagram are shown in Figures S26 and S27, respectively.

Serogroup 22

This serogroup consists of only two members, 22A and 22F. The serotypes differ only in one gene, 22A having one copy of *wcwC* and 22F having another one, the two being non-homologous as reported before [21]. Our analysis reveals that 22A and 22F are considerably similar and the exchange of gene leading to the emergence of a new serotype, presumably happening by recombination, must have been more recent than for many previously described serogroups. Furthermore, we find two distinct genetic populations of 22F, one from Thailand and the other one from the USA, and the genetic distance between them is comparable to the one between 22F and 22A. This suggests that there may be a considerable amount of uncovered serotypic diversity within serogroup 22. The population genetic structure and the evolutionary diagram are shown in Figures S28 and S29, respectively.

3 Supplementary figures

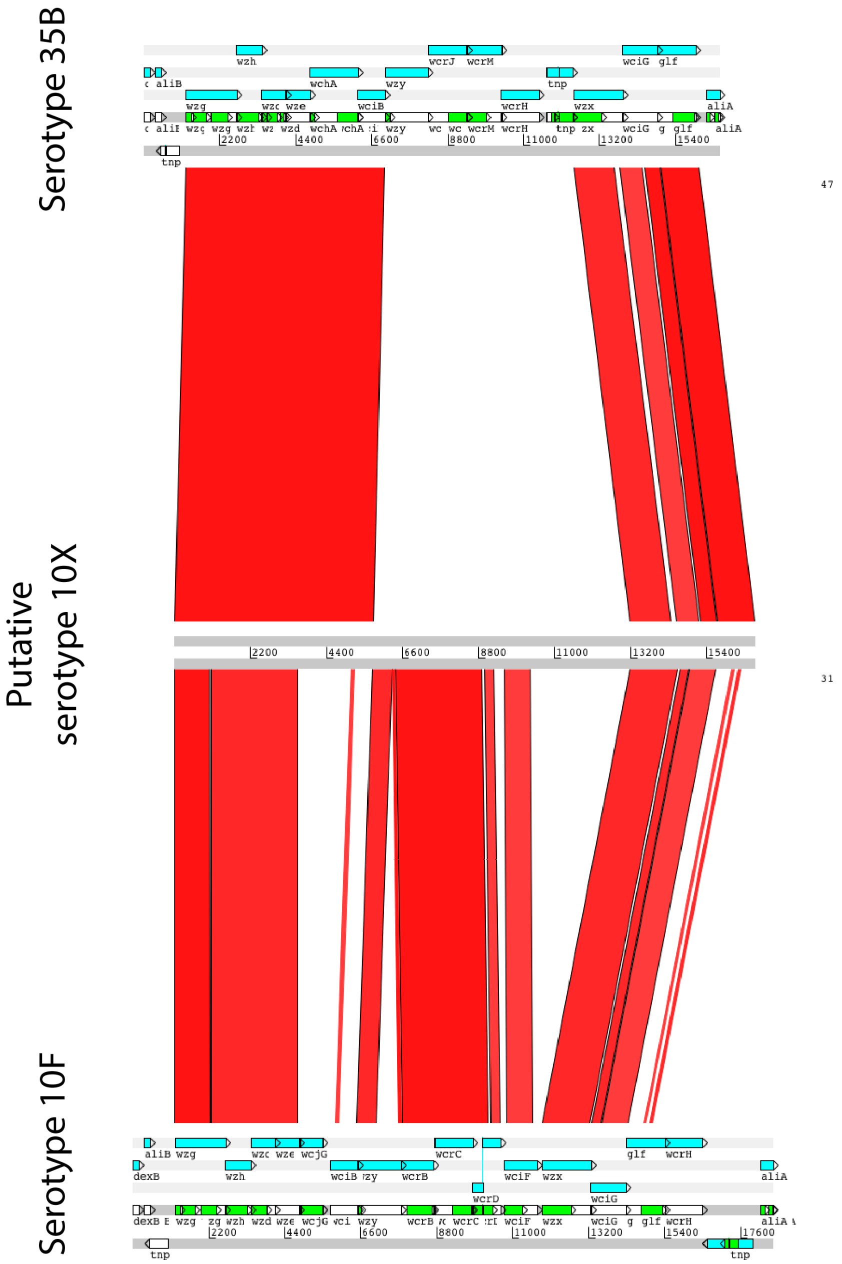

Figure S1. Genetics of putative serotype 10X. A comparison of putative serotype 10X from MaeLa (ERR049999) with two known reference serotypes: 35B (top, CR931705) and 10F (bottom, CR931652). Starting with the 5′ end, the following genes were identified: *wzg*, *wzh*, *wzd*, *wze*, *wchA*, *wciB*, *wzy*, *wcrB*, *wcrC*, *wcrD*, *wciF*, *wzx*, *wciG* and *glf*. The gap between *wciF* and *wzx* corresponds to three unknown homology groups with no hits in the public database.

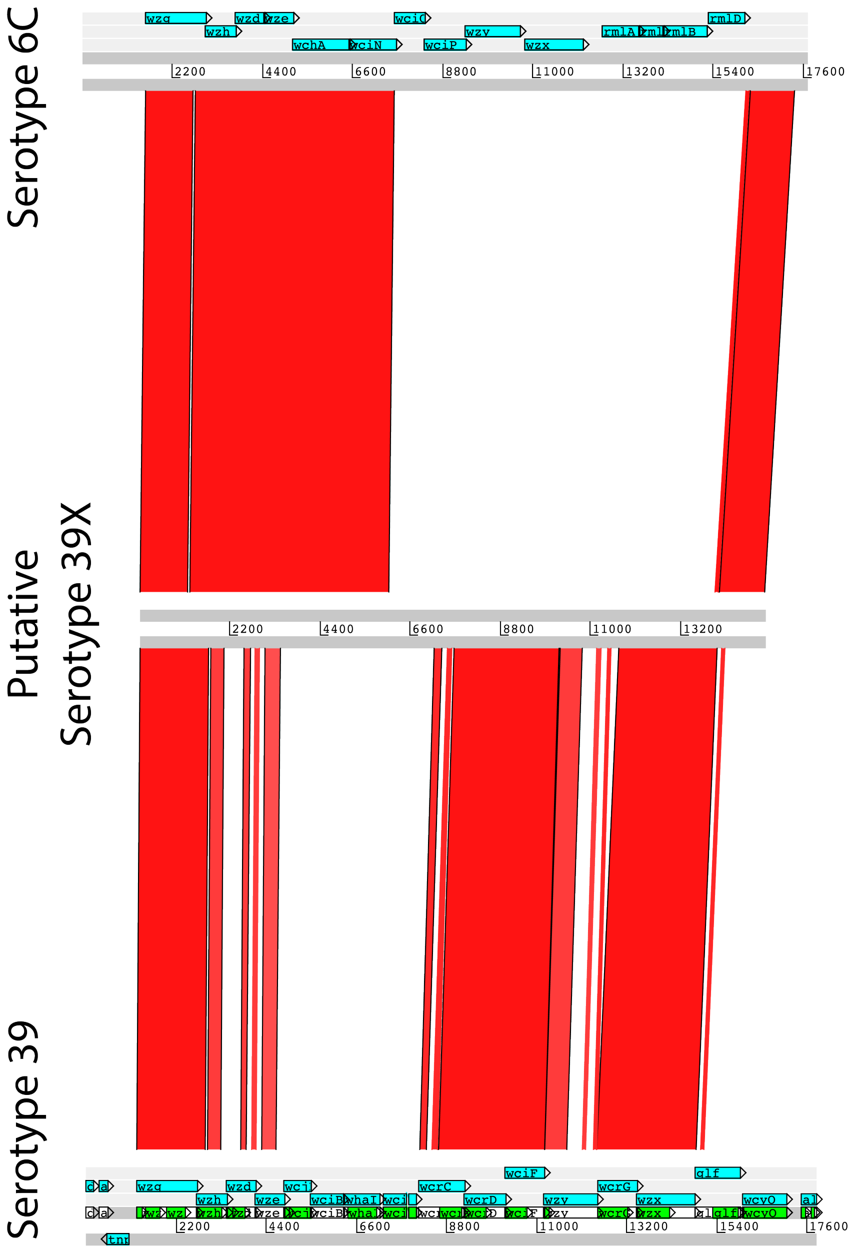

Figure S2. Genetics of putative serotype 39X. A comparison of putative serotype 39X from MaeLa (ERR051587) with two known reference serotypes: 6C (top, EF538714) and 39 (bottom, CR931711). Starting with the 5′ end, the following genes were identified: *wzg*, *wzh*, *wzd*, *wze*, *wchA*, *wciN*, *wcrO*, *wcrC*, *wcrD*, *wciF*, *wzy*, *wcrG*, *wzx* and *glf*. The gap between *wciN* and *wcrO* corresponds to a homology group from an unidentified source.

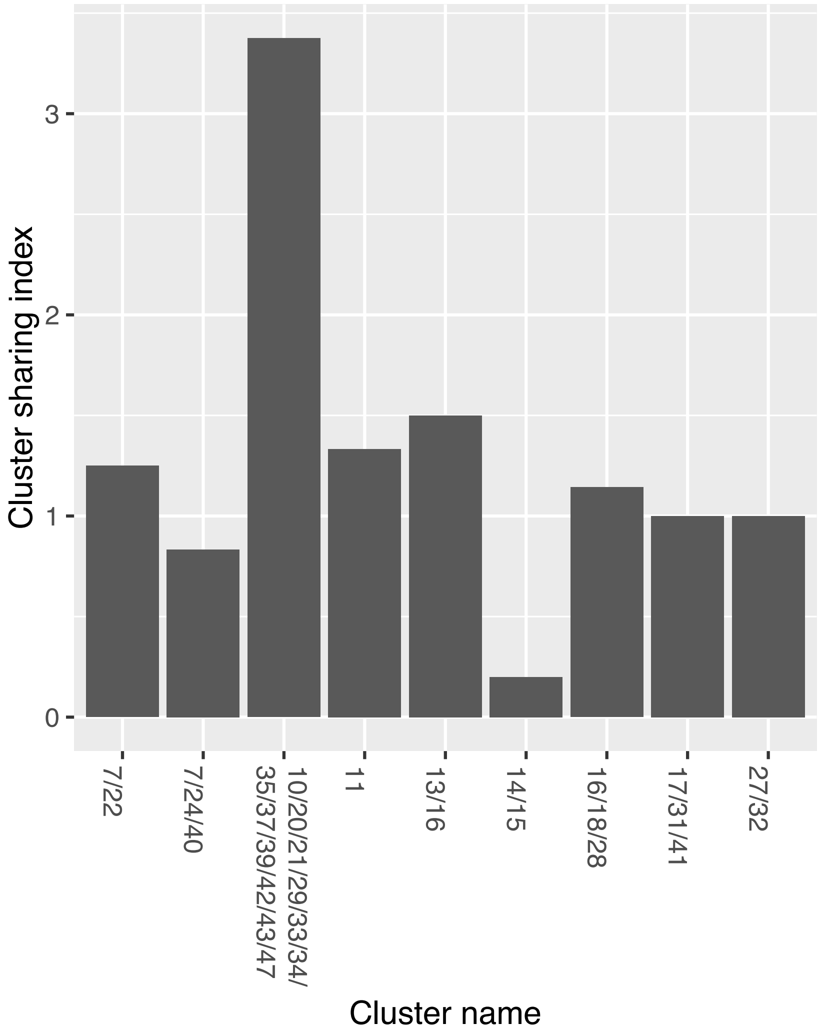

Figure S3. Mosaicism of large serotype clusters. Mean cluster sharing index for liberal serotype clusters defined in Figure 1C (groups with value of zero are not shown). The sharing index for each serotype was calculated by counting the number of genetic serogroups (defined as conservative clusters in Figure 1C) with which it shares at least two capsule-specific genes. The mean cluster sharing index was calculated as a mean sharing index of all nodes (serotypes) belonging to this cluster. The cluster with the largest mean sharing index is the largest cluster in Figure 1C, and on average its members share at least two genes with 3.4 other genetic serogroups.

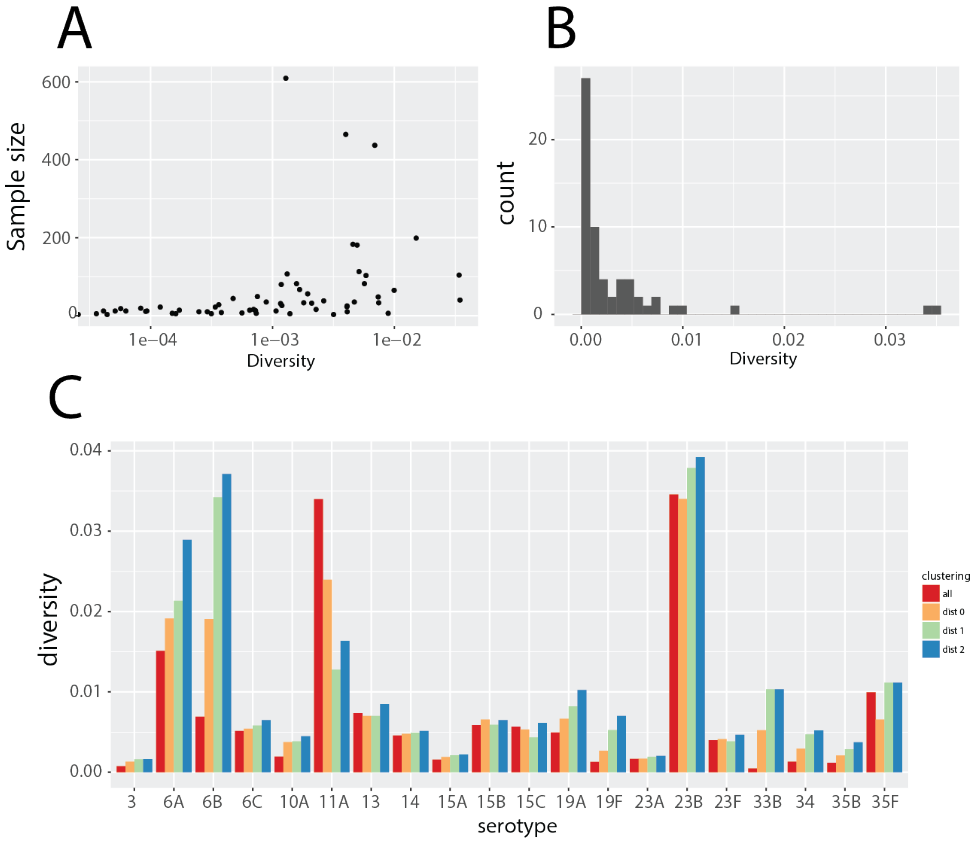

Figure S4. Within-serotype diversity. (A) Mean pairwise diversity vs. sample size for all serotype alignments (prior to removing recombinatioins) with at least 3 isolates, giving 61 data points altogether. Diversity was calculated as the mean pairwise Kimura K80 distance within a sample. (B) Histogram of diversity shown in (A). (C) Mean pairwise Kimura K80 diversity in the 20 largest serotype alignments. Four different clustering methods were used: none (red), grouping isolates of distance $d = 0$ (yellow), grouping isolates of distance $d = 1$ (green), and grouping isolates of distance $d = 2$ (blue). Diversity was then calculated based on the representative set (one random isolate per cluster).

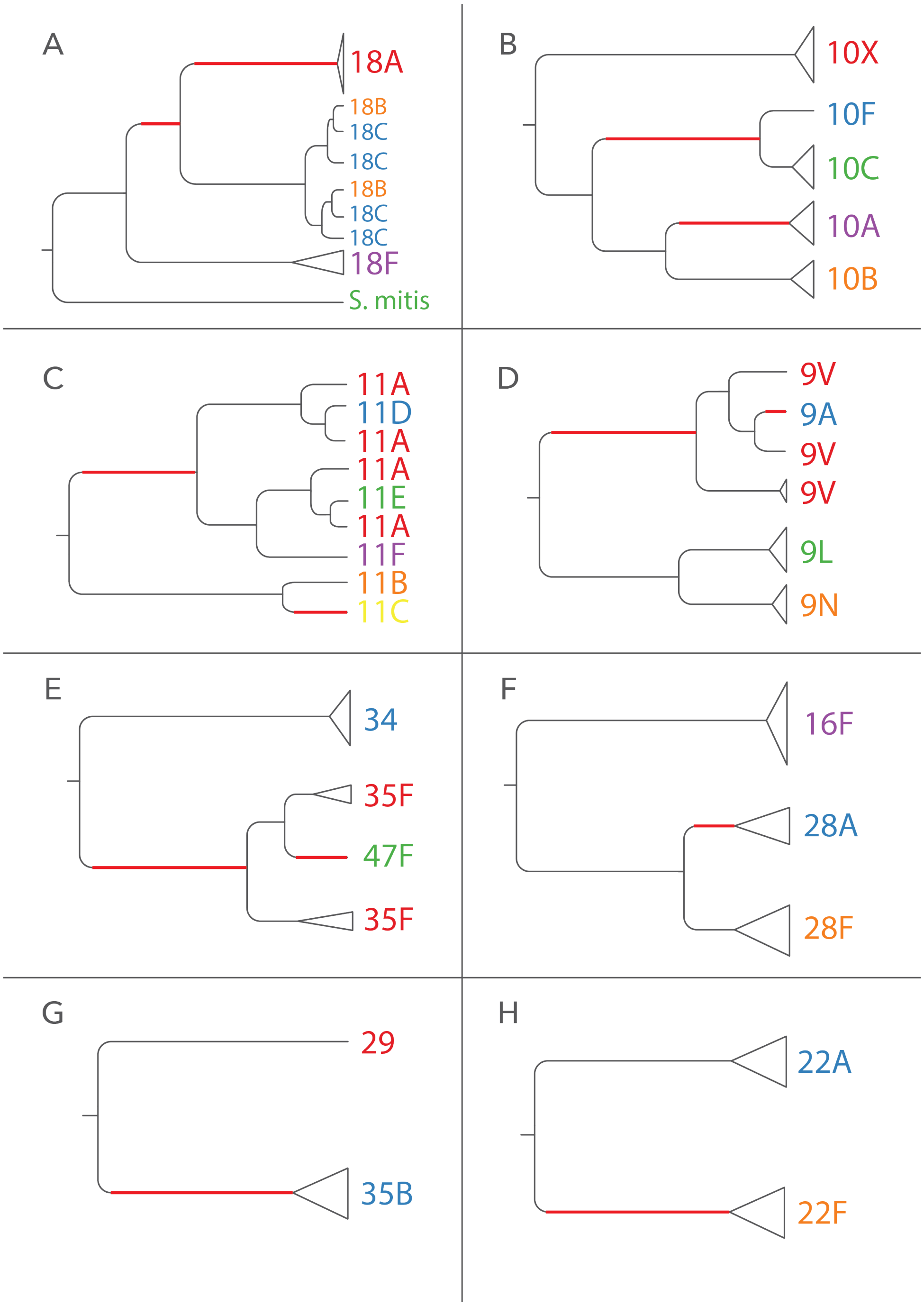

Figure S5. Evolution of serogroups 18, 10, 11, 9, 34/35, 16/28, 29/35 and 22. Dendograms showing schematic evolution of eight serogroups in question. Recombinations hypothesised to have occurred on branches leading to a new serotype or a mosaic are coloured in red.

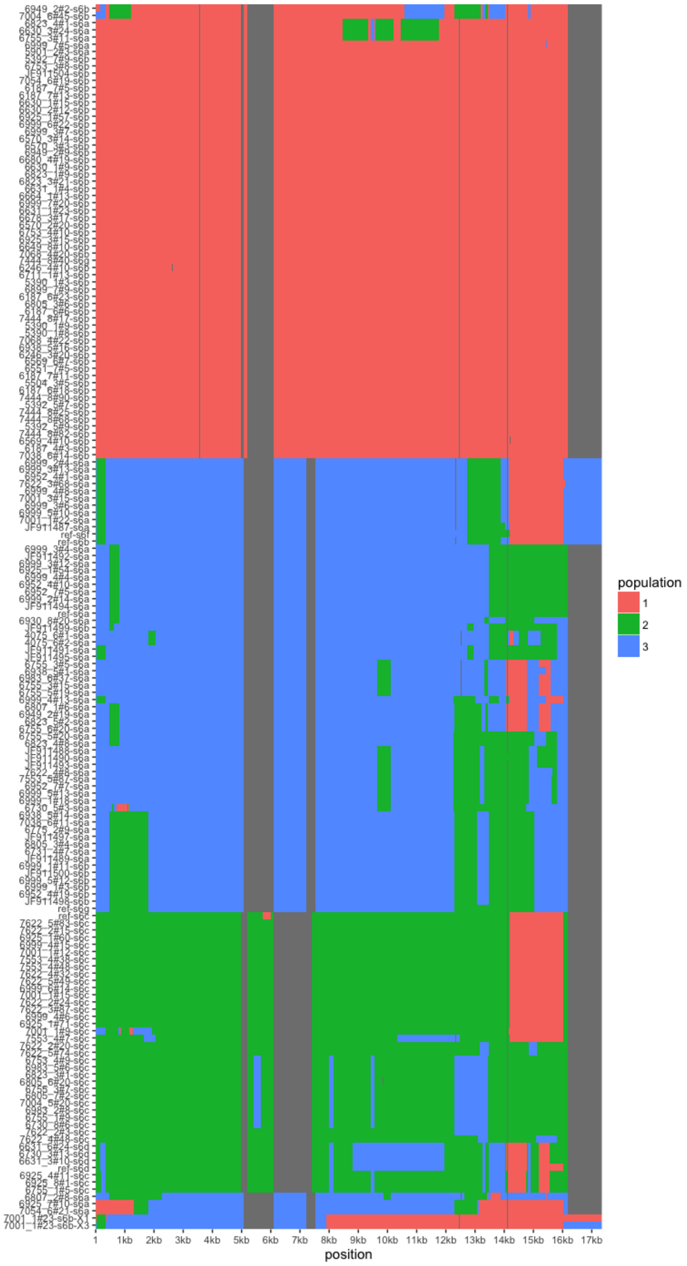

Figure S6. Population genetic structure of serogroup 6. Output of Structure is shown (see Methods). Each row corresponds to an isolate and each column to a position in the alignment. Grey colours represent alignment gaps and other colours represent the population which a given position was assigned to. The home population for each isolate was determined as the population for which at least 30% of the alignment was assigned to with at least 0.95 posterior probability; if multiple populations were found, the isolate was considered as mosaic. Recombinations here were defined as segments with a minimum 0.75 posterior and at least one site with posterior 0.95 belonging to a different population.

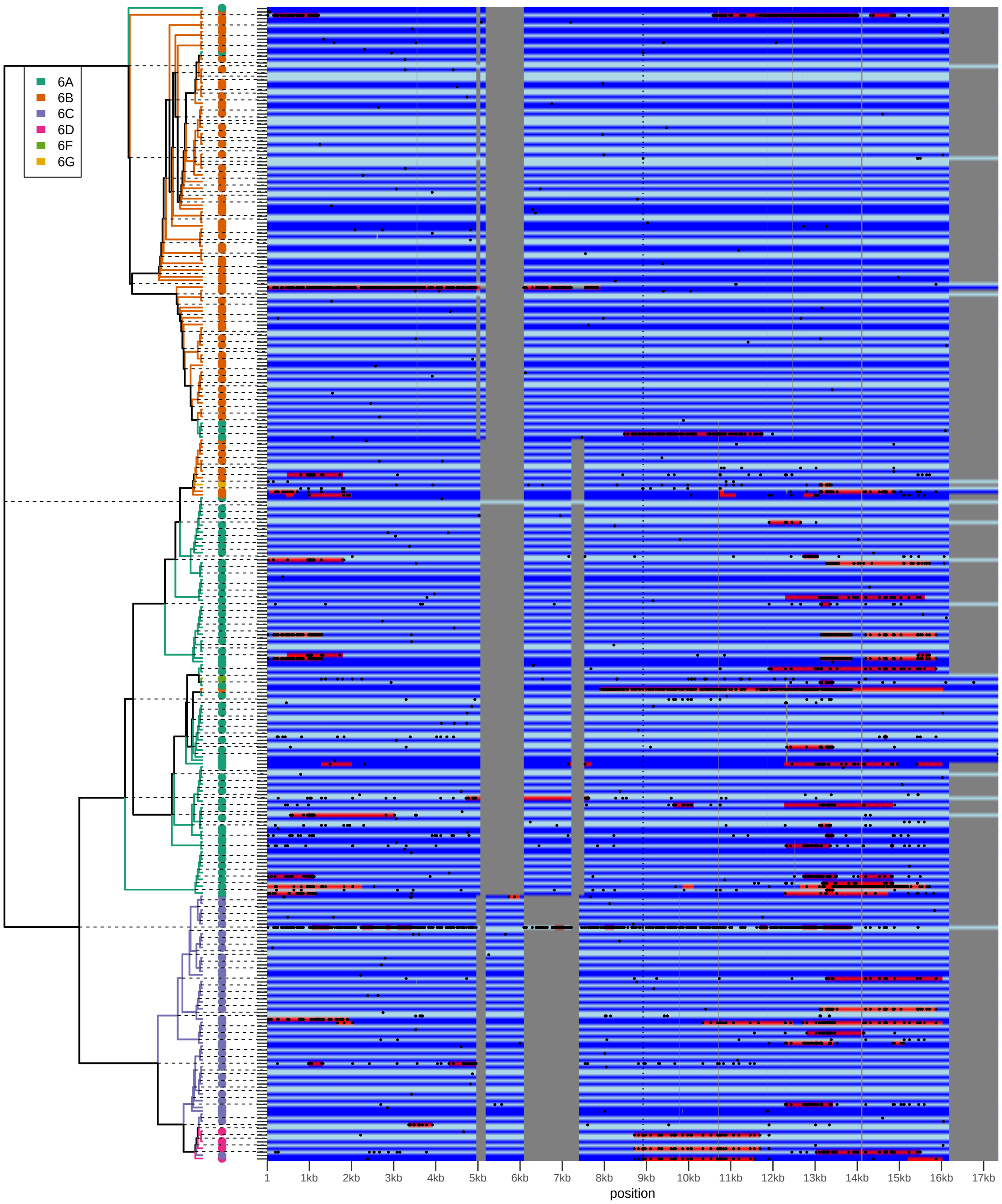

Figure S7. Evolutionary history of serogroup 6. The plot shows the inferred clonal phylogeny with recombinations assigned to the nodes of the tree, both internal and external. On the left, dendogram based on the ML phylogeny is shown, which tips coloured according to the inferred serotype. On the right, an array is shown where each row is the observed sequence (in the case of tips) or inferred sequence (in the case of internal nodes). Grey positions show alignment gaps and blue positions show all other alignment positions. Black dots show the ancestry pattern inferred by Gubbins (with two window sizes, 1kb and 10kb) and red positions show recombinations as inferred by Gubbins and Structure (or only Gubbins in the case of recombinations on internal nodes).

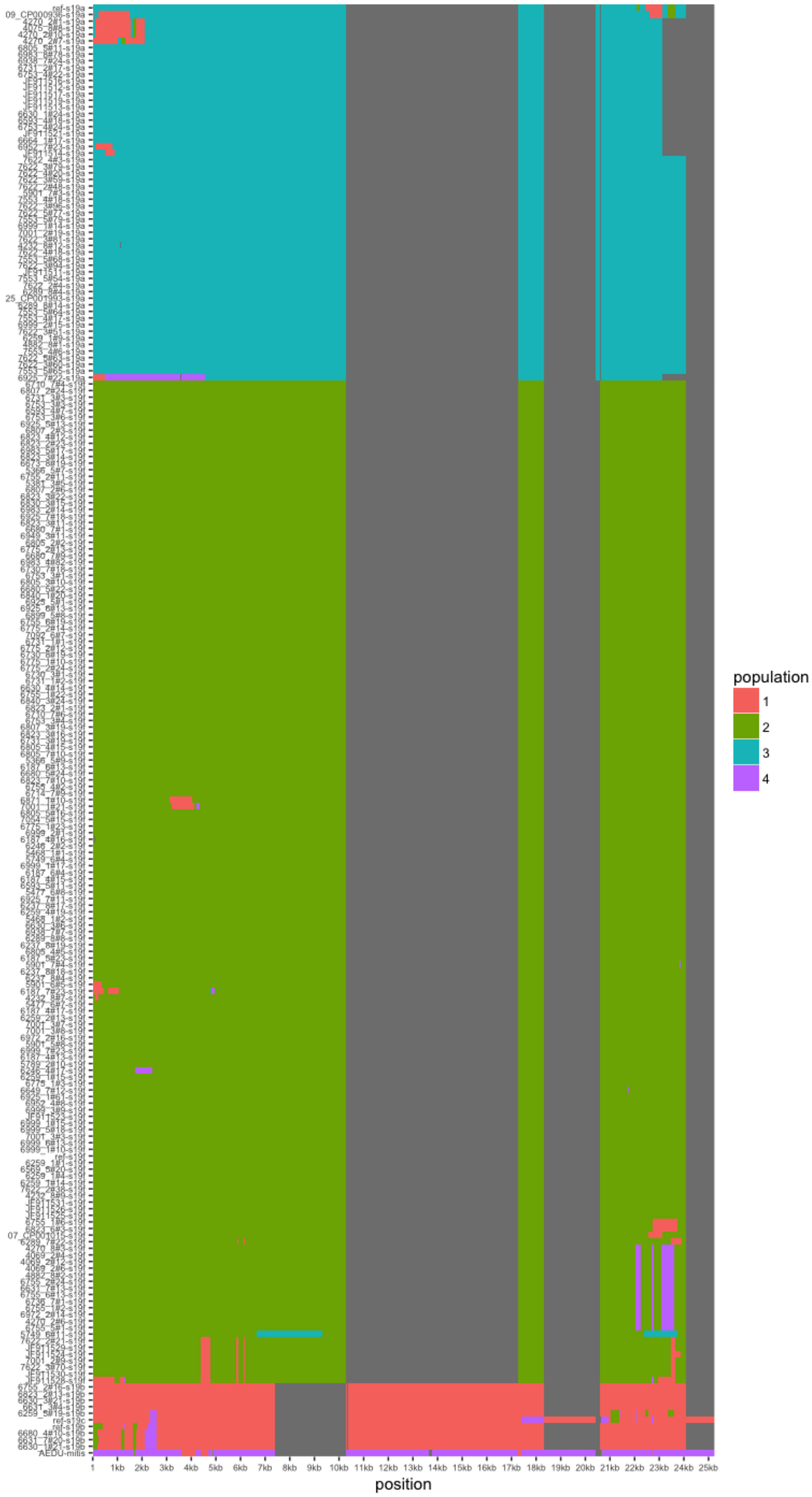

Figure S8. Population genetic structure of serogroup 19. Annotation is the same as in Figure S6.

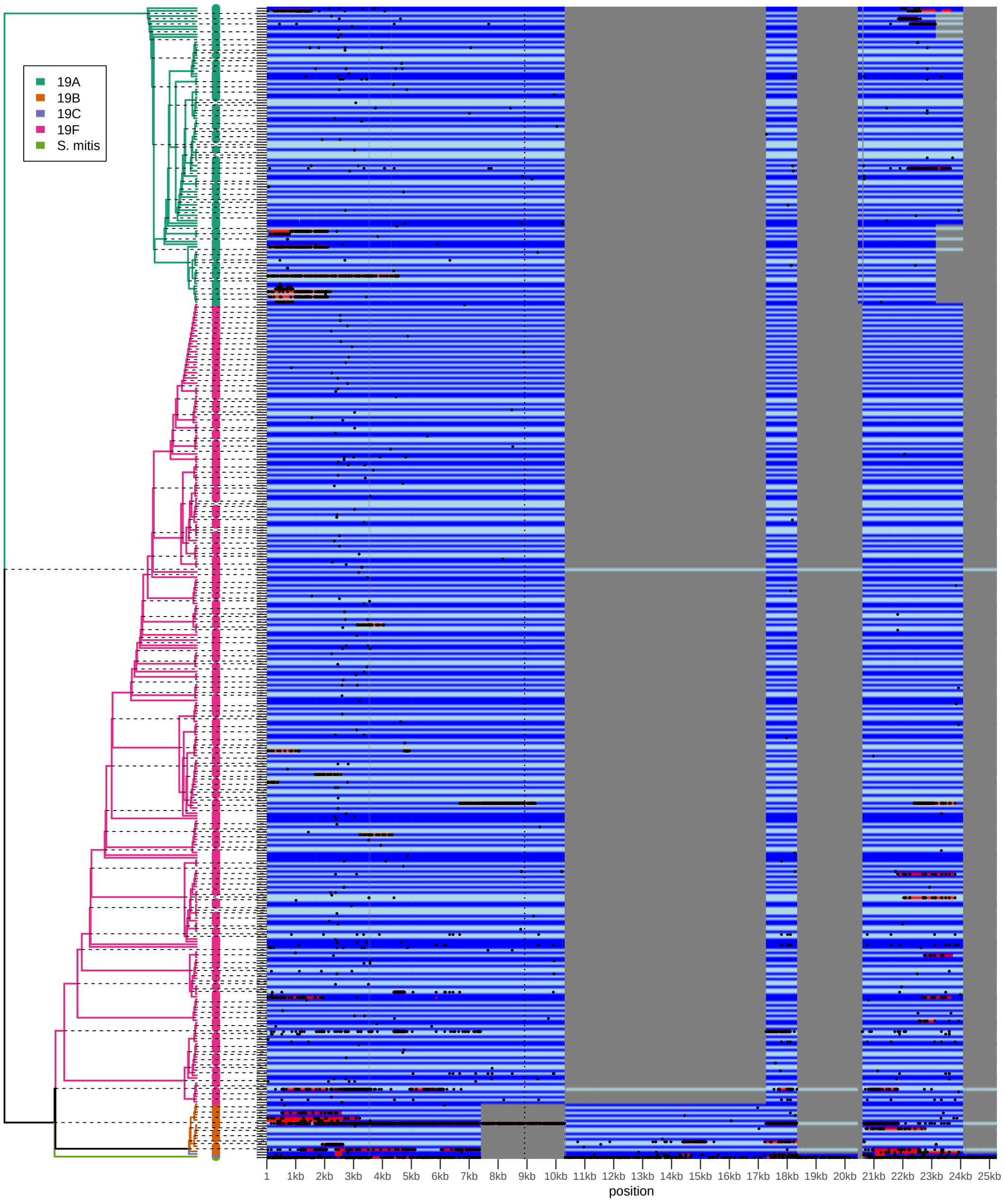

Figure S9. Evolutionary history of serogroup 19. Annotation is the same as in Figure S7.

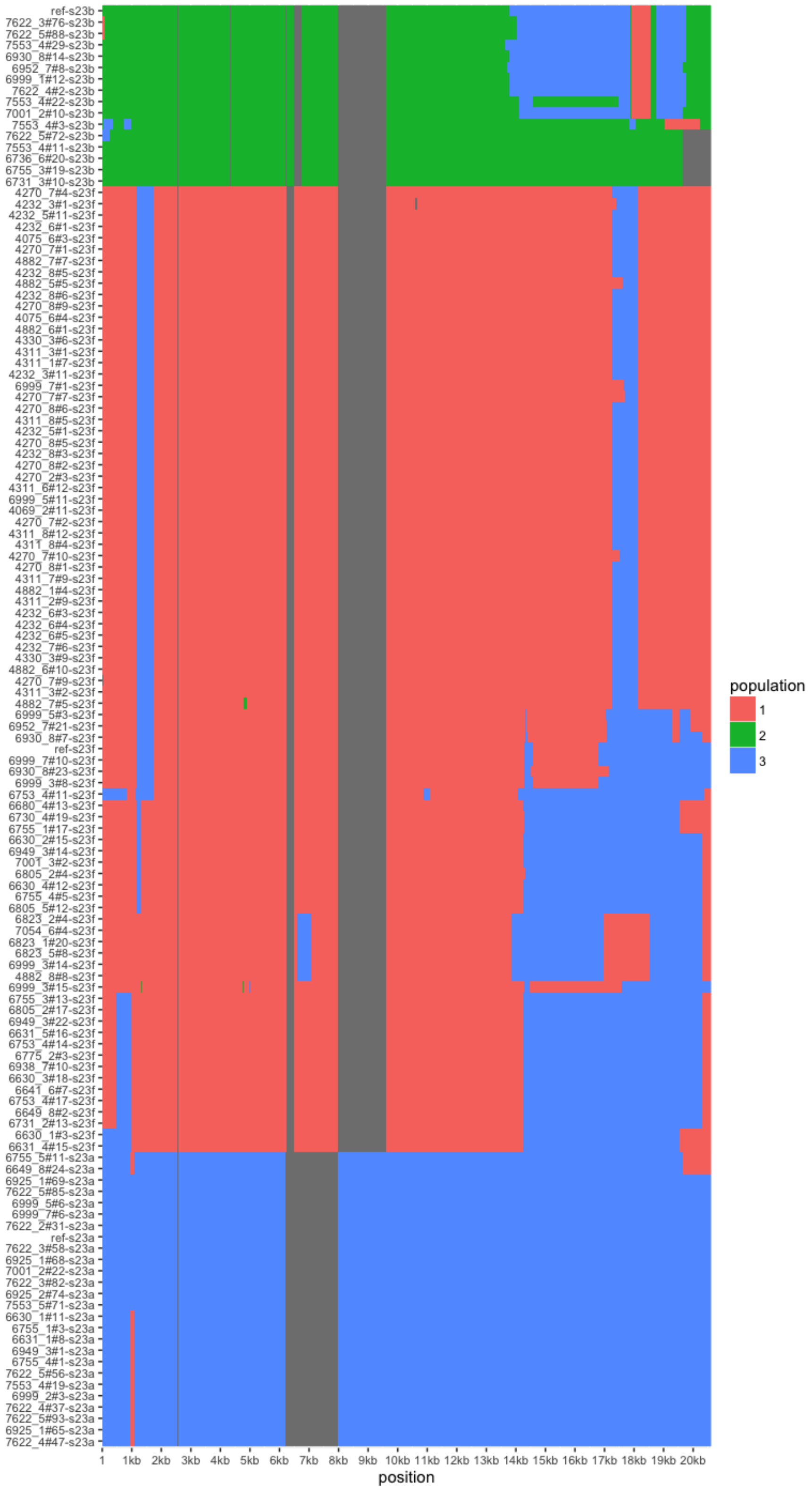

Figure S10. Population genetic structure of serogroup 23. Annotation is the same as in Figure S6.

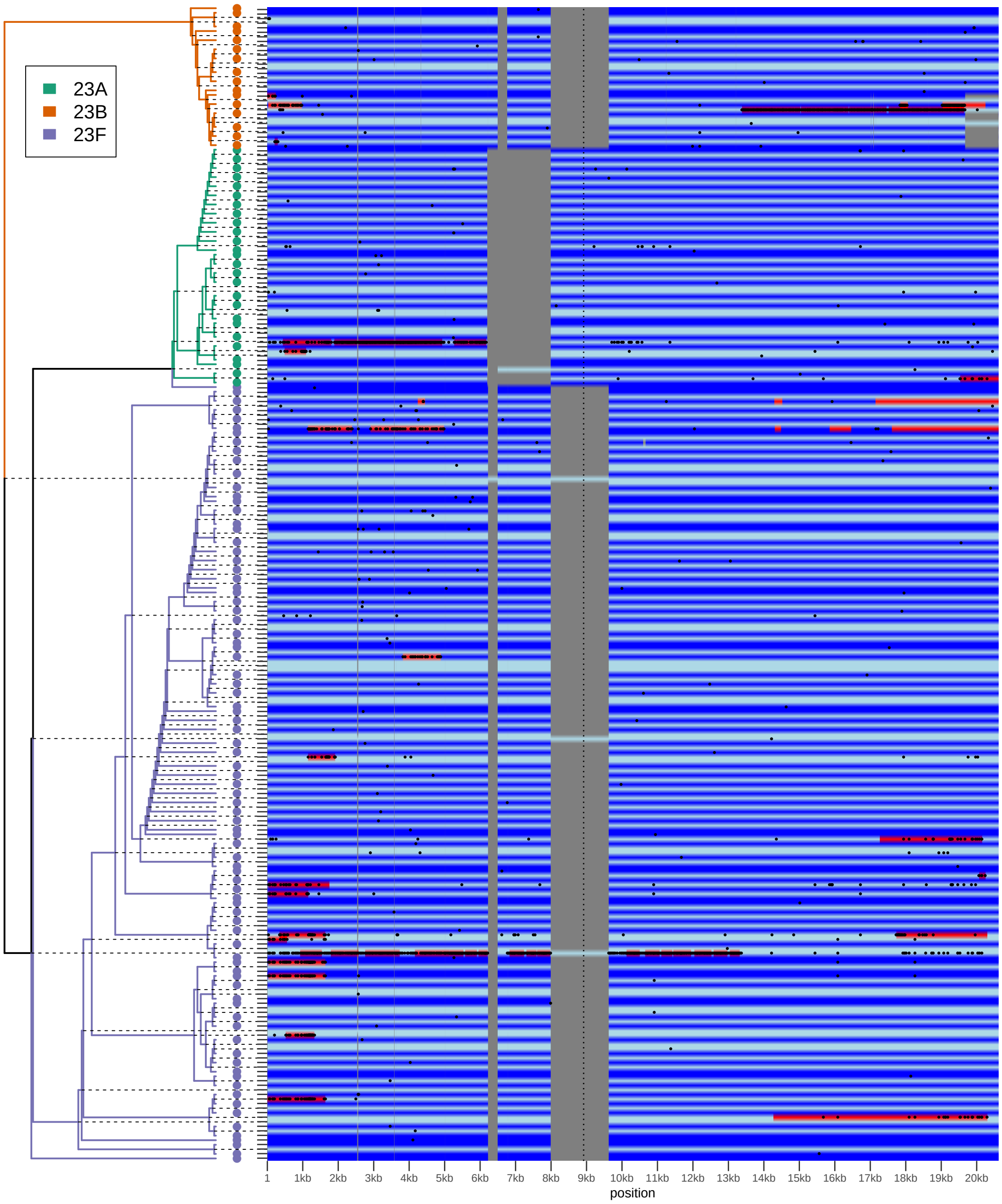

Figure S11. Evolutionary history of serogroup 23. Annotation is the same as in Figure S7.

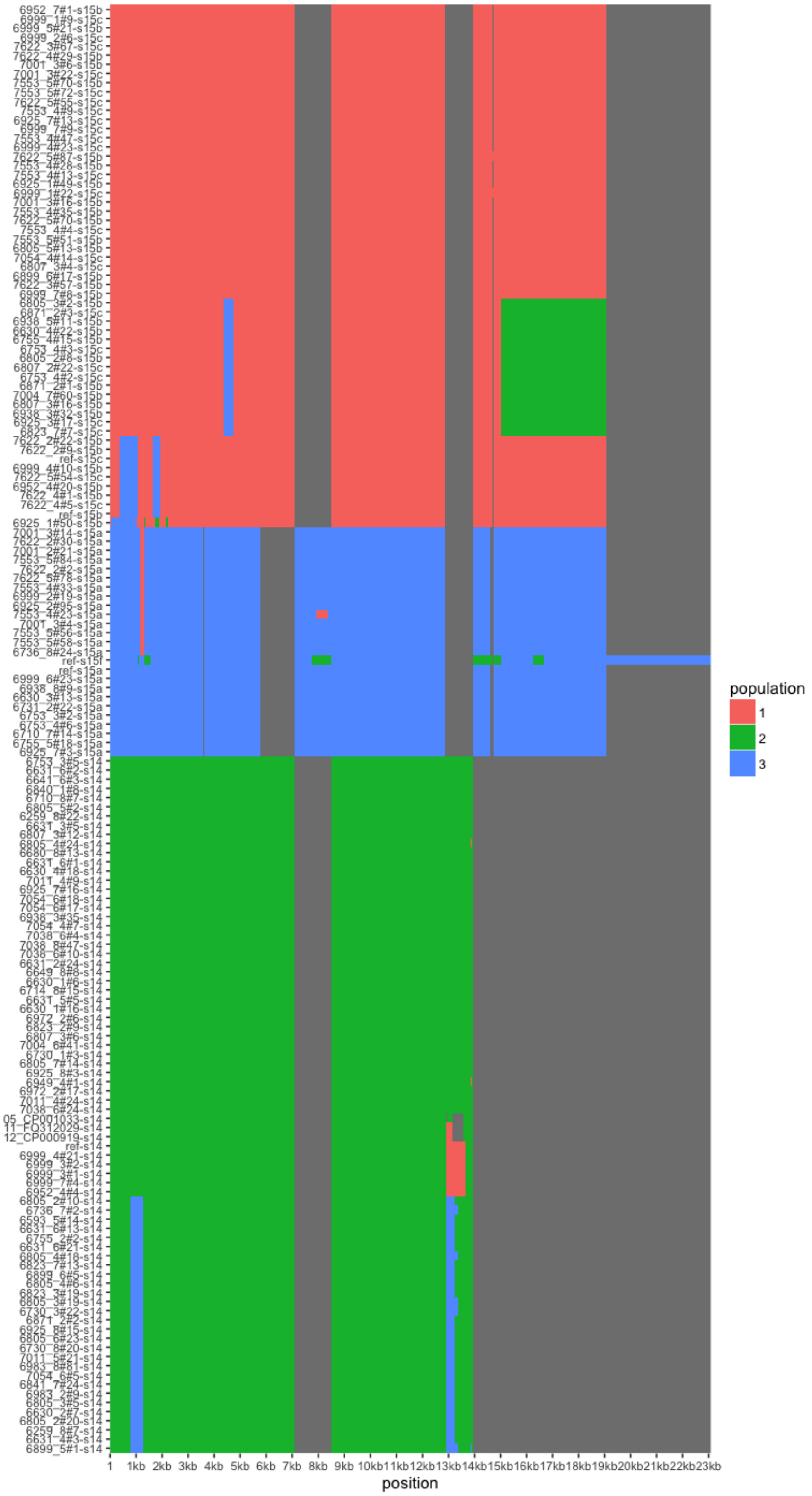

Figure S12. Population genetic structure of serogroup 14/15. Annotation is the same as in Figure S6.

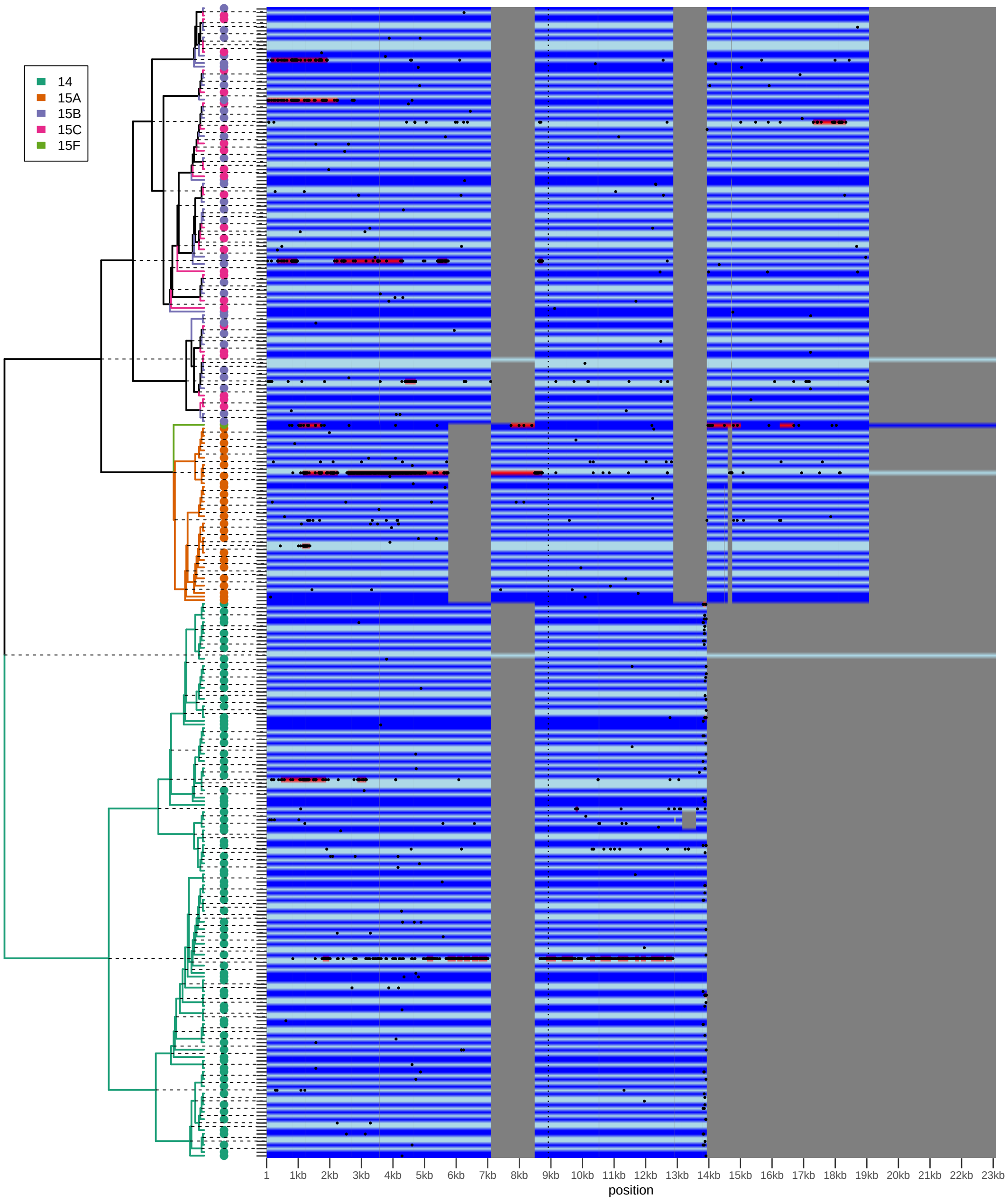

Figure S13. Evolutionary history of serogroup 14/15. Annotation is the same as in Figure S7.

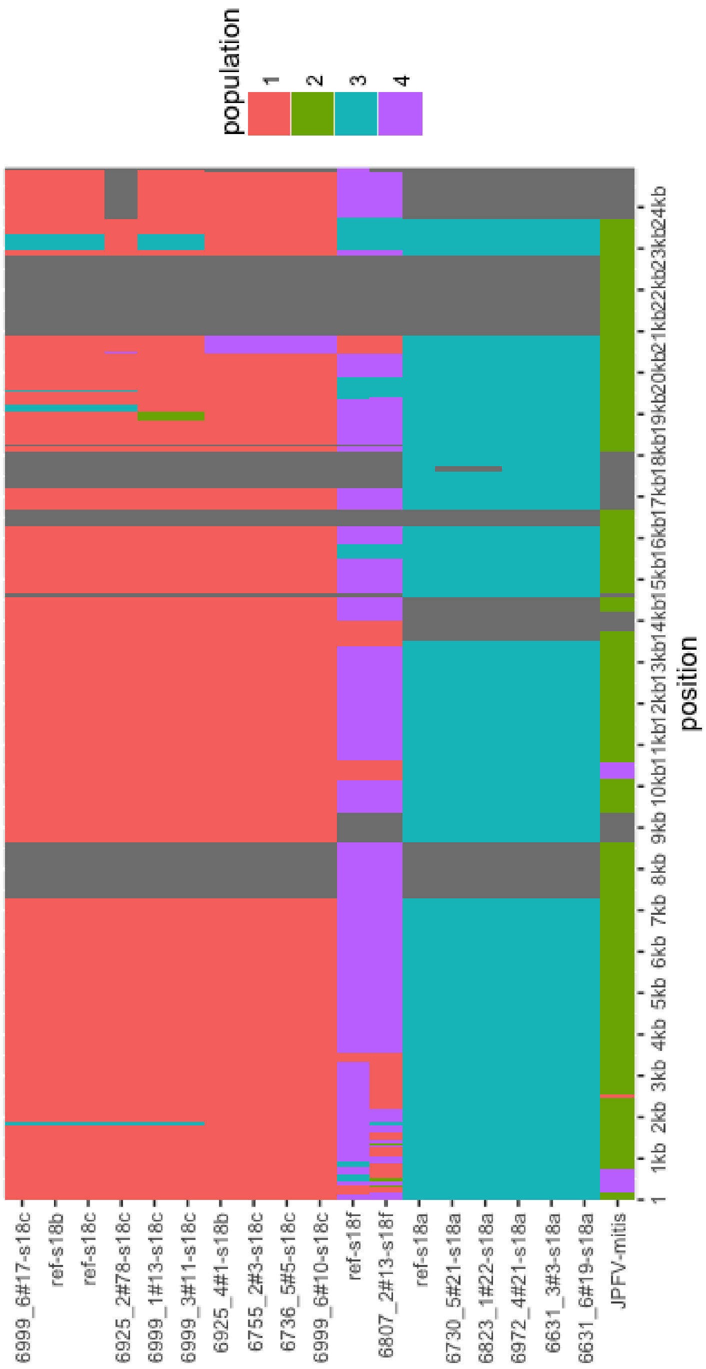

Figure S14. Population genetic structure of serogroup 18. Annotation is the same as in Figure S6.

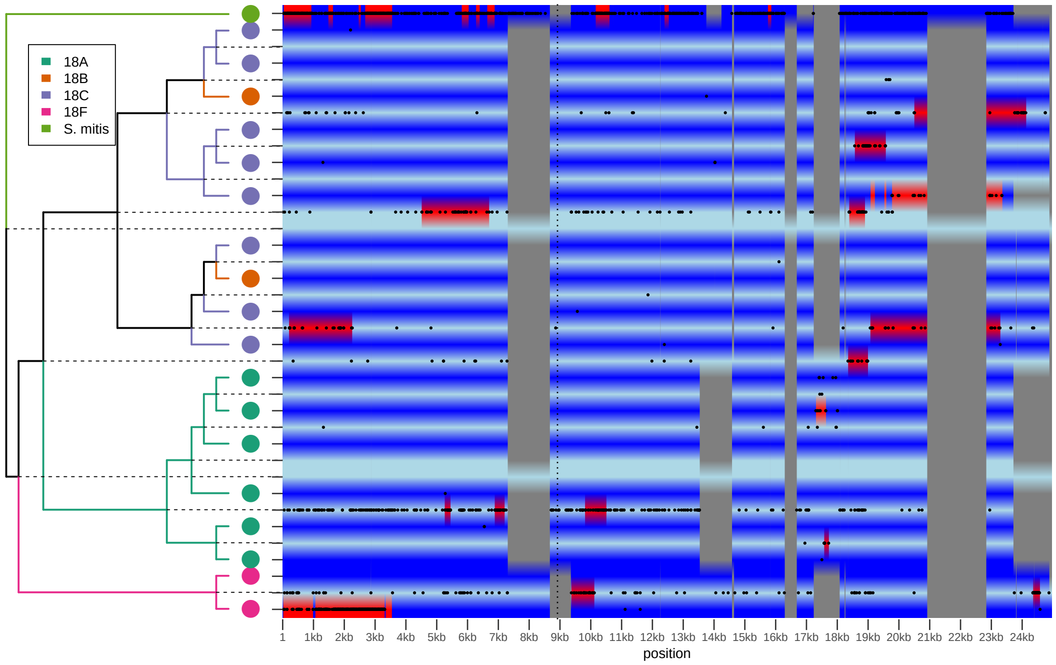

Figure S15. Evolutionary history of serogroup 18. Annotation is the same as in Figure S7.

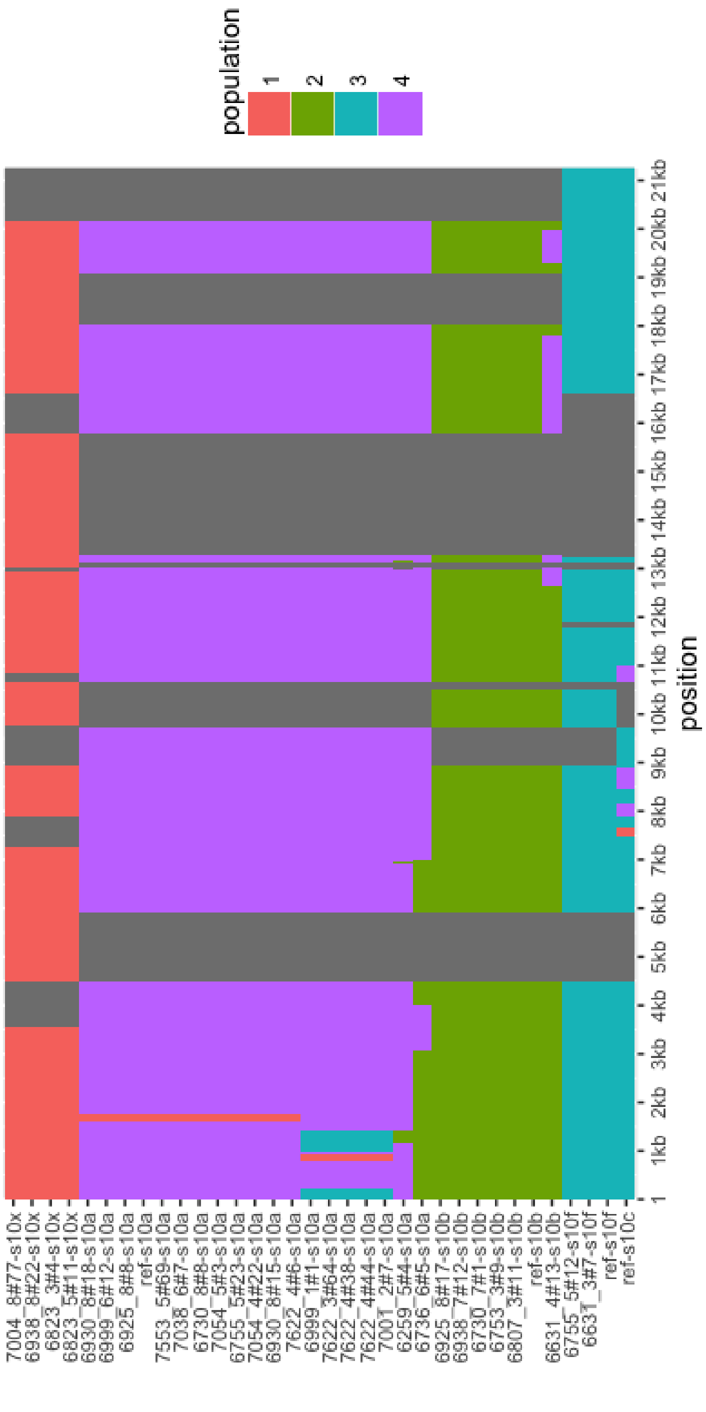

Figure S16. Population genetic structure of serogroup 10. Annotation is the same as in Figure S6.

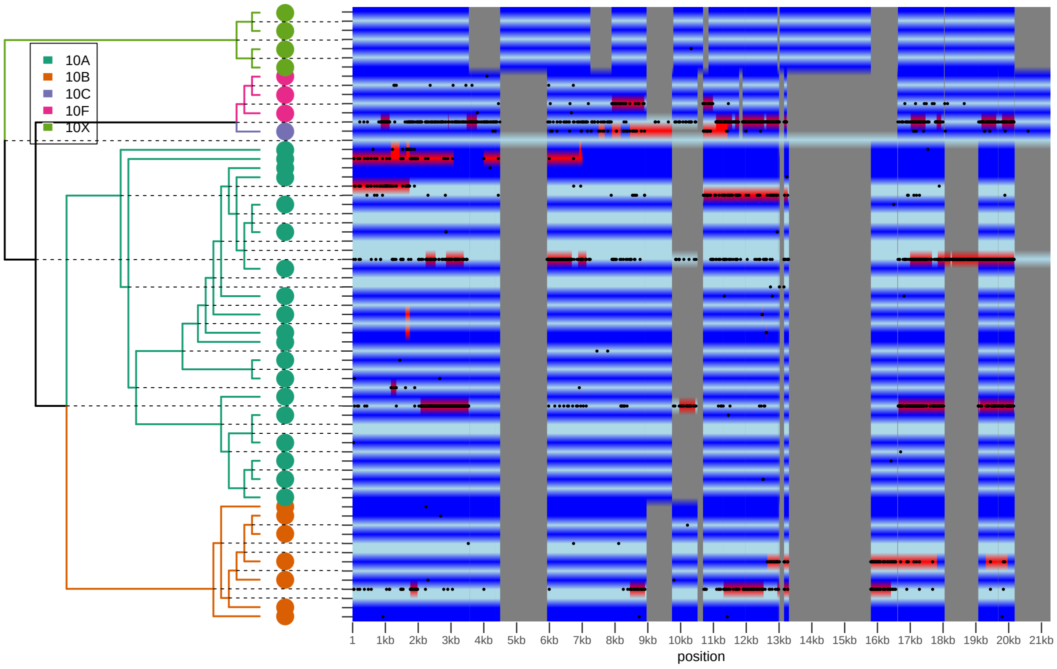

Figure S17. Evolutionary history of serogroup 10. Annotation is the same as in Figure S7.

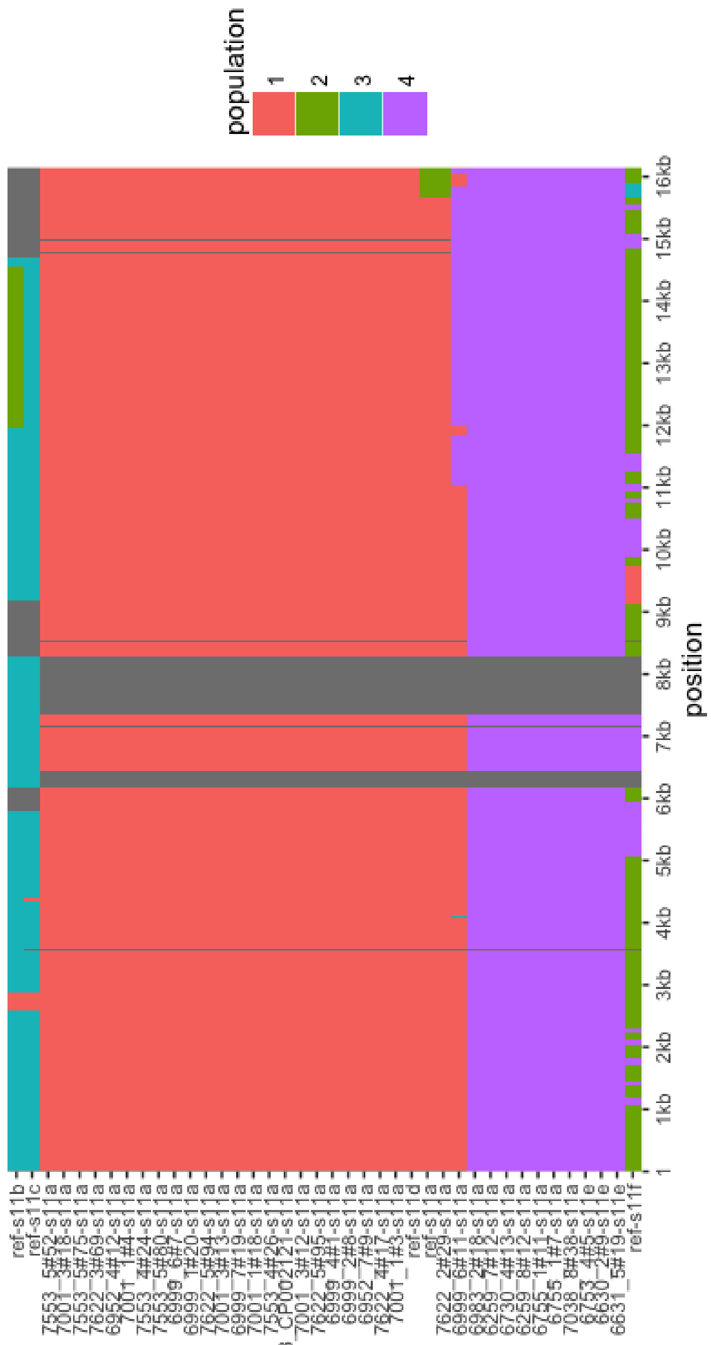

Figure S18. Population genetic structure of serogroup 11. Annotation is the same as in Figure S6.

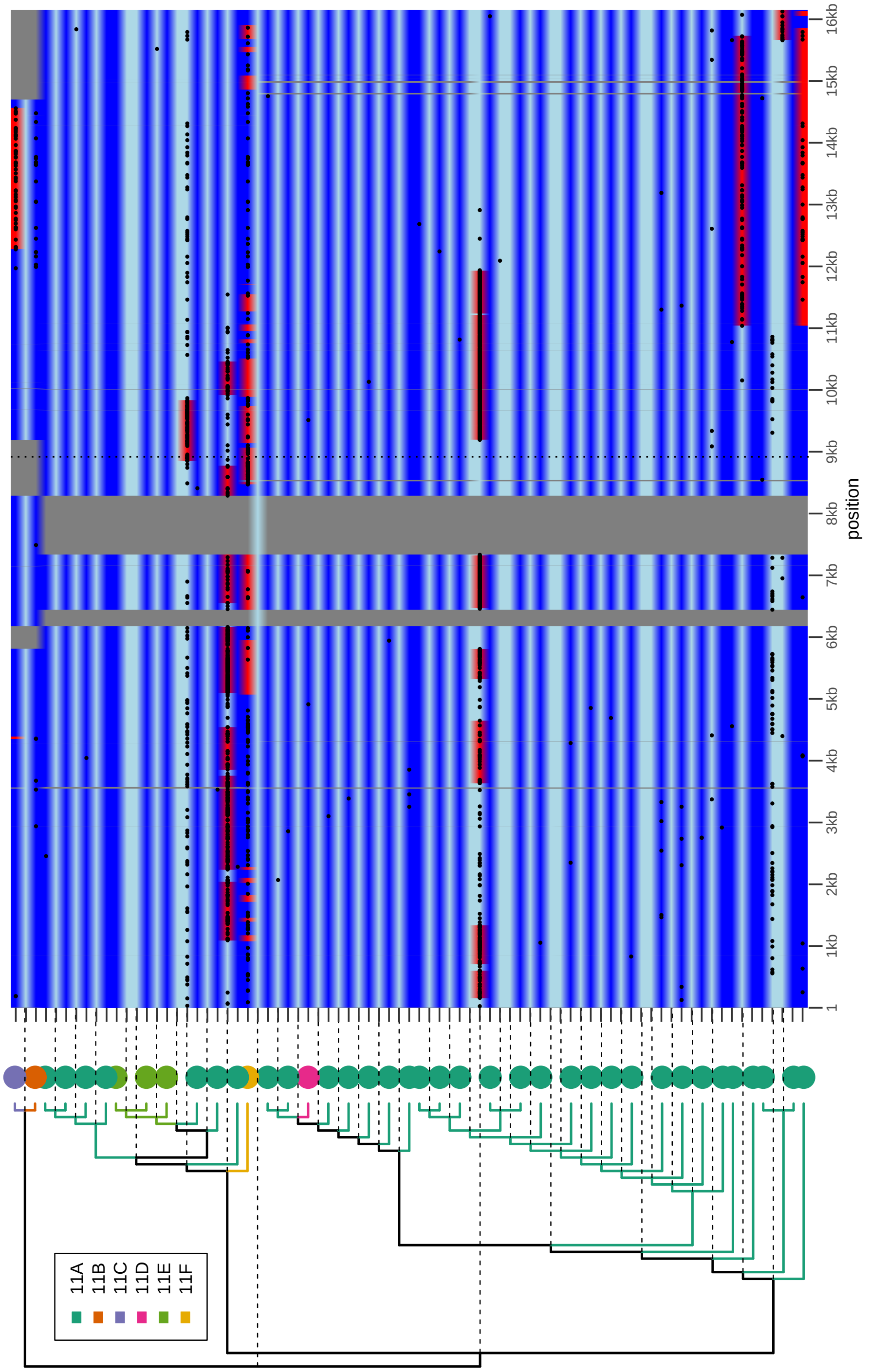

Figure S19. Evolutionary history of serogroup 11. Annotation is the same as in Figure S7.

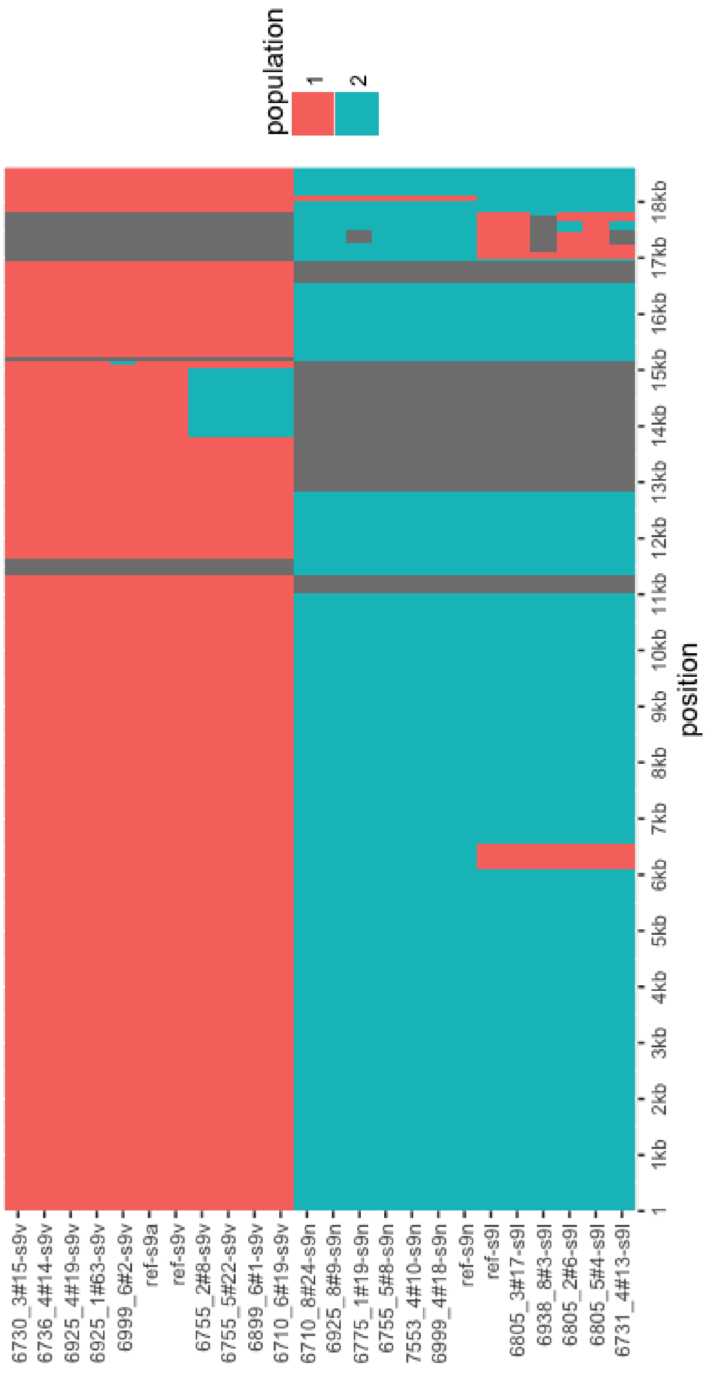

Figure S20. Population genetic structure of serogroup 9. Annotation is the same as in Figure S6.

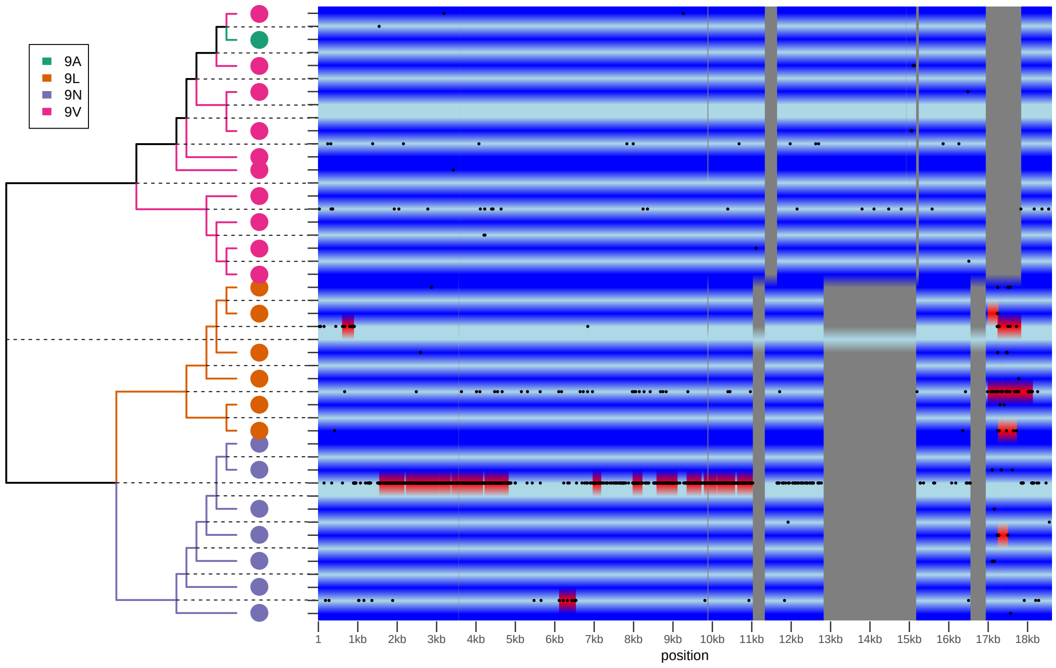

Figure S21. Evolutionary history of serogroup 9. Annotation is the same as in Figure S7.

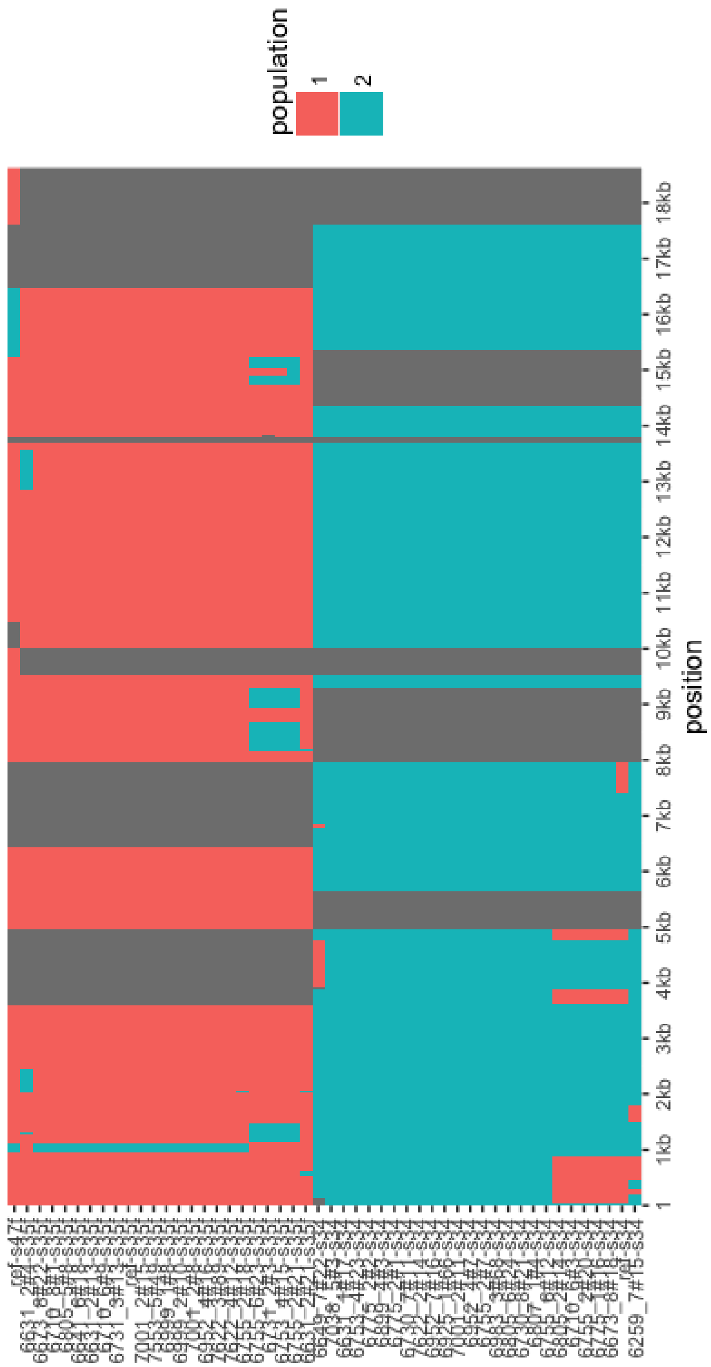

Figure S22. Population genetic structure of serogroup 34/35. Annotation is the same as in Figure S6.

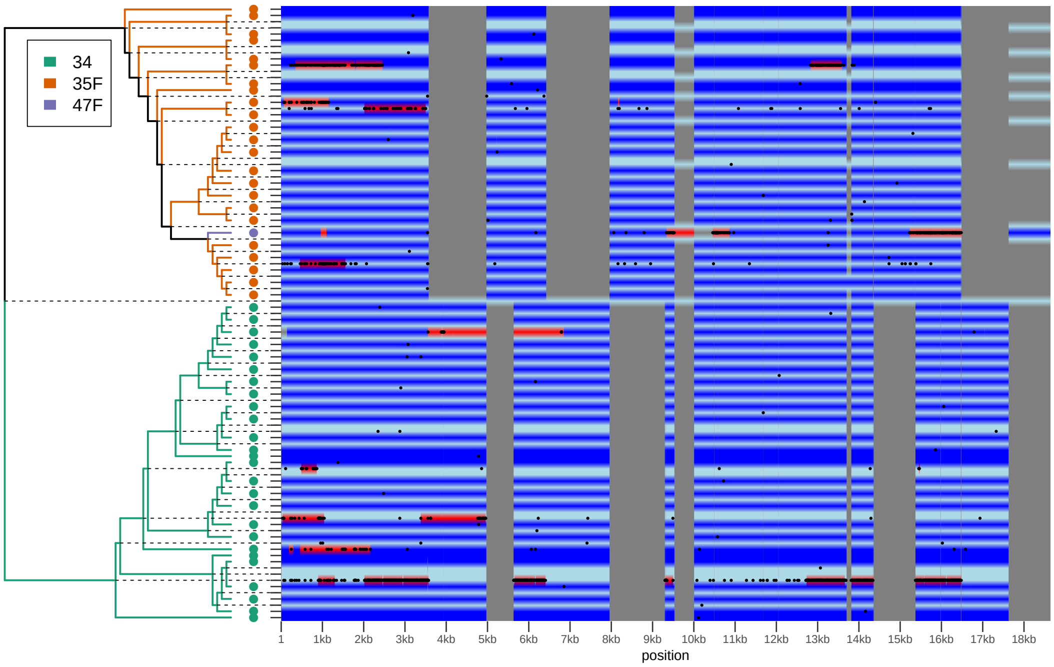

Figure S23. Evolutionary history of serogroup 34/35. Annotation is the same as in Figure S7.

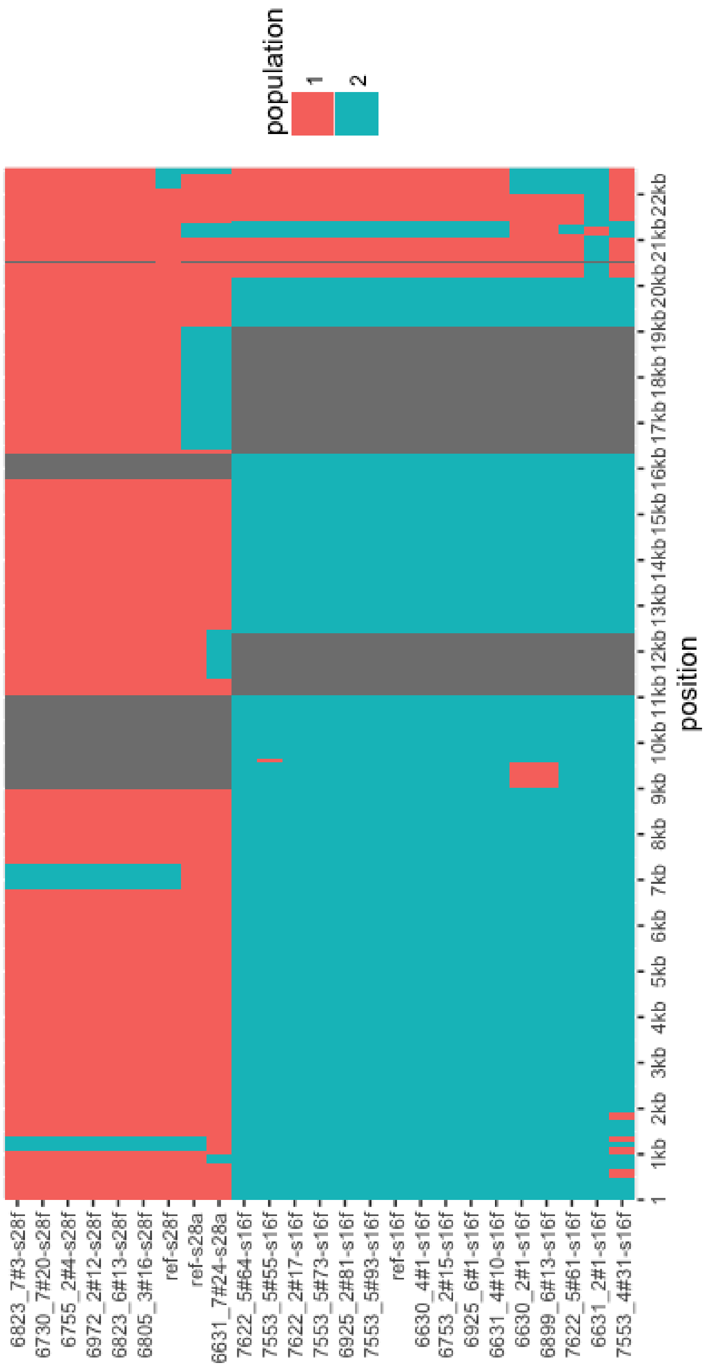

Figure S24. Population genetic structure of serogroup 16/28. Annotation is the same as in Figure S6.

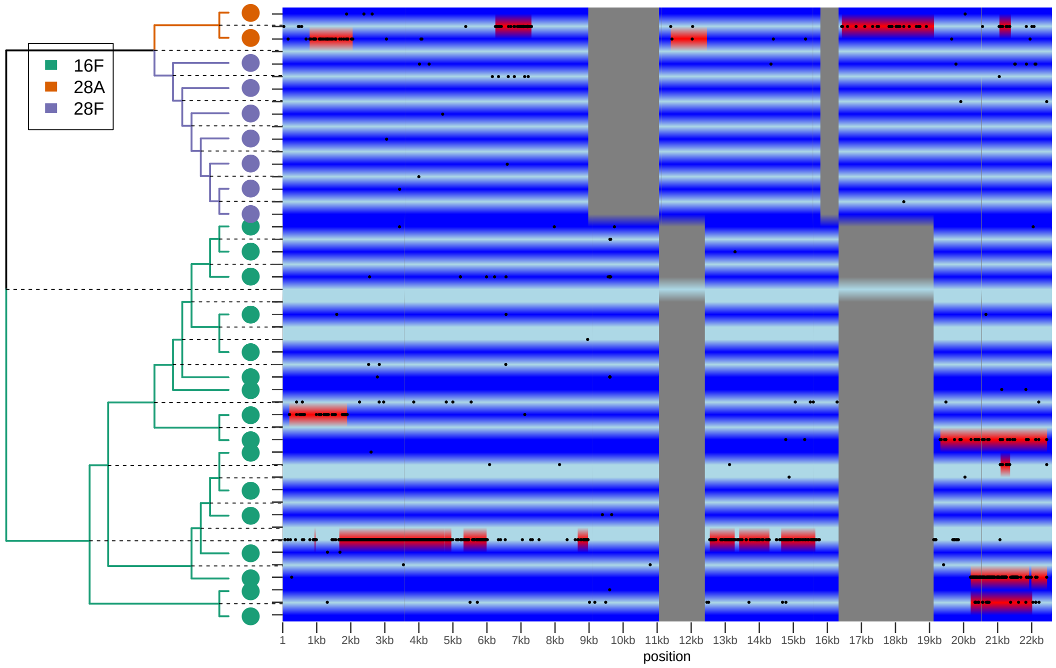

Figure S25. Evolutionary history of serogroup 16/28. Annotation is the same as in Figure S7.

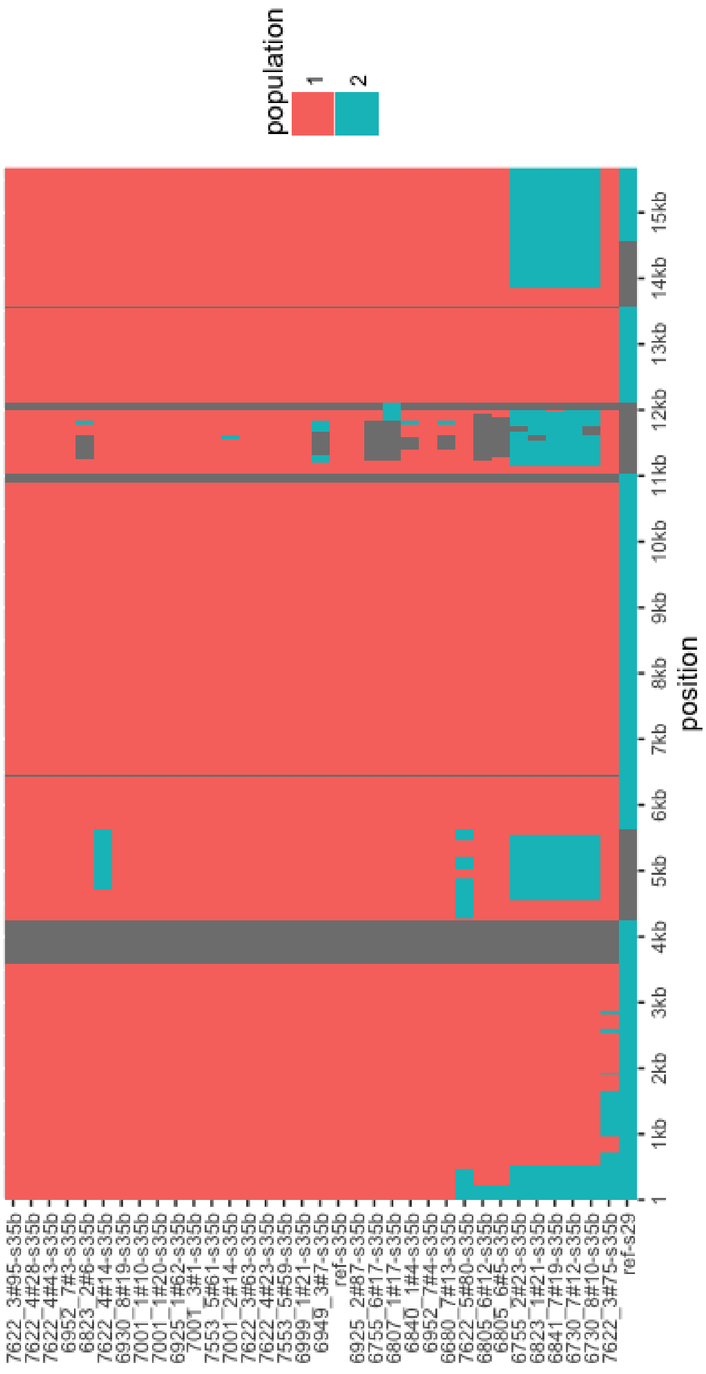

Figure S26. Population genetic structure of serogroup 29/35. Annotation is the same as in Figure S6.

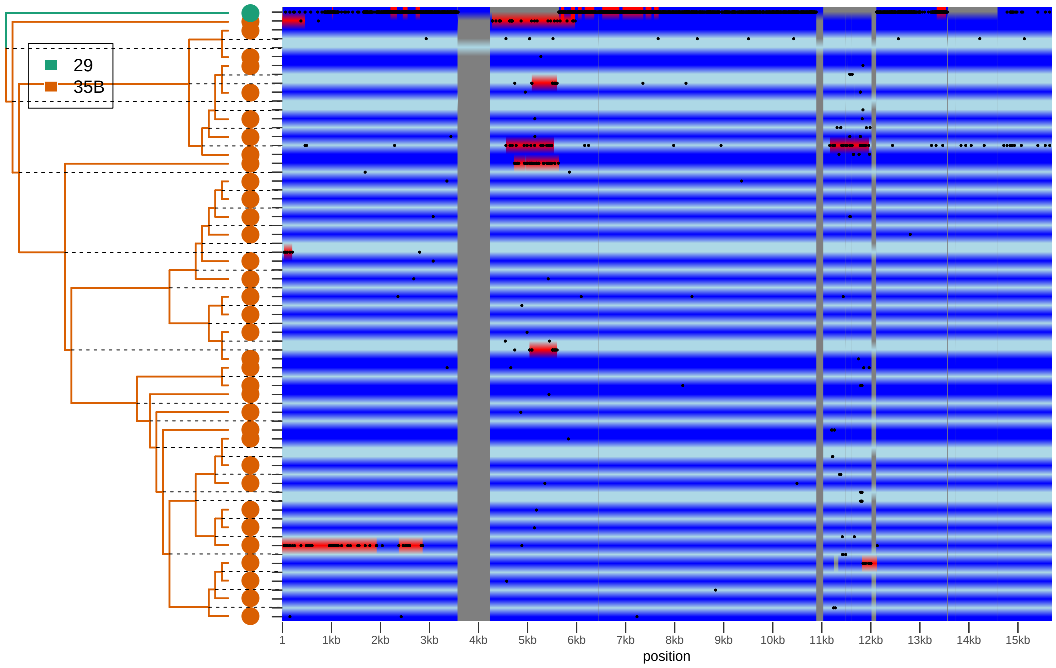

Figure S27. Evolutionary history of serogroup 29/35. Annotation is the same as in Figure S7.

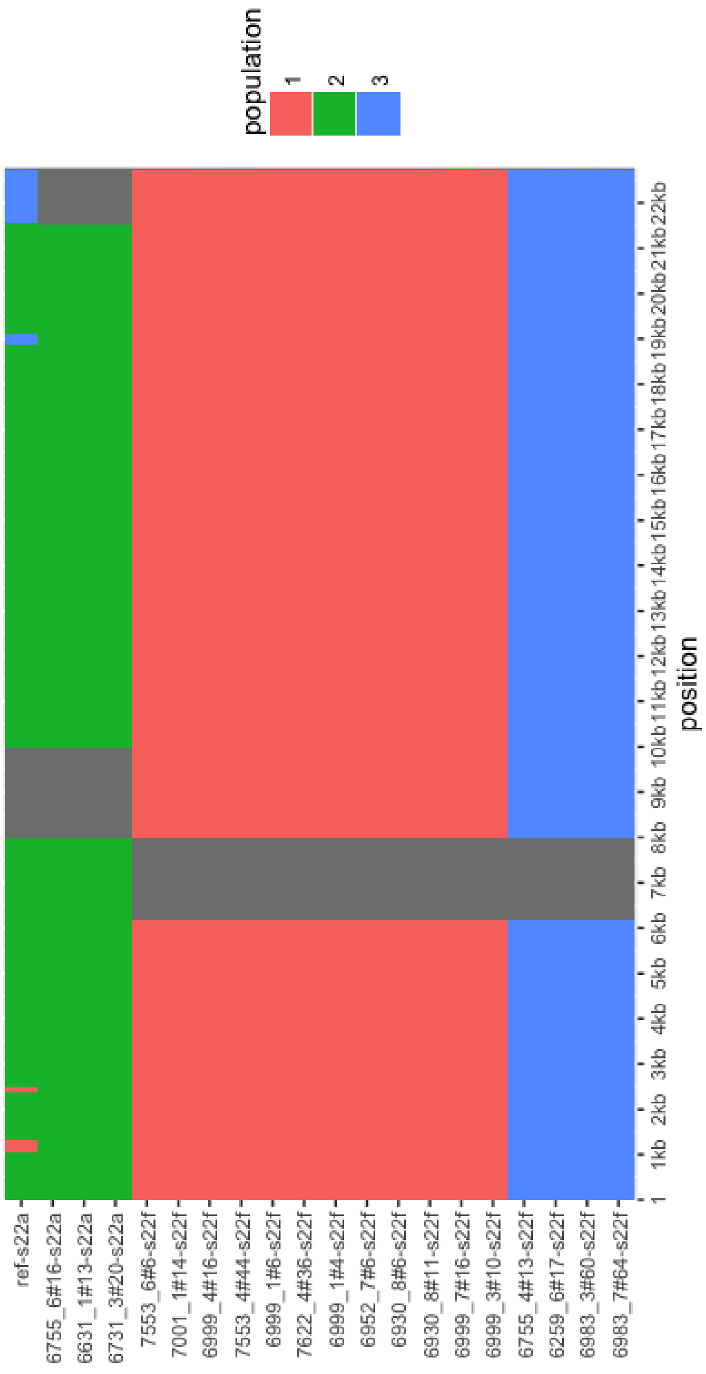

Figure S28. Population genetic structure of serogroup 22. Annotation is the same as in Figure S6.

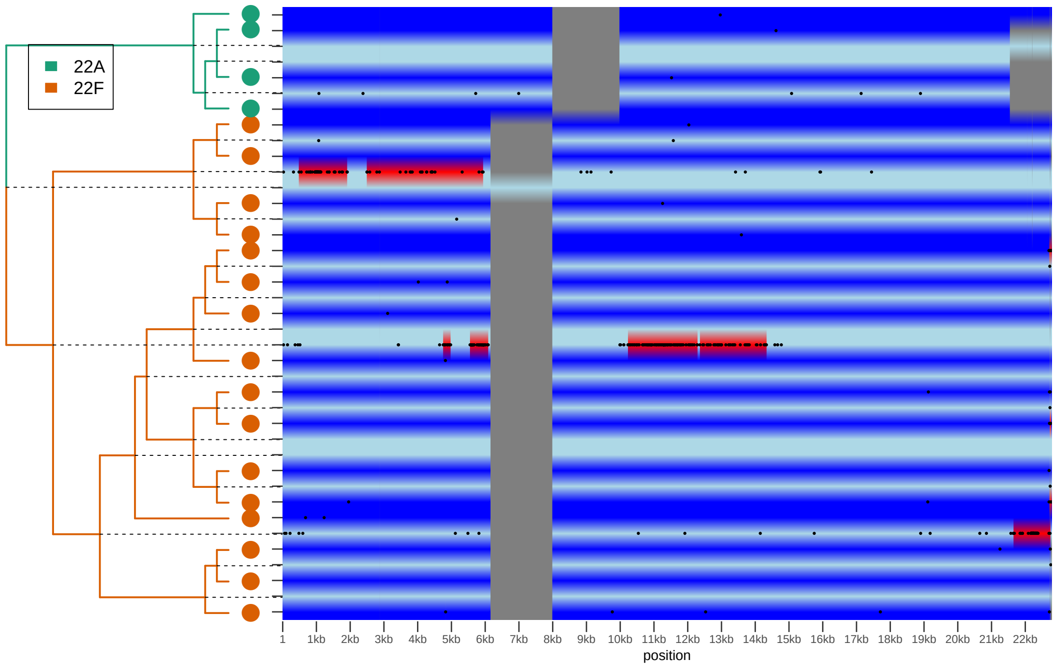

Figure S29. Evolutionary history of serogroup 22. Annotation is the same as in Figure S7.

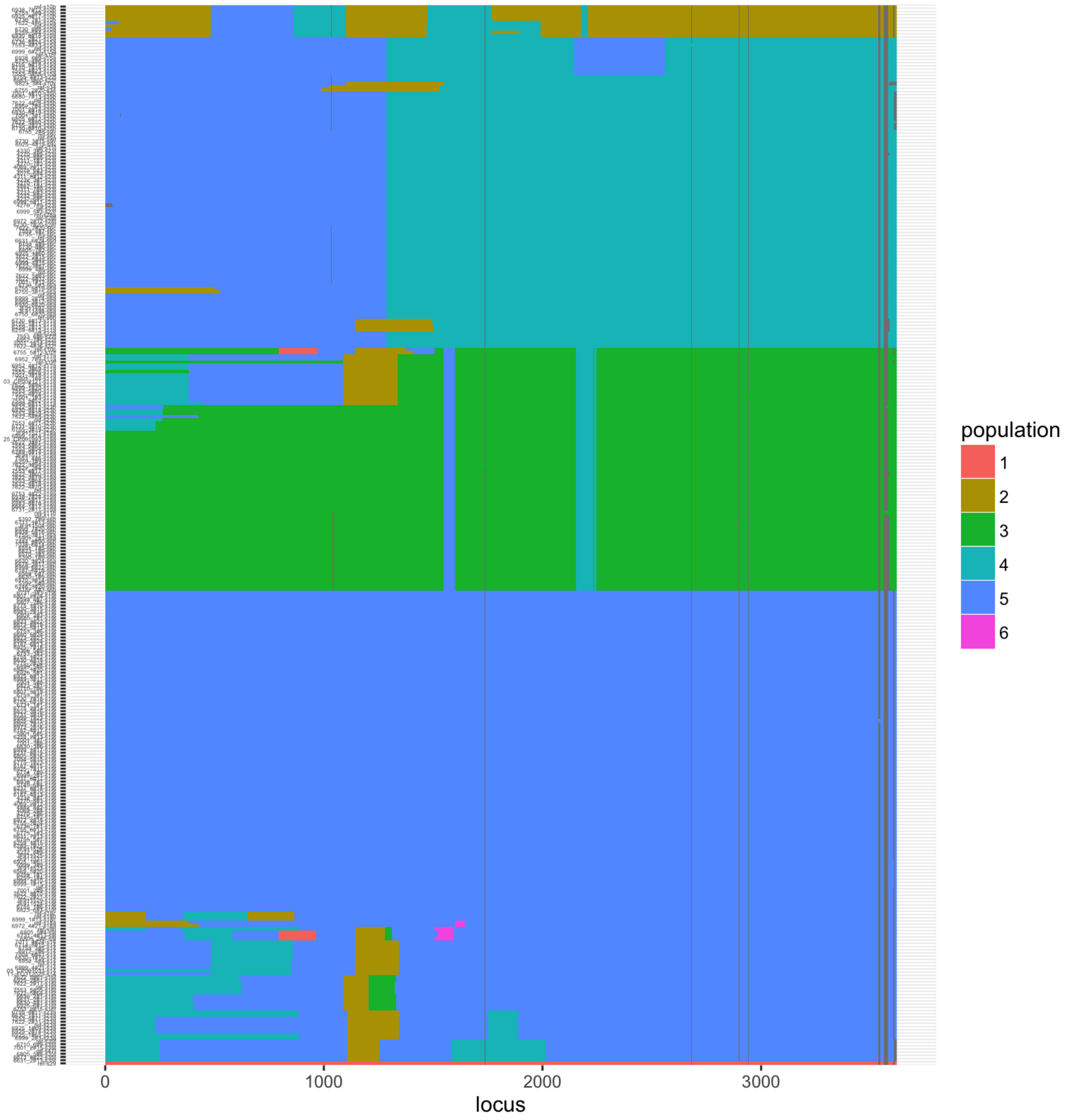

Figure S30. Population structure of the most common capsular genes: *wzg, wzh, wzd* and *wze*. Each row represents an isolate and each column represents a position in the alignment. Each entry of the matrix shows the inferred lineage using `fastGEAR` [22], with the legend showed on the right-hand side. Positions of the four genes were identified in clonal alignments of the 12 analysed serogroups; isolates with over 30% of missing data were removed. Five major lineages were identified (1-5). Isolates consisting of multiple populations (lineages) represent admixture between distant genetic lineages. Population 6 represents genetic imports from unknown source.

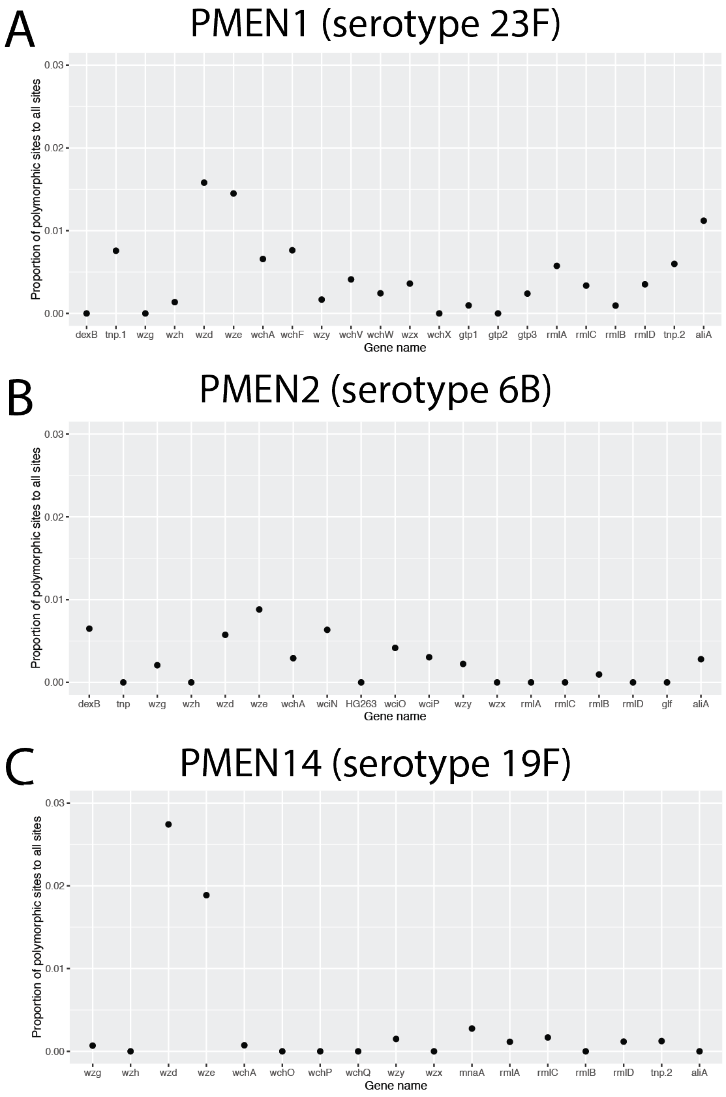

Figure S31. Distribution of diversity across capsular genes in three PMEN lineages. Capsular gene coordinates were located in the whole-genome alignment of the three PMEN lineages analysed, PMEN1 (panel A), PMEN2 (panel B) and PMEN14 (panel C). The plots show diversity for each gene, calculated by the proportion of polymorphic sites to all sites in the gene alignment. Genes with 20% of missing sites or more were disregarded.

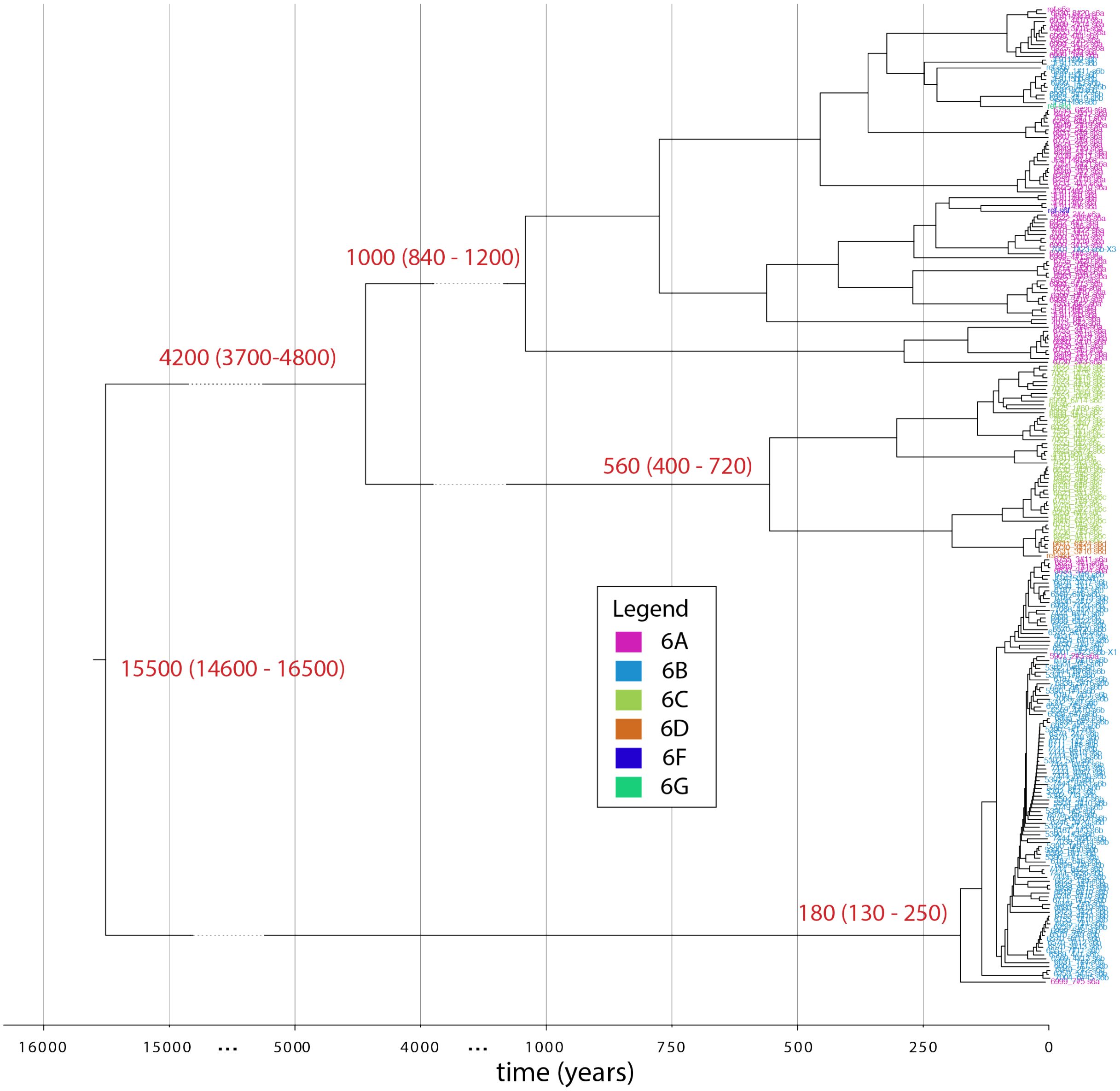

Figure S32. Timescales of evolution for serogroup 6. The clonal tree with branch lengths in years as estimated by BEAST. Tip colours correspond to different serotypes, as given in legend. Horizontal axis has been manipulated to account for large differences in branch lengths. Estimated times with 95% confidence intervals of major splits are given in red next to each ancestral node.

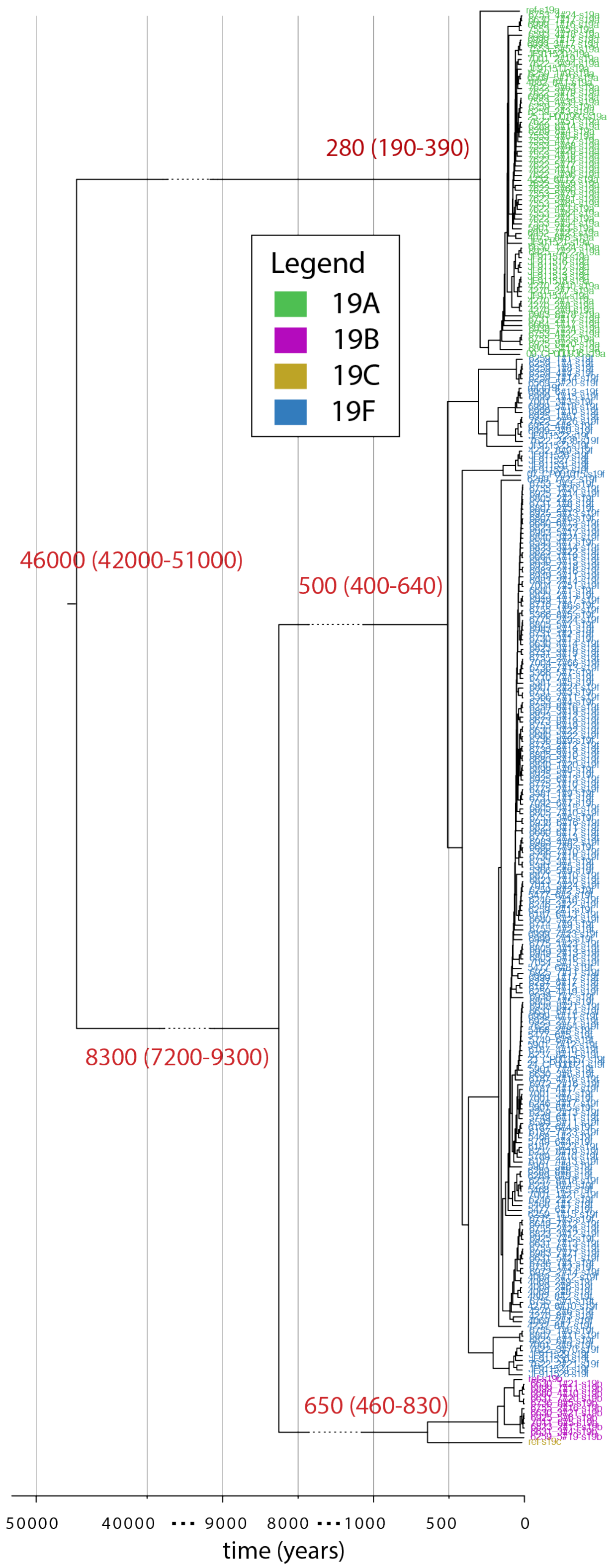

Figure S33. Timescales of evolution for serogroup 19. Annotation is the same as in figure S32.

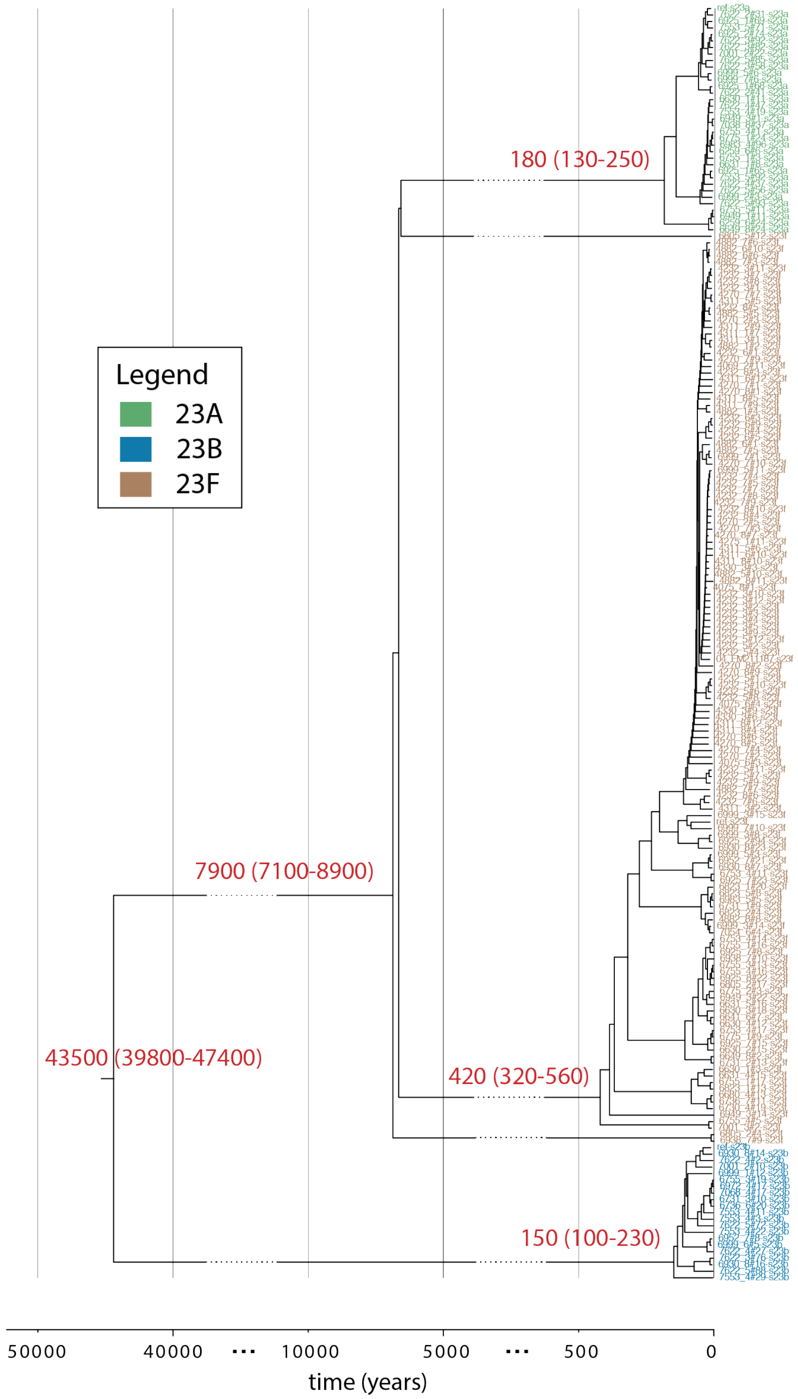

Figure S34. Timescales of evolution for serogroup 23. Annotation is the same as in figure S32.

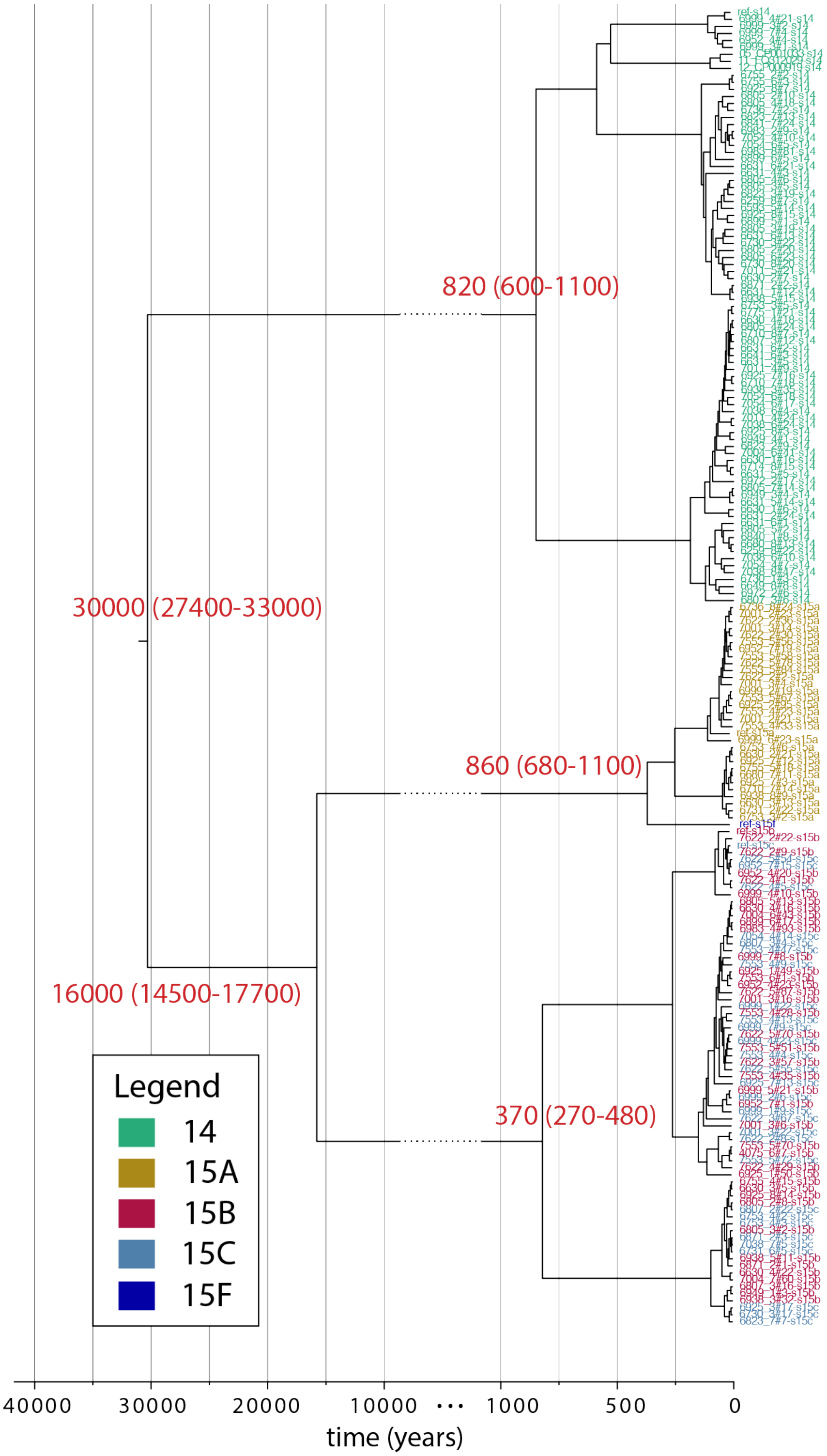

Figure S35. Timescales of evolution for serogroup 14/15. Annotation is the same as in figure S32.

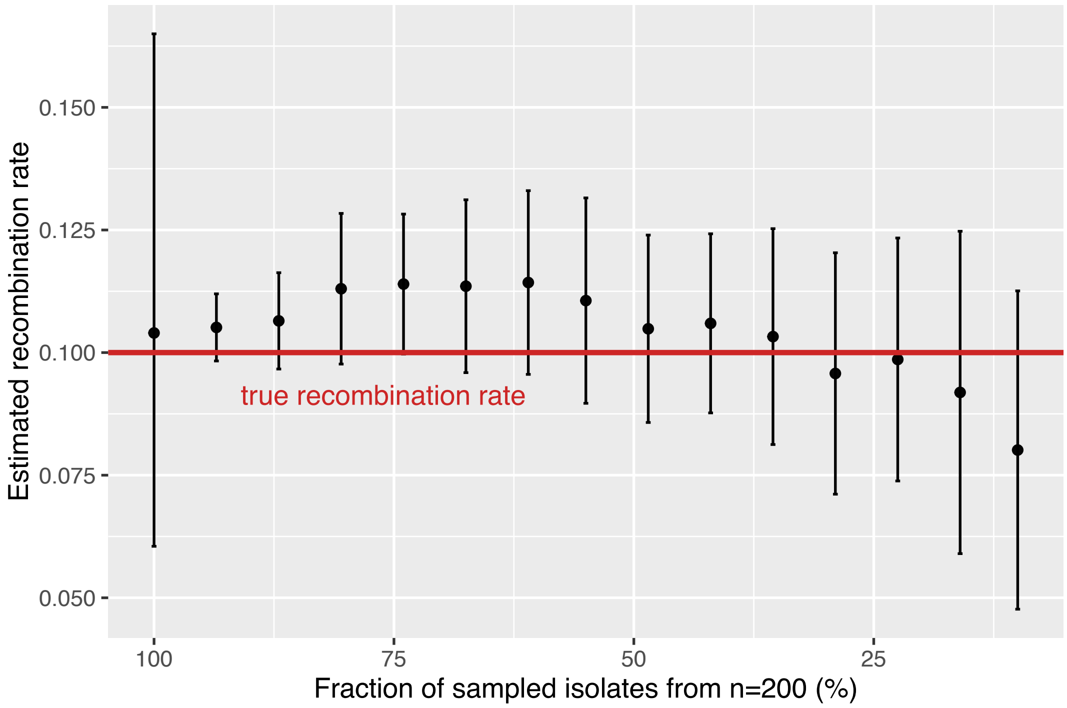

Figure S36. Estimates of recombination versus sampling. Simulation of how sampling affects the estimate of the recombination rate. Two clonal populations with 200 mebmers within each population were generated with the average within-population distance of 70 SNPs and between-population distance of 16,000 SNPs using `fastSimCoal` [23]. The alignment of length 20kb was then produced using `seq-gen` [24]. We then simulated bacterial transformation in population 1 with population 2 acting as a donor, and with the number of recombinations at each branch being Poisson distributed with rate $\lambda = 0.1$ and mean length $\Sigma = 500$. A randomly picked subsample was analysed with Gubbins and the recombination rate was estimated, which was performed 30 times indenepdently. The estimate for the full sample shows the 95% confidence intervals; the remaining estimates show the 95% quantile of the estimated rates in 30 runs for each sample size. Red line shows the true, simulated recombination rate.

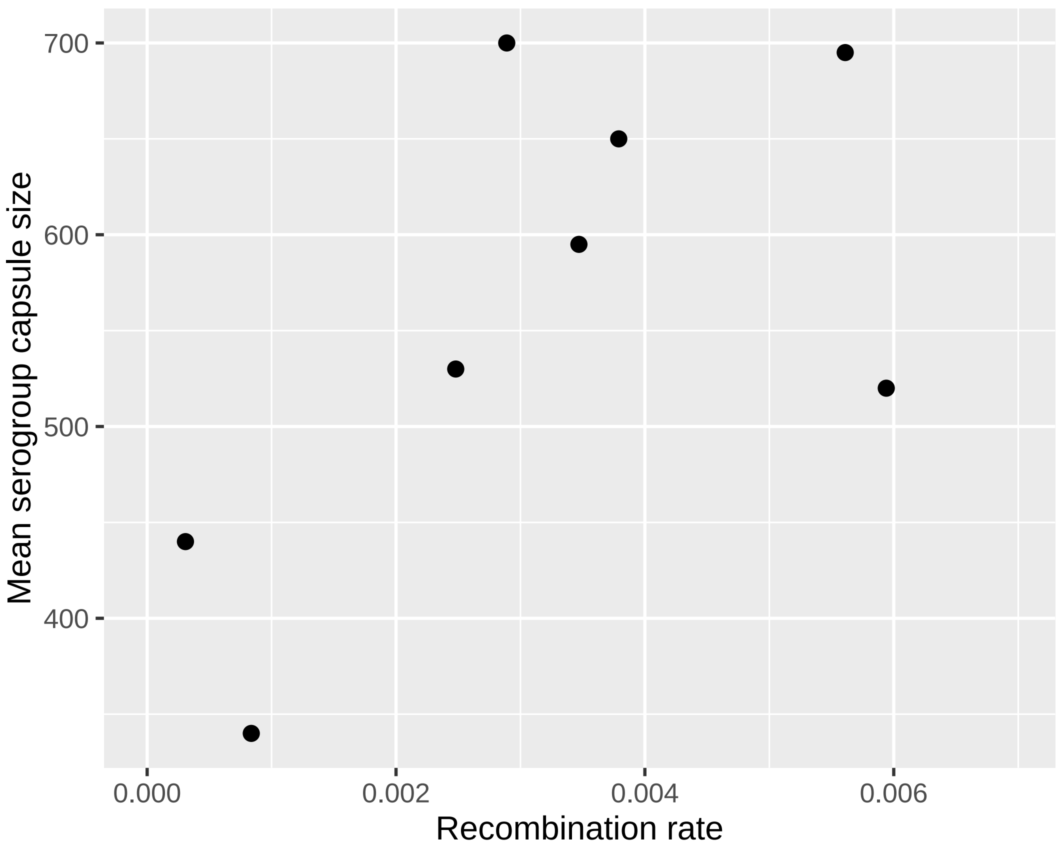

Figure S37. Estimated recombination rate versus capsule size. The capsule size (degree of encapsulation) data are reported as estimated by FITC-Dextran exclusion in Weinberger et al. [25]. The mean serogroup capsule size was estimated for the serogroups which included serotypes for which the estimates were available: serogroup 6, 9, 11, 14/15, 18, 19, 23 and 29/35. The mean recombination rates were calculated using estimates in Figure 4.

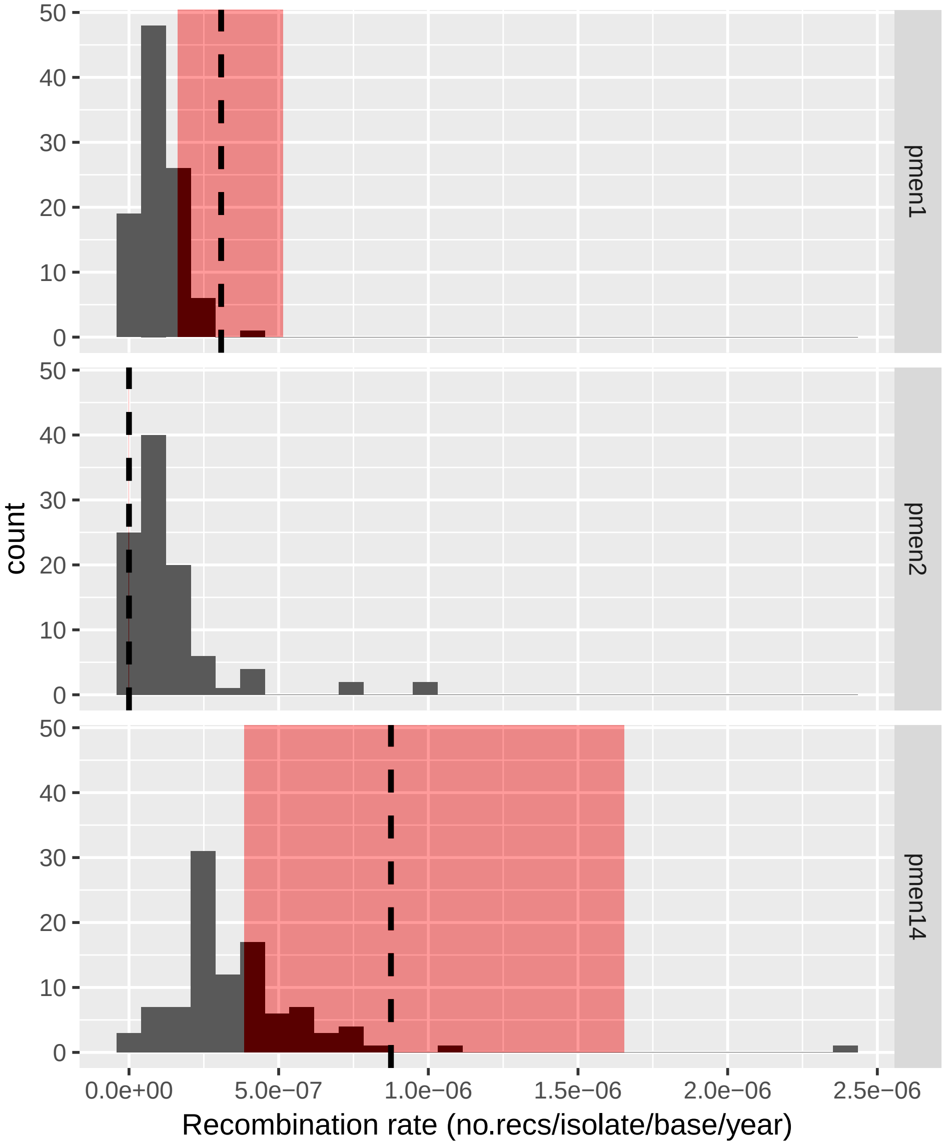

Figure S38. Distribution of recombination rate in three PMEN lineages. Panel shows the distribution of recombination rates in randomly sampled regions of length 20kb. The locations were randomly drawn from the genome, and the rate of recombinations affecting this region was calculated as described in the main text. The procedure was repeated 100 times. The shaded red areas show the estimated rate of recombinations affecting the *cps* locus. The area is missing from the PMEN2 because no *cps* recombinations were observed.

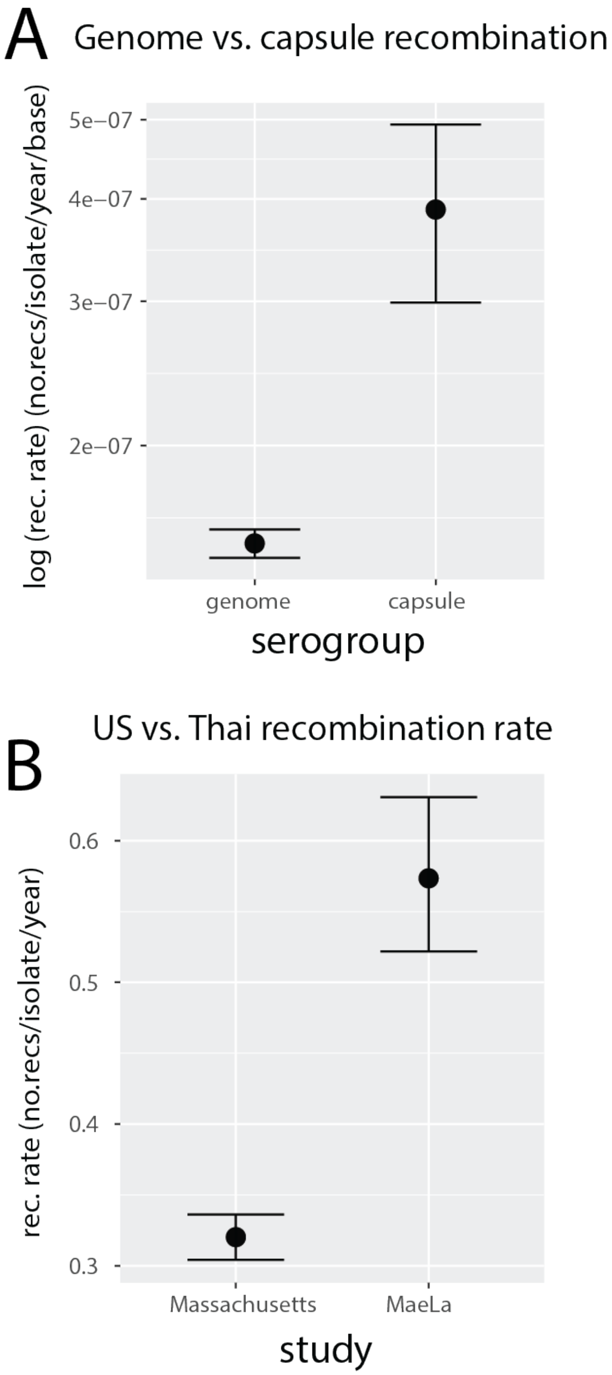

Figure S39. Comparison of recombination rates using whole-genome approach. (A) Comparison of mean recombination rate estimated in the entire genome excluding the capsular locus vs. recombination rate estimated in the capsular locus. For the comparison we used all available lineages which had a viable capsular locus. (B) Comparison of the overall genomic recombination rate estimated in Thailand (MaeLa) vs. in US (Massachusetts). All Thai and US lineages were used, however the highly recombinogenic non-typeable lineages (SC12 in US study and BC3 in Thai study) were excluded. See also Table S5.

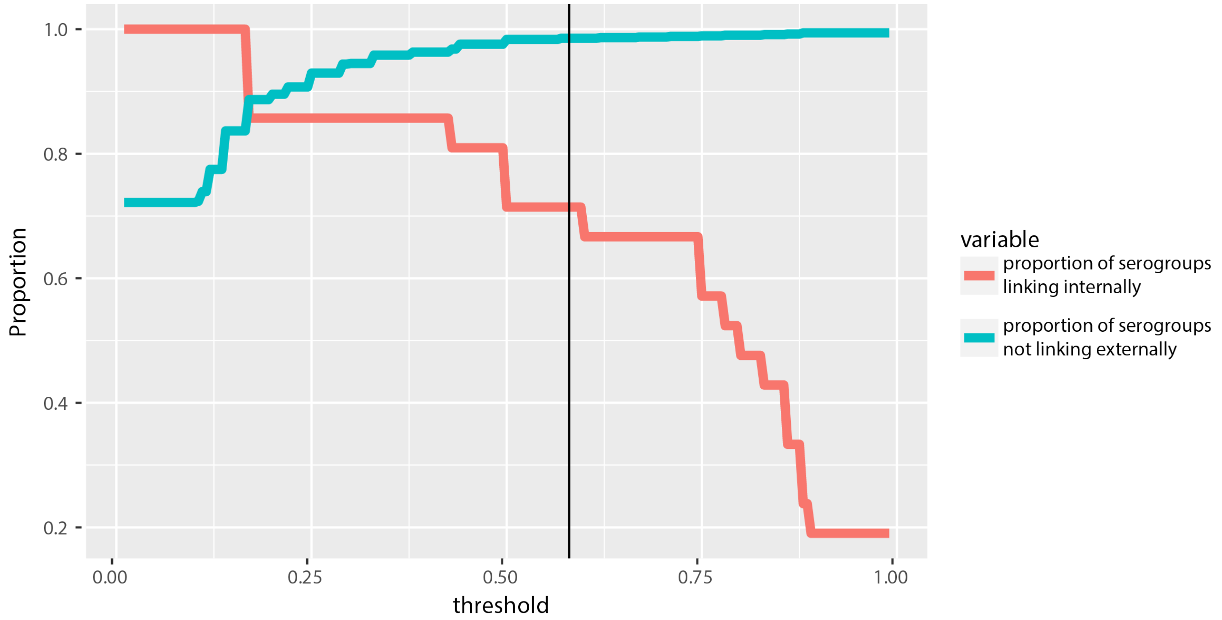

Figure S40. Choice of threshold to determine genetic serogroups. Proportion of serogroups linking internally (red) and proportion of serogroups not linking externally (blue) as a function of threshold s defined in the main text (value $s = 0.58$, marked by vertical black line, was used to define 'genetic serogroups' in figure 2). In this analysis, serogroups are here defined in the traditional sense, namely by the serologically-derived names (serogroups 1, 2, 3, 4, 5, 6, 7, etc.). Proportion of serogroups linking internally was calculated as the number of serogroups with more than one serotype which formed a connected component to the total number of serogroups with more than one serotype. Proportion of serogroups not linking externally was defined as one minus the proportion of the number of different serogroup pairs which were connected to the total number of different serogroup pairs; a connection between two different serogroups was defined as at least one pair of serotypes from these two serogroups which were linked.

Table S1. List of all *S. pneumoniae* **isolates used in this study**. See first tab of the supplementary XLSX file.

Table S2. List of all non-pneumococcal isolates used in this study. See second tab of the supplementary XLSX file.

**synteny group**	**description**
6A/6B/6F/6G	Serotypes 6A and 6B were distinguished based on a single mutation in *wciP* (S195N) which was reported to alter the rhamnose-ribtol linkage [2,3]. If 6A had a single mutation in *wciN* gene (A150T) it was classified as 6F; if 6B had a double mutation in the same gene (A150T and D38N) it was classified as 6G.
6C/6D	Same difference as between serotypes 6A and 6B [3,4].
7A/7F	Serotype 7A, unlike 7F, has an inactive *wcwD* gene due to a frameshift [5].
7B/7C/40	Undetermined; closest hit to reference [1].
9A/9V	Inactive *wcjE* gene in 9A [6,7].
9L/9N	Similarity to diverse *wcjA* gene [8].
11A/11D/11E/11F	11F has inactive copy of *gct* due to frameshift mutation; 11E has deletion in *wcjE*; 11D has S112N mutation in *wcrL* [9,10].
11B/11C	11B has inactive copy of *gct* due to frameshift mutation [9].
12A/12B/12F/44/46	Undetermined; closest hit to reference [1].
15B/15C	15C has an inactive *wciZ* gene [11].
18B/18C	18B has an inactive *wciX* gene.
19A/19F	Very diverse; distinguished based on sequence similarity.
24B/24F	24B has inactive *abp1* gene [5].
25A/25F/38	38 differs from 25A/25F by sequence similarity; as reference sequences of 25A and 25F were identical in the serotype-specific region, and since their structures have not been described, we used 25A/F classification in this study.
28A/28F	Undetermined; closest hit to reference [1].
32A/32F	Serotype 32A was distinguished form 32F by an extra chain of 18 amino-acids in the acetyltransferase gene *wcyH*.
33A/33F/37	Serotype 37, being an inactive remnant of 33A/33F, was classified based on sequence similarity of the serotype-specific genes, while 33A was distinguished from 33F by an inactive version of the *wcjE* gene [5].
35A/35C/42	35A had inactive *wcrK* gene, which distinguished it from 35C and 42; the latter two serotypes were, in the absence of contrary evidence, distinguished by the only non-synonymous substitution in the *wcrI* gene of the reference sequence, i.e., R152Q in serotype 42 [5].

Table S3. Serotyping assay. The table describes the *in silico* serotyping assay used in this study with the relevant citations.

serogroup	n	n_{non}	clonal div	no countries	cap. thickness	no clonal complexes
6	757	169	0.00149	13	595	31
19	825	220	0.000843	17	695	24
23	572	127	0.000681	19	650	12
14/15	451	158	0.00162	3	340	9
18	45	19	0.00204	2	700	7
10	100	34	0.000584	2	unknown	5
11	111	40	0.000275	2	530	4
9	58	24	0.00157	2	440	5
34/35	173	50	0.000994	2	unknown	11
16/28	62	25	0.00082	2	unknown	9
29/35	81	35	0.000647	2	520	6
22	40	20	0.000162	2	unknown	4

Table S4. Metadata obtained for each serogroup. Number of all isolates is denoted by n, while number of genetically non-identical isolates is denoted by n_{non}. Clonal diversity for each serogroup was calculated as the mean, within-population, pairwise Kimura K80 distance having removed recombinations (on non-identical subset). The capsule thickness was obtained from [25].

lineage	no. isolates	predominant ST	serotypes	predominant ser.	no. countries
PMEN1	241	81	23F-19F-19A-15B-6A-3	23F	22
PMEN2	189	90	6B-6A-6G	6B	13
PMEN14	175	4414	19F-19A-23F	19F	13
MA-1	52	460	35F-6A-10A		1
MA-2	48	62	11A	11A	1
MA-3	25	63	15A-19A	15A	1
MA-4	21	433	22F	22F	1
MA-5	15	156	9V-19A-15C-11A-15B		1
MA-6	28	338	23A-6B-15C-23B-15B-23F-6C		1
MA-7	10	191	7F	7F	1
MA-8	98	199	19A-15B-15C-7C		1
MA-9	56	439	23A-23B-23F-18C		1
MA-10	26	1390	6C	6C	1
MA-11	46	558	35B	35B	1
MA-12	10	448	NT	NT	1
MA-13	19	1876	6A-6C-6B	6A	1
MA-14	12	376	6A	6A	1
MA-15	25	320	19F-19A		1
MaeLa-1	364	4414	19F	19F	1
MaeLa-2	213	802	23F	23F	1
MaeLa-3	202	4133	NT-14		1
MaeLa-4	126	315	6B-6A	6B	1
MaeLa-5	106	172	23F-23A-24F		1
MaeLa-6	102	4209	15B-15C		1
MaeLa-7	101	63	14-15A-19F	14	1

Table S5. Whole-genome lineages used in this study. Table gives the list of twenty five whole-genome lineages used in this study. Annotation is as follows: 'MA' = Massachusetts collection, 'MaeLa' = Thai collection, 'ST' = sequence type, 'NT = non-typeable. Predominant sequence types were defined as the most common STs, and predominant serotypes were defined as those present in at least 80% of all isolates.

gene name	serotypes
rmlD	1, 2, 6A, 6C, 6D, 6G, 7C, 16F, 18A, 19A, 19B, 19F, 23A, 23B, 23F, 24B, 27, 28A, 28F, 32A, 32F, 41A, 45
pgm	3
fnlC	4, 5, 12A, 12B, 12F, 44, 46
glf	6B, 6F, 7A, 7B, 7F, 10A, 10B, 13, 16A, 17A, 17F, 18B, 18C, 18F, 19C, 22A, 22F, 24A, 24F, 29, 33B, 33D, 35B, 35F, 40, 41F, 48, 10X, 39X
ugd	8, 25A/F, 38
wcjE	9A, 9L, 9N, 9V, 11A, 11D, 11E, 11F, 15F, 20, 31, 33A, 33F, 35A, 35C, 37, 42, 43, 47A, 47F
wcrH	10C, 10F, 36
gct	11B, 11C
wciY	14
gtp3	15A,15B,15C
wcyO	21, 33C, 34, 39

Table S6. Flanking genes for different serotypes used in this study. The table shows the list of genes which were assumed as the downstream flanking genes in reference *cps* sequences as well as all sequences extracted from whole-genome data. The upstream flanking gene was always *wzg*, except for serotypes 25A/F and 38 in which case it was *glf*.

References

[1] Bentley SD, Aanensen DM, Mavroidi A, Saunders D, Rabbinowitsch E, et al. (2006) Genetic analysis of the capsular biosynthetic locus from all 90 pneumococcal serotypes. PLoS Genet 2: e31.

[2] Mavroidi A, Godoy D, Aanensen DM, Robinson DA, Hollingshead SK, et al. (2004) Evolutionary genetics of the capsular locus of serogroup 6 pneumococci. J Bacteriol 186: 8181–8192.

[3] Bratcher PE, Kim KH, Kang JH, Hong JY, Nahm MH (2010) Identification of natural pneumococcal isolates expressing serotype 6D by genetic, biochemical and serological characterization. Microbiology (Reading, Engl) 156: 555–560.

[4] Park IH, Pritchard DG, Cartee R, Brandao A, Brandileone MC, et al. (2007) Discovery of a new capsular serotype (6C) within serogroup 6 of Streptococcus pneumoniae. J Clin Microbiol 45: 1225–1233.

[5] Mavroidi A, Aanensen DM, Godoy D, Skovsted IC, Kaltoft MS, et al. (2007) Genetic relatedness of the Streptococcus pneumoniae capsular biosynthetic loci. J Bacteriol 189: 7841–7855.

[6] Calix JJ, Oliver MB, Sherwood LK, Beall BW, Hollingshead SK, et al. (2011) Streptococcus pneumoniae serotype 9A isolates contain diverse mutations to wcjE that result in variable expression of serotype 9V-specific epitope. J Infect Dis 204: 1585–1595.

[7] Calix JJ, Saad JS, Brady AM, Nahm MH (2012) Structural characterization of Streptococcus pneumoniae serotype 9A capsule polysaccharide reveals role of glycosyl 6-O-acetyltransferase wcjE in serotype 9V capsule biosynthesis and immunogenicity. J Biol Chem 287: 13996–14003.

[8] McEllistrem MC (2009) Genetic diversity of the pneumococcal capsule: implications for molecular-based serotyping. Future Microbiol 4: 857–865.

[9] Calix JJ, Nahm MH, Zartler ER (2011) Elucidation of structural and antigenic properties of pneumococcal serotype 11A, 11B, 11C, and 11F polysaccharide capsules. J Bacteriol 193: 5271–5278.

[10] Oliver MB, Jones C, Larson TR, Calix JJ, Zartler ER, et al. (2013) Streptococcus pneumoniae serotype 11D has a bispecific glycosyltransferase and expresses two different capsular polysaccharide repeating units. J Biol Chem 288: 21945–21954.

[11] van Selm S, van Cann LM, Kolkman MA, van der Zeijst BA, van Putten JP (2003) Genetic basis for the structural difference between Streptococcus pneumoniae serotype 15B and 15C capsular polysaccharides. Infect Immun 71: 6192–6198.

[12] van Tonder AJ, Bray JE, Roalfe L, White R, Zancolli M, et al. (2015) Genomics Reveals the Worldwide Distribution of Multidrug-Resistant Serotype 6E Pneumococci. J Clin Microbiol 53: 2271–2285.

[13] Burton RL, Geno KA, Saad JS, Nahm MH (2016) Pneumococcus with the "6E" cps Locus Produces Serotype 6B Capsular Polysaccharide. J Clin Microbiol 54: 967–971.

[14] Bratcher PE, Park IH, Oliver MB, Hortal M, Camilli R, et al. (2011) Evolution of the capsular gene locus of Streptococcus pneumoniae serogroup 6. Microbiology (Reading, Engl) 157: 189–198.

[15] McEllistrem MC, Nahm MH (2012) Novel pneumococcal serotypes 6C and 6D: anomaly or harbinger. Clin Infect Dis 55: 1379–1386.

[16] Park IH, Park S, Hollingshead SK, Nahm MH (2007) Genetic basis for the new pneumococcal serotype, 6C. Infect Immun 75: 4482–4489.

[17] Elberse K, Witteveen S, van der Heide H, van de Pol I, Schot C, et al. (2011) Sequence diversity within the capsular genes of Streptococcus pneumoniae serogroup 6 and 19. PLoS ONE 6: e25018.

[18] Zahner D, Gandhi AR, Yi H, Stephens DS (2011) Mitis group streptococci express variable pilus islet 2 pili. PLoS ONE 6: e25124.

[19] Kilian M, Riley DR, Jensen A, Bruggemann H, Tettelin H (2014) Parallel evolution of Streptococcus pneumoniae and Streptococcus mitis to pathogenic and mutualistic lifestyles. MBio 5: e01490–01414.

[20] Yang J, Nahm MH, Bush CA, Cisar JO (2011) Comparative structural and molecular characterization of Streptococcus pneumoniae capsular polysaccharide serogroup 10. J Biol Chem 286: 35813–35822.

[21] Salter SJ, Hinds J, Gould KA, Lambertsen L, Hanage WP, et al. (2012) Variation at the capsule locus, cps, of mistyped and non-typable Streptococcus pneumoniae isolates. Microbiology (Reading, Engl) 158: 1560–1569.

[22] Mostowy R, Croucher NJ, Andam CP, Corander J, Hanage WP, et al. (2017) Efficient inference of recent and ancestral recombination within bacterial populations. Mol Biol Evol in press.

[23] Excoffier L, Dupanloup I, Huerta-Sanchez E, Sousa VC, Foll M (2013) Robust demographic inference from genomic and SNP data. PLoS Genet 9: e1003905.

[24] Rambaut A, Grassly NC (1997) Seq-Gen: an application for the Monte Carlo simulation of DNA sequence evolution along phylogenetic trees. Comput Appl Biosci 13: 235–238.

[25] Weinberger DM, Trzcinski K, Lu YJ, Bogaert D, Brandes A, et al. (2009) Pneumococcal capsular polysaccharide structure predicts serotype prevalence. PLoS Pathog 5: e1000476.
